# Genome-Wide Effects on Gene Expression Between Parental and Filial Generations of Trisomy 11 and 12 of Rice

**DOI:** 10.1186/s12284-023-00632-5

**Published:** 2023-03-25

**Authors:** Shang Sun, Kai Liu, Chao Xue, Yingying Hu, Hengxiu Yu, Guoxiao Qi, Jijin Chen, Xiya Li, Xinru Zhao, Zhiyun Gong

**Affiliations:** 1 Jiangsu Key Laboratory of Crop Genomics and Molecular Breeding/Key Laboratory of Plant Functional Genomics of the Ministry of Education/Jiangsu Key Laboratory of Crop Genetics and Physiology, Agricultural College of Yangzhou University, Yangzhou, 225009 China; 2 Jiangsu Co-Innovation Center for Modern Production Technology of Grain Crops, Yangzhou University, Yangzhou, 225009 China

**Keywords:** Aneuploid, Gene expression, Rice

## Abstract

Aneuploid refers to the gene dosage imbalance due to copy number alterations. Aneuploidy is generally harmful to the growth, development and reproduction of organisms according to the numerous research. However, it has rarely been reported on whether aneuploid have a relevant pattern of genome expression between the parental and its offspring generations. In this study, mRNA sequencing analysis was performed on rice (*Oryza sativa* L.) primary trisomes 11 and 12, same primary trisomes and normal individuals in their filial generation. We systematically summarized the changes in gene expression patterns that occur on *cis *genes and on *trans *genes between parental and filial generations. In T11 and T12, the ratio of *cis*-gene expression showed intermediate type in parents and dosage compensation in filial generations, which maybe due to more genes being downregulated. The *trans* genes were also affected by aneuploidy and manifested as *cis*-related. The strains with normal chromosomes in filial generations, there are still aneuploid-sensitive genes differentially expressed in their genomes, indicating that the effect of aneuploidy is far-reaching and could not be easily eliminated. Meanwhile, among these differentially expressed genes, genes with low-expression level were more likely to be upregulated, while genes with medium- and high-expression level were easy to be downregulated. For the different types of rice aneuploid, upregulated genes were mainly associated with genomic imbalance while downregulated genes were mainly influenced by the specific added chromosome. In conclusion, our results provide new insights into the genetic characterization and evolution of biological aneuploidy genomes.

**Supplementary Information:**

The online version contains supplementary material available at 10.1186/s12284-023-00632-5.

## Background

Aneuploidy refers to numerical chromosomal abnormalities, which is caused by chromosome mismatches during meiosis or mitosis and disrupts the balance of the entire genome (Birchler and Newton 1981; Huettel et al 2008; Torres et al 2010; Chen et al 2019). In general, this genomic instability (aneuploidy) has more serious phenotypic consequences than that changes in the number of entire genomes (ploidy) (Hou et al 2018). Studies of gene expression in aneuploids in a variety of species have claimed many different types of responses (Birchler 2010). Aneuploidy affects the physiological process and normal cell division in the budding yeast cells (Torres et al 2007). Trisomy of chromosome 12 is common in human pluripotent stem cells (hPSCs). Global gene expression analyses reveal that trisomy 12 profoundly affects proliferation, differentiation and apoptosis (Ben-David et al 2014). In *Arabidopsis thaliana*, global transcript profiles of normal diploids and chromosome 5 trisomy analysis showed that trisomy 5 would disrupt genome-wide transcription and expression (Huettel et al 2008). Aneuploidy decreases not only organismal but also cellular fitness and elicits traits that are shared between different aneuploid mammalian cells (Williams et al 2008).

Aneuploidy is also a hallmark of cancer, a disease of enhanced proliferative capacity. Compared with normal cells, chromosomal variants are common in cancer cells, is more of a marker of tumorigenic (Cao et al 2008). However, aneuploid does not produce all bad things, it may be a suppressor of tumors (Vasudevan et al 2021). In some species, aneuploidy can provide a means of gaining or losing tumorigenic and tumor suppressor genes, and aneuploidy may be a stress response that can promote cell growth and proliferation (Torres et al 2007). In addition, in the liver and brain tissue, aneuploidy is necessary for cell function, the relative effect of aneuploidy cells might evolve a new mechanism to aneuploidy adaptation (Sheltzer and Amon 2011), which may be advantageous in certain circumstances.

Further analysis of individual genes suggests that effects of aneuploidy can be divided into two types: *cis*-effect and *trans*-effect (Devlin et al 1982; Veitia et al 2008; Birchler 2010; Zhang et al 2017). *Cis*-effect can be divided into two types according to the change of gene expression on extra chromosomes: dosage effect and dosage compensation effect. Gene dosage effects reveal that the doses of many genes have been changed in aneuploidy cells, which has a commonly negative effect on organism growth and development. The degree of phenotypic variation related to different levels of gene expression in aneuploid (Lambert et al 1976; Sheltzer et al 2011). On the other hand, the aneuploidy of certain organisms will also provide a natural, transient phenotypic evolutionary pathway to buffer the effects of gene amplification, implying that there is a self-balancing regulatory mechanism in these abnormal individuals, in which the dosage of structural genes changes while the expression level remains the same, and that is the dosage compensation effect (Birchler 2010). In maize aneuploid, the dosage of chromosomal segments was added, but the transcript level of most genes encoded therein did not change, showing dosage compensation (Guo and Birchler 1994). In Drosophila, the predominant response of genes was dosage compensation in that similar expression occurred in the trisomic compared with the normal diploid (Devlin et al 1982; Sun et al 2013b, 2013a). Observing and analyzing the degree of changes in the abundance of gene expression on aneuploid chromosomes (*cis*-effect) and the changes in gene expression on all remaining normal chromosomes (*trans*-effect) may answer the potential molecular mechanisms underlying the occurrence of aneuploid events to some extent. The *trans*-effect can be classified into two types based on the change in gene expression on the remaining chromosomes: direct *trans*-effect and inverse *trans*-effect. The direct *trans*-effect is that the expression level of *trans* genes was positively correlated with the direction of aneuploid, if shown negatively, it is the inverse *trans*-effect (Zhang et al 2017; Birchler 2014).

Plants generally tolerate aneuploidy better than animals, so plants have traditionally provided excellent systems for studying aneuploidy (Huettel et al 2008). Aneuploidy global transcript profiles can be constructed in hexaploid wheat (*Triticum aestivum*), maize and *Arabidopsis thaliana*, revealing additional and novel features of global gene expression alterations that would result from *cis* and *trans* regulation. These studies all provide a systematic summary of the implications for how gene expression operates, the evolution of duplicated genes, and the potential basis for quantitative traits (Hou et al 2018; Zhang et al 2017; Shi et al 2021; Johnson et al 2020). However, most of those studies only described the genome information of one generation, which cannot summarize well about sustained effects of aneuploidy on the genetics and evolution of organisms. Rice is a model plant for molecular biological study in monocots (Goff 1999). It is relatively easy to prepare well-spreading pachytene or premetaphase chromosomes using rice anthers or roots, which makes it a good system for aneuploidy study as well (Cheng et al 2001a, 2001b). In this study, different types of rice trisomy from different generations with a common genetic background of Zhongxian 3037 were systematically studied. According to the detection of global gene expression changes by mRNA-seq (mRNA-sequencing) to unravel novel features with the two major impacts of aneuploidy (*cis*- and *trans*-effects) as well as dosage effect and dosage compensation. Summarizing the dynamic changes of aneuploidy effects during the transmission from parental to filial generation and the gene expression patterns of aneuploid genomes between generations. The result provides a theoretical basis for further analysis of how genomic imbalance due to aneuploidy affects and regulates the mechanisms of organism growth and development.

## Materials and Methods

### Plant Materials

All plant materials used for the study were in the same genetic background of rice Zhongxian 3037 (*Oryza sativa* L. 2*n* = 24). The aneuploids were as follows: parental primary trisomy 11 (2*n* = 25, designated as T11-P) and parental primary trisomy 12 (2*n* = 25, designated as T12-P), filial primary trisomy 11 (2*n* = 25, designated as T11-F) and filial primary trisomy 12 (2*n* = 25, designated as T12-F), plants with normalized chromosome numbers in the progeny population of the parental primary trisomy 11 and 12 (2*n* = 24, designated as T11-FN and T12-FN). All materials were grown under the same field environmental conditions. All collected leaf tissues were stored at -80 °C.

### Karyotyping

All aneuploidy strains were identified cytologically using Oligo-FISH, in which dual-color bar-code oligo probes and chromosome painting probes were used for karyotype analysis of the parental and filial generation to ensure the accuracy of the results. The protocols were largely as described originally (Liu et al 2020). Each FISH image was acquired using Olympus BX60 microscope and processed using Adobe Photoshop 2020.

### RNA Extraction and mRNA-Seq Analysis

Total leaf RNA from diploid (Zhongxian 3037, T11-FN, T12-FN) and aneuploids (T11-P, T11-F, T12-P and T12-F) seedlings were isolated using the RNA simple Total RNA Kit (Tiangen; China), according to the manufacturer’s protocols. mRNA sequencing was performed on the Illumina Nova Seq 6000 with PE150 method. Two biological replicates of each sample were sequenced. Raw data was filtered by Trimmomatic (version 0.38) (Bolger et al 2014) to remove adapters and low-quality reads. Clean reads were mapped to the rice genome (MSU 7.0) with TopHat2 (version 2.1.1) (Kim et al 2013) and only uniquely mapped reads (Phred Quality Score > 20) were analyzed next. The data information and mapping efficiency are shown in (Additional file 2: Table S1). Fragments per kilobase of exon per million mapped fragments (FPKM) values of rice annotated genes were calculated using Cufflinks and differential expression analysis was performed using Cuffdiff in Cufflinks (version 2.2.1) (Trapnell et al 2010). Genes with *p*-value ≤ 0.05 and fold-change ≥ 1.50 in the comparison of aneuploidy with normal diploids were considered as differentially expressed genes (DEGs).

### RT-qPCR

Total RNA was obtained from the seedling leaves by using the RNA simple Total RNA Kit (Tiangen; China). cDNA was synthesized from 3 μg of total RNA by using the reverse transcription kit (Tiangen; China). Real-time PCR (RT-qPCR) was performed using the SYBR qPCR Master MIX (Vazyme; China) and Bio-Rad CFX96TM real-time PCR system. The rice ubiquitin gene (*UBQ*) was selected as the internal reference gene for RT-qPCR. The primer sequence of RT-qPCR genes has also been provided (Additional file 3: Table S2).

### Data Statistics and Visualization

All statistical analyses were performed based on R (R version R 4.0.2 and Rstudio version 1.2.1335). We calculated Pearson correlation coefficients between two biological replicates of aneuploidy in the range of 0.90–0.99 using FPKM of genes in R (Additional file 2: Table S1).

We defined the mean value of gene FPKM between two biological replicates as the expression level of the gene and generated a ratio distribution map using genes with FPKM > 0. Clustered heatmaps were generated as described in previous studies (Zhang et al 2017). In brief, hierarchical clustering of aneuploid strains and their normal diploids was performed using a total of 9,594 genes that showed dysregulated expression in at least one aneuploid strain compared to the normal diploids. Hierarchical clustering analysis was performed and visualized using the R package pheatmap (version 1.0.12, https://CRAN.R-project.org/package=pheatmap).

### Gene Ontology Analysis

Gene Ontology (GO) terms enrichment was performed by g:Profiler with an FDR < 0.05 (Reimand et al 2016). GO results were clustered REVIGO (Supek et al 2011).

## Results

### Karyotype Analysis Different Types of Rice Primary Trisomy

**Fig. 1:**
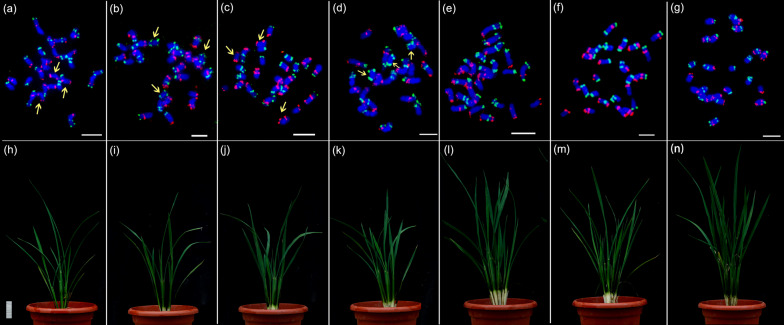
Fluorescence in situ hybridization (FISH) identification of aneuploid and diploid on mitotic metaphase chromosomes in rice. **a** and **b** Bar-code oligo-FISH analysis of parental primary trisomy 11 and 12 (named as T11-P and T12-P) using FAM-green and digoxigenin-red labeled probes on somatic cell chromosomes in mitotic metaphase, respectively. **c** and **d** Bar-code oligo-FISH analysis of filial primary trisomy 11 and 12 (named as T11-F and T12-F) using FAM-green and digoxigenin-red labeled probes on somatic cell chromosomes in mitotic metaphase, respectively. **e** and **f** FISH analysis of normal strain of progeny of parental primary trisomy 11 and 12 (named as T11-FN and T12-FN) using FAM-green and digoxigenin-red labeled probes on mitotic metaphase chromosomes, respectively. **g** FISH analysis Zhongxian 3037 using FAM-green and digoxigenin-red labeled probes on mitotic metaphase chromosomes. **h–n** The seedling phenotype corresponding to (**a–g**). Bars = 5 cm. The yellow arrow points to the extra chromosomes. Chromosomes were counterstained with 4’,6-diamidino-2-phenylindole. Bars = 5 μm

Due to the additive effect of the extra chromosomes, different types of trisomy showed various morphology (Blakeslee et al 1920). However, it is inaccurate to distinguish the types of trisomy only by morphological traits, cytological identification is required to determine the type of chromosome added to each trisomy (Khush et al 1984). Here, we identified the chromosomal composition of trisomic plants using “Bar code” oligo-FISH (Liu et al 2020). Based on these methods, we obtained 6 rice karyotypes: parental primary trisomy 11 (named as T11-P, 2*n* = 25) (Fig. 1a and h), parental primary trisomy 12 (named as T12-P, 2*n* = 25) (Fig. 1b and i), filial primary trisomy 11 (named as T11-F, 2*n* = 25) (Fig. 1c, j), filial primary trisomy 12 (named as T12-F, 2*n* = 25) (Fig. 1d and k), and the diploid plants with normal chromosomes in the sexual offspring trisomy 11 and 12, respectively (named as T11-FN and T12-FN, 2*n* = 24) (Fig. 1e, l and f, m). The wild type provides reference as standard karyotype (Diploid, 2*n* = 24) (Fig. 1g and n). The difference between the rice primary trisomy and the diploid can be found through the phenotype of the seedling stage. The plant height of both parental and filial primary trisomy was shorter than that of the diploids. For each type, we obtained at least three individual plants for subsequent experiments. The karyotypes of all the individual plants used in this experiment were also supplemented with oligo-painting FISH for verification, ensuring that they do not contain any other chromosomal variations (Additional file 1: Fig. S1). We also measured the phenotypes on agronomic traits during the maturity period (Additional file 1: Fig. S2).

### *Different Types of Rice Aneuploid Regulated Gene Expression *Via *Cis- and Trans-Effect*

Gene expressions are abnormal in aneuploidy genomes (Birchler 2019). By mRNA-seq, we analyzed the transcriptome of all the aneuploid (2*n* = 25) and the diploid (2*n* = 24) leaves at seedling stage under the same growth environment conditions. Only unique reads were selected for further analysis, and 31.39–41.51 million reads from all samples were mapped to the rice genome. Pearson correlation coefficients from two biological replicates of each type were 0.90–0.99 (Additional file 2: Table S1), indicating that the data were reliable and reproducible. RT-qPCR with eight randomly selected DEGs was conducted to validate the results of the mRNA-seq data (Additional file 1: Fig. S3). Relationships between dosage and expression are consistent with mRNA-seq and RT-qPCR, which further proved the reliability of mRNA-seq data.

**Fig. 2:**
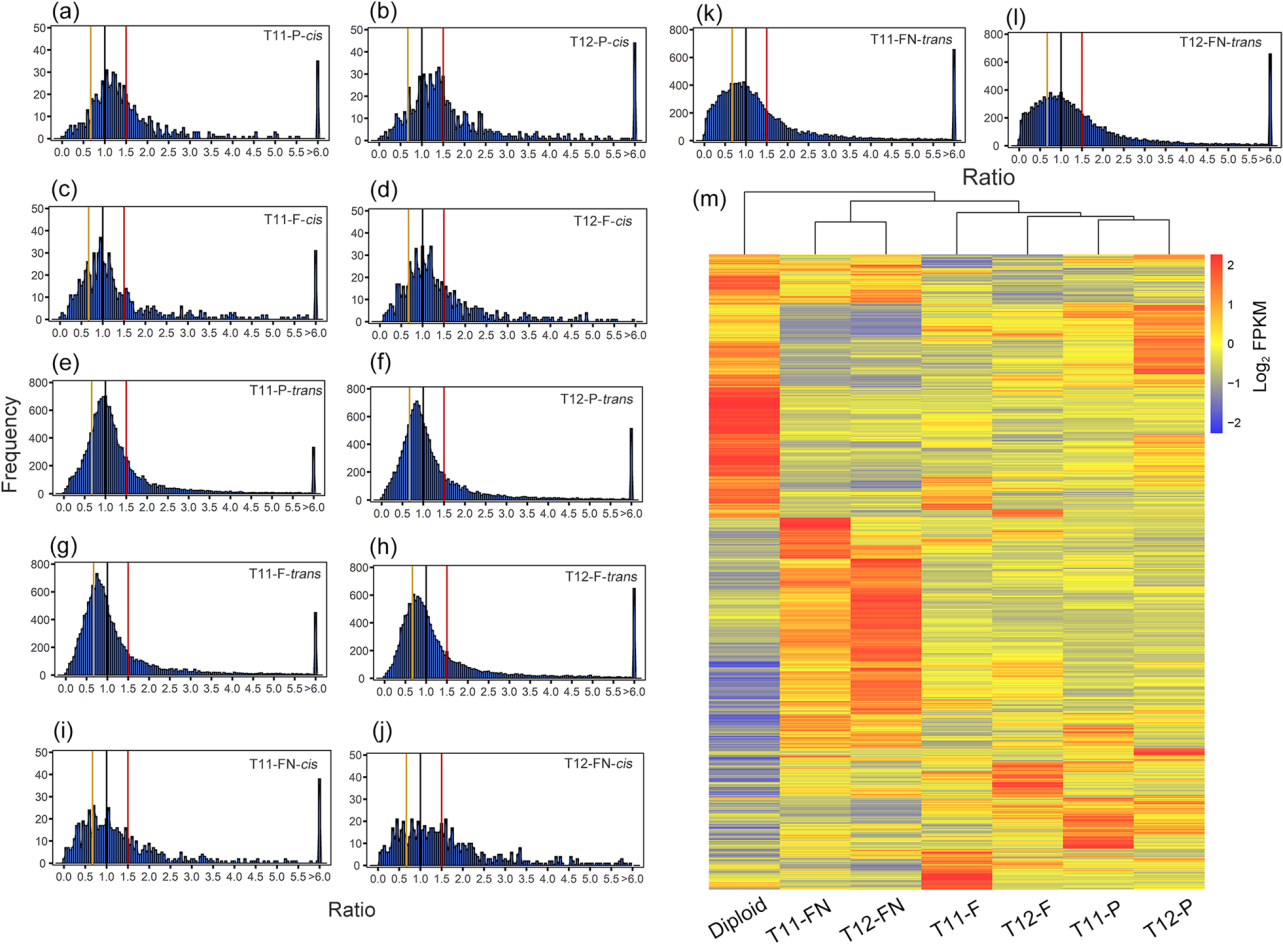
Ratio distribution of gene expression levels of parental and filial of aneuploid compared with diploid. **a**–**d** The distribution of gene expression ratios on the extra chromosomes (*cis*) of parental, filial primary trisomy 11 and 12, respectively. **e**–**h** The distribution of gene expression ratios on the other chromosomes (*trans*) of parental, filial primary trisomy 11 and 12, respectively. **i** and **j** The distribution of gene expression ratios on the extra chromosomes of T11-FN and T12-FN, respectively. **k** and **l** The distribution of gene expression ratios on the other chromosomes of T11-FN and T12-FN, respectively. The ratios were plotted with bins of 0.05 increments. X-axis represents the ratio bin of fold-change in gene expression levels and Y-axis represents the expressed gene (FPKM > 0) frequency per bin. A ratio of 1.00 and 1.50 represents the dosage compensation and dosage effect in *cis*, respectively. A ratio of 0.67 represents the inverse ratio of gene expression in *trans*. These ratio values are demarcated with labeled vertical lines in red (1.50), black (1.00) and yellow (0.67). **m** Hierarchical analysis of dysregulated genes from aneuploid and diploid was performed based on the expression patterns of genes showed dysregulated expression in at least one aneuploid (9,594 in total). Heatmap visualizing the hierarchical clustering of gene expression levels in each strains

For aneuploidy genome, expressions patterns of genes located on the extra chromosomes (*cis*-effect) and located on the other normal chromosomes (*trans*-effect) have different distributions, and the gene expression pattern is synergistically regulated (Birchler 1981). Based on this, we analyzed whole genome-wide gene expression patterns of *cis* and *trans* in rice aneuploid. Comparing aneuploid to diploid, the main peaks of gene expression levels regulated by *cis*-effect in both T11-P and T12-P exhibited intermediate (1.00–1.50-fold, Fig. 2a and b). The *cis*-effect of T11-F and T12-F are consistent with parental primary trisomy, but its main peak is more inclined to dosage compensation (1.00-fold, Fig. 2c and d). The *trans*-effect of parental aneuploid exhibited the most common trend that reduced expression in the aneuploid compared with diploid, with ratios between 0.67- and 1.00-fold (Fig. 2e and f). The main peaks of *trans* genes in filial aneuploid showed further close to 0.67-fold (Fig. 2g and h). The pattern can be summarized in the T11-FN and T12-FN, where both the *cis* and *trans* peaks tend to be flatter in modulation range, and none of the *cis*-gene expression ratios of T11-FN and T12-FN have a peak as sharp as the primary trisomy (Fig. 2i and j). Theoretically, the main *trans* peaks of T11- and T12-FN should have expression ratio of around 1.00-fold, but which are all to the left of 1.00-fold (Fig. 2k and l). These results preliminarily suggested that parental aneuploidy affects the gene transcription of normal individuals in the offspring.

In exception to *cis*- and *trans*-effect, the differential effects of different types aneuploid on gene expression were also reflected in a hierarchical analysis of overall expression patterns versus expression differences in diploid (Bin et al 2019; Makarevitch and Harris 2010; Wu et al 2018). The hierarchical analysis was used to identify differences in expression patterns between the different types of rice aneuploid and diploid (Fig. 2m). The results showed that the overall expression pattern was varied most in the diploid. Different types of rice aneuploid exhibited similar distributions at the transcriptional level, such as T11-P and T12-P, T11-F and T12-F, T11-FN and T12-FN clustered together and formed a branch. This implies that the expression pattern of the aneuploid genome does not depend on a specific added chromosome, but is guided by dosage changes in the genome. Interestingly, the gene expression patterns did not resemble those of the wild type diploid although they have exactly same karyotype with wild type diploid (Fig. 2m). These results shown that parental aneuploidy dose effect the gene expression of normal individuals in the offspring, and this effect is not regulated by the type of aneuploidy (chromosome-independent).

### A Similar Whole-Genomic Transcription Patterns Exists in Different Types of Aneuploid

**Fig. 3:**
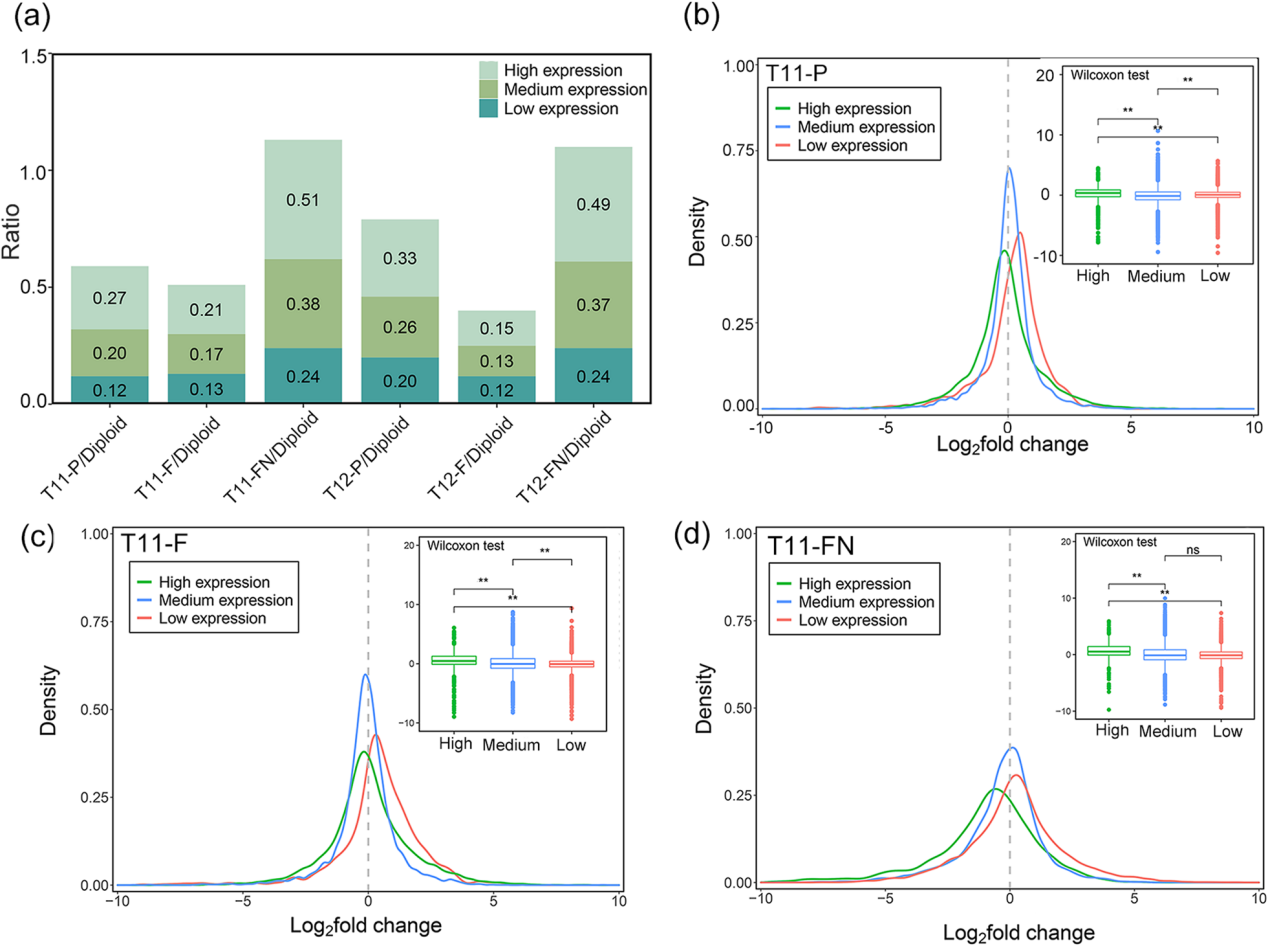
Impact of extra chromosomes on changes in gene expression. **a** Proportions of DEGs with different expression levels in T11 (T11-P, T11-F and T11-FN) and T12 (T12-P, T12-F, T12-FN). Based on the expression levels of Zhongxian 3037, genes were divided into three clusters: (i) high-expression genes (FPKM > 100), (ii) medium-expression genes (10 < FPKM ≤ 100) and (iii) low-expression genes (0 < FPKM ≤ 10). Y-axis represents the proportion of DEGs in different expression level genes. **b**, **c** and **d** The Log_2_(fold change) frequency distribution of genes with different expression levels in T11-P, T11-F and T11-FN, respectively. X-axis represents the Log_2_(fold change) in gene expression levels between diploid and trisomy and Y-axis represents frequency distributions of Log_2_(fold change). The red, blue and green lines indicate low (0 < FPKM ≤ 10), medium (10 < FPKM ≤ 100) and high (FPKM > 100) -expression genes, respectively; ns, not significant Boxplot on the right is a statistical analysis of the expression levels of the different types of genes (Wilcoxon test, ** *p-*value < 0.01)

To further explore whether genes exhibited similar expression patterns impacted on account of the addition resulting extra chromosomes, we analyzed the transcriptional response patterns of rice aneuploid. In primary trisomy and diploid, we first screened for genes that were expressed in both of them (FPKM > 0). Next, based on the expression levels of genes in diploid, we classified the genes into three groups, which are high (FPKM > 100), medium (10 < FPKM ≤ 100), and low (0 < FPKM ≤ 10) -expression levels (Fig. 3a and Additional file 4: Table S3). In addition, the high-expression genes exhibited a relatively large impact of extra chromosomes (14.54%-50.80% of high-expression genes), the trend gradually decreases in medium-expression levels (13.07%-37.82% of medium-expression genes). We found that low-expressed genes were less sensitive to additional chromosomes and had the lowest percentage of genes (11.83%-23.90% of low-expression genes).

Another interesting pattern is that downregulated genes commonly consist in medium- and high-expression levels, while upregulated genes are mainly concentrated in low-expression levels (Fig. 3b–d and Additional file 1: Fig. S4). To substantiate that, we compared the distribution of gene expression fold-change for all pairwise comparisons in each group (Additional file 5: Table S4). For instance, by pairwise comparison in T11-P and diploid, a total of 1,411 DEGs were counted in low-expression level genes (0 < FPKM ≤ 10), among which 902 (63.93%) genes were upregulated and 509 (36.07%) were downregulated. For medium-expression level genes (10 < FPKM ≤ 100), the 1,341 DEGs contained 609 (45.41%) upregulated genes and 732 (54.59%) downregulated genes. Of the 274 DEGs in the high-expression level (FPKM > 100), 109 (39.78%) genes were upregulated and 165 (60.22%) genes were downregulated. All remaining pairwise comparisons in each group showed the largest percentage of upregulated genes to total DEGs at low-expression level, while the largest percentage of downregulated genes to total DEGs at medium- and high-expression level (*p*-value < 0.01). This indicates that aneuploidy can promote transcription of the low-expression level genes and suppress transcription of the high-expression level genes.

### Differentially Expressed Genes are Involved in Distinct Functional Categories

**Fig. 4:**
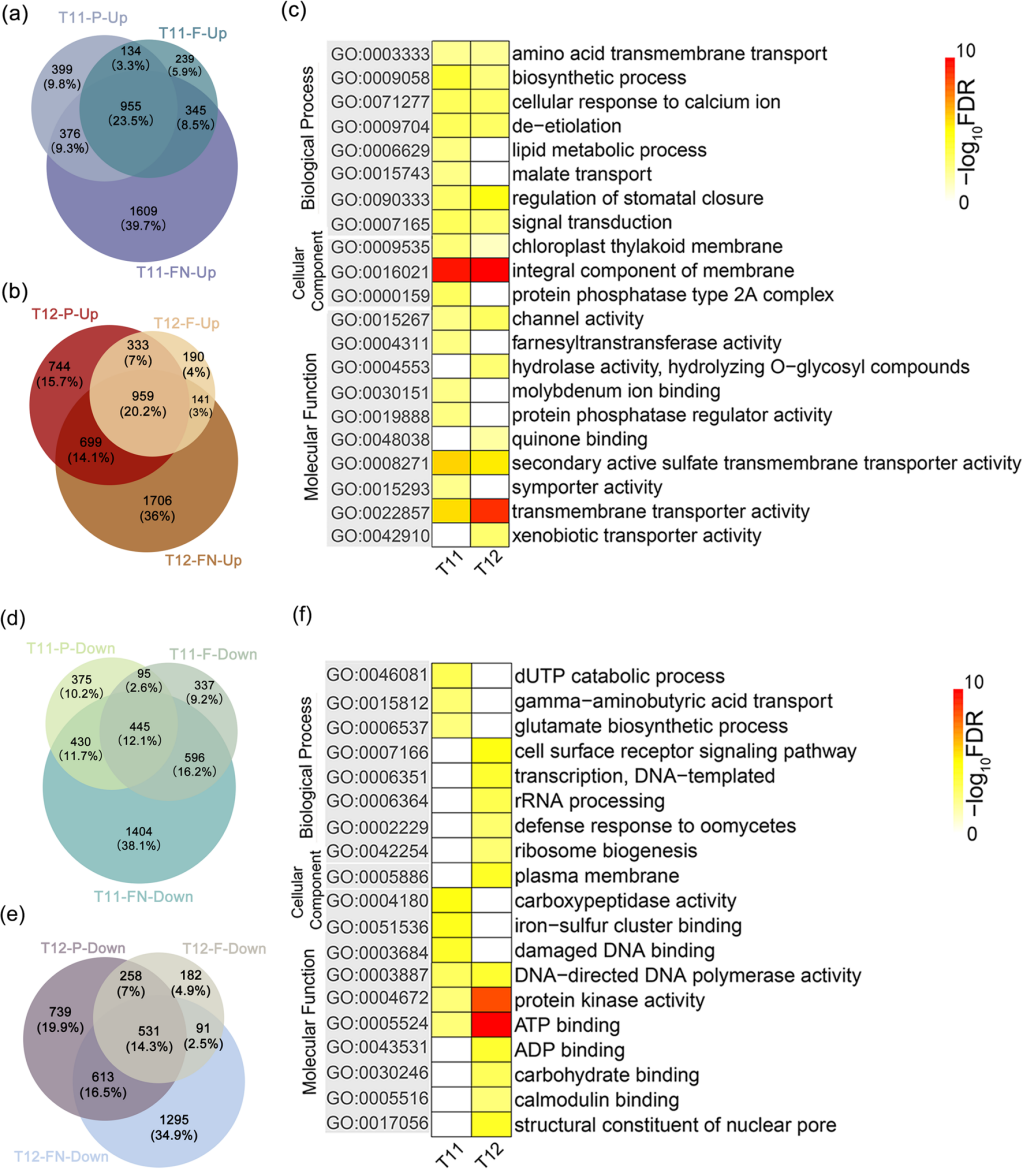
GO enrichment analysis of DEGs. **a** and **b** The venn diagrams of the upregulated genes of T11 (T11-P, T11-F and T11-FN) and T12 (T12-P, T12-F and T12-FN), respectively. **c** Heatmap of GO enrichment analysis of upregulated overlapped genes of T11 (overlapped T11-P and T11-F) and T12 (overlapped T12-P and T12-F). **d** and **e** The venn diagrams of the downregulated genes of T11 (T11-P, T11-F and T11-FN) and T12 (T12-P, T12-F and T12-FN), respectively. **f** Heatmap of GO enrichment analysis of downregulated overlapped genes of T11 (overlapped T11-P and T11-F) and T12 (overlapped T12-P and T12-F)

A large number of genes within the aneuploidy genome are differentially expressed in response to their dynamic imbalance, and whether these co-regulated or specific DEGs are involved in the same pathways in response to the occurrence of aneuploidy. Venn diagrams were constructed to determine the co-responsive genes between different types of aneuploid (Fig. 4). The overlapping of 1,864 and 1,673 upregulated genes in T11-P and T11-F, respectively, revealed that there were 1,089 genes co-upregulated (Fig. 4a). Meanwhile, a total of 1,292 genes were co-upregulated in T12-P and T12-F (Fig. 4b). GO enrichment analysis of those co-regulated genes found that the co-upregulated involved in GO terms in different types of rice aneuploid are largely similar (Fig. 4c). The co-responsive upregulated genes associated with biological processes were significantly enriched for annotated GO terms involved in biosynthetic process, cellular response to calcium ion de-etiolation, and signal transport. In terms of cellular component and molecular function, genes were overrepresented by GO terms associated with integral component of membrane and chloroplast thylakoid membrane, transporter activity, respectively. We also conducted a total of 540 co-downregulated genes in T11 and 789 co-downregulated genes in T12, respectively (Fig. 4d and e). The downregulated DEGs in T11 were mainly involved in carboxypeptidase activity, damaged DNA binding and iron-sulfur cluster binding (Fig. 4f). In T12, co-downregulated DEGs were mainly enriched in protein kinase activity, ATP binding (Fig. 4f). In both types of aneuploid, there are basically differences in GO terms of co-downregulated genes. In addition, we divided the co-regulated DEGs into *cis* and *trans* genes from T11 and T12, respectively. It can be clearly concluded that the *cis* and *trans* genes have different contributions to aneuploidy genome (Additional file 1: Fig. S5). A total of 67 co-upregulated *cis* genes in T11 (overlapped T11-P and T11-F) were only enriched in cellular components, but the 1,022 co-upregulated *trans* genes were enriched in all three types of GO categories (Additional file 1: Fig. S5a). For the co-downregulated genes in T11, we did not find significantly enriched GO categories in 28 *cis* genes, but there were 512 *trans* genes that were mainly involved in biological process and molecular function (Additional file 1: Fig. S5b). In T12 (overlapped T12-P and T12-F), a total of 86 *cis* genes and 1,206 *trans* genes were co-upregulated, 28 *cis* genes and 761 *trans* genes were co-downregulated. The *cis* genes (up- and down-regulated) were mainly enriched in molecular function and biological process while the *trans* genes (up- and down-regulated) were enriched in all three types of GO categories (Additional file 1: Fig. S5a and S5b).

We were interested whether there was a part of genes that was not affected by the dosage effect and kept the gene expression markers unchanged in the rice aneuploid genome (*p*-value ≥ 0.05). Therefore, we performed detailed statistics on this type genes. There are 909 and 923 genes in T11-P and T11-F, respectively, a high percentage (76.20%) of genes are overlapped between them (Additional file 1: Fig. S6a). The same phenomenon can be observed in T12 (Additional file 1: Fig. S6b). GO enrichment analysis revealed that the co-regulated compensatory genes in T11 and T12 involved in the GO terms are basically similar (Additional file 1: Fig. S6c), suggesting that the function of these genes share the commonalities, is that not affected by the added chromosomes.

In the previous analysis, we found a large number DEGs in T11-FN and T12-FN. As a result, venn diagrams also established overlap all the DEGs in T11-P, T11-F and T11-FN (Fig. 4a and b), and the same statistics were performed in T12 (Fig. 4d and e). The co-up and downregulated genes were significantly enriched for annotated GO terms are largely similar to those of primary trisomy (Additional file 1: Fig. S7). In addition, we also separately counted the co-regulated DEGs in T11-FN and T12-FN, containing 2,661 co-upregulated genes and 2,041 co-downregulated genes (Additional file 1: Fig. S8a and S8b). Upregulated genes were significantly enriched for annotated GO terms involved in ion transmembrane transport and small molecule metabolic process, and the downregulated genes were mainly involved in the terms of cellular nitrogen compound metabolic process (Additional file 1: Fig. S8c and S8d).

### Genes in Different Functional Groups Respond Differently to Aneuploid

**Fig. 5:**
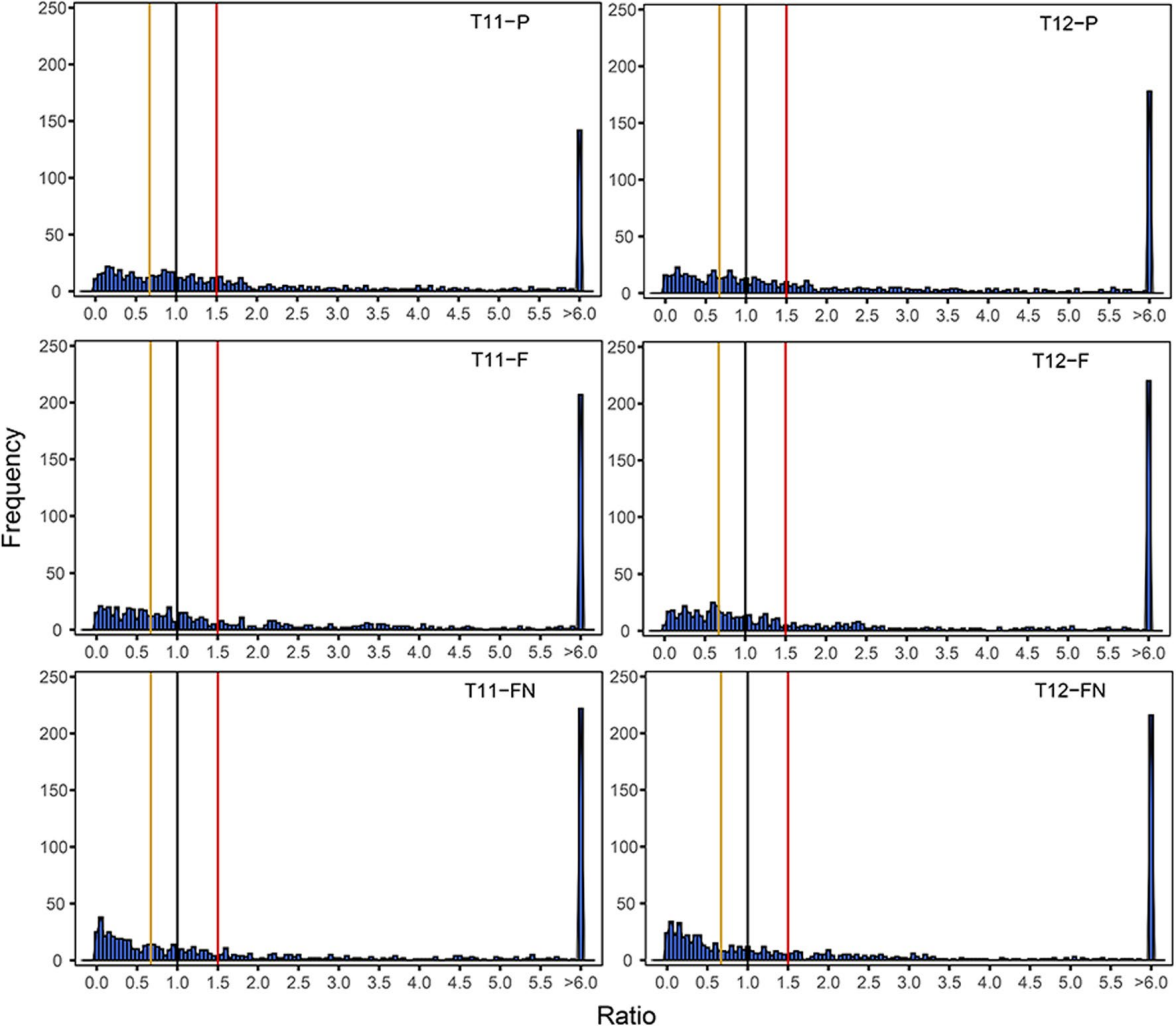
Ratio distributions of gene expression of the functional class of stress-related genes in T11 and T12. The ratios were plotted with bins of 0.05 increments. X-axis represents the ratio bin of fold-change in gene expression levels and Y-axis represents the gene frequency per bin

**Fig. 6:**
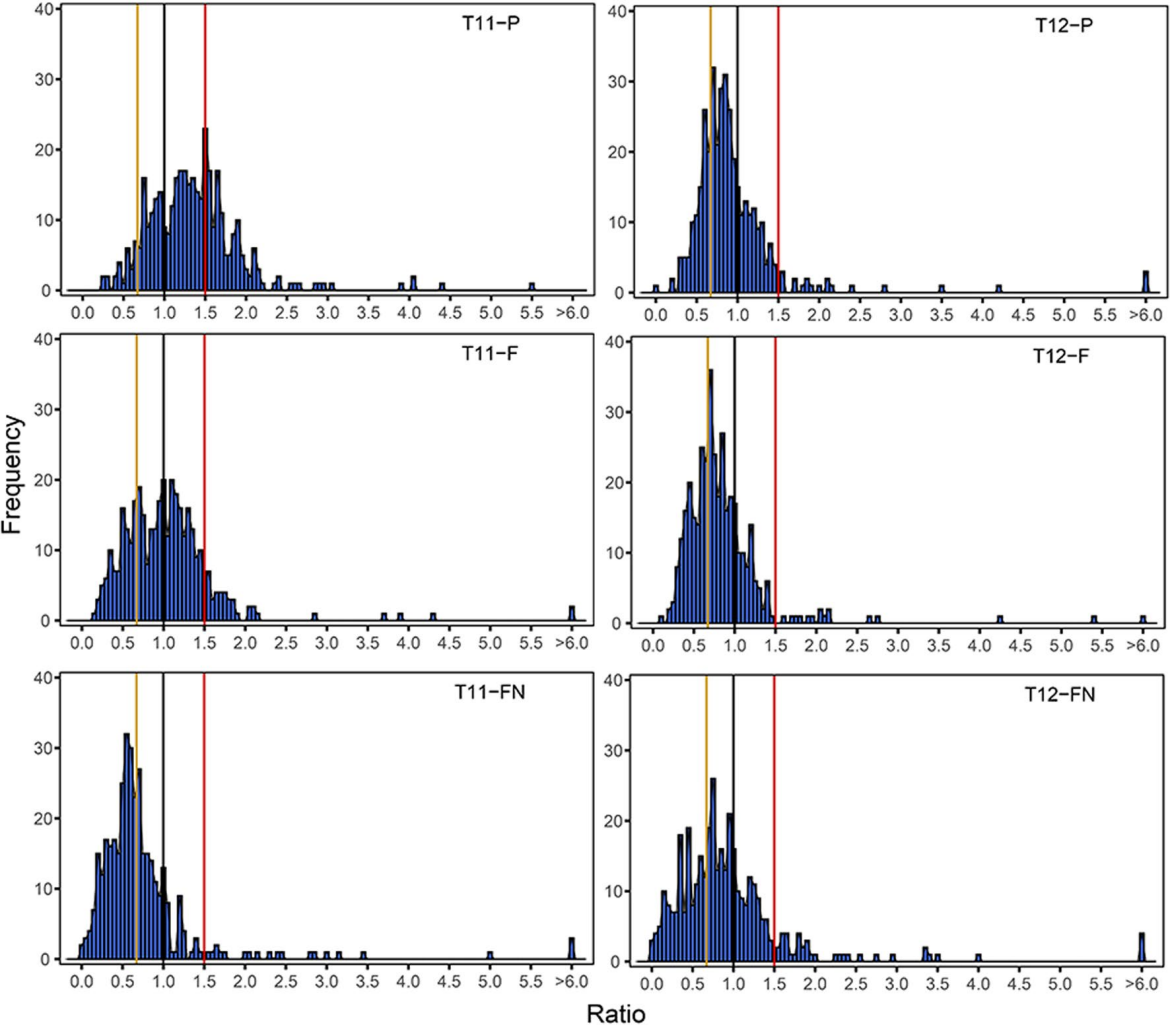
Ratio distributions of gene expression of for the functional class of ribosomal structural genes in T11 and T12. The ratios were plotted with bins of 0.05 increments. X-axis represents the ratio bin of fold-change in gene expression levels and Y-axis represents the gene frequency per bin

Genes were divided into eight functional groups and plotted the ratio distributions to determine whether there is any specific response to aneuploid and diploid (Johnson et al 2020). Genes in each functional group are assembled from various types of resources, including web databases, published article resources, Kyoto Encyclopedia of Genes and Genomes (KEGG) and GO terms (Additional file 6: Table S5). Genes related to stress were performed to analyze whether the occurrence of aneuploidy might induce the response of genes in that category. The distribution of gene expression ratios shows that the number of genes with gene expression ratios between 1.00–1.50 is quite few, but there are abundant genes with gene expression ratio > 1.50 and gene expression ratio < 0.67. The above results indicating that exception of a few genes, the vast majority of stress-related genes are commonly induced to higher or lower extremes of expression in these types of aneuploids (Fig. 5). Another characteristic group is that the ribosomal structural genes, this class of genes exhibit diametrically opposed gene distribution ratio in both T11 and 12 (Fig. 6). T11 exhibits a positive response (1.00–1.50), but near the negative response (0.67–1.00) in T12. The imbalance of ribosomal proteins has the potential to lead to phenotypic effects (Casanova-Saez et al 2014). Nevertheless, this imbalance may not only be caused only by dosage effects in rice aneuploids. Genes encoding peroxisome proteins exhibits a gene expression ratio distribution with a similar pattern to the ribosome, the major peak near the positive effect (1.00–1.50) in T12-P and T12-F, but with a ratio of 1.00 being defined as no modulation both in T11-P and T11-F (Additional file 1: Fig. S9).

Chloroplast and mitochondria are subcellular bioenergetic organelles with their own genomes and genetic systems, each contain a small genome that relies largely on nuclear factors for maintenance and expression (Newton et al 2009; Bennetzen and Hake 2009). However, when the balance was disturbed, genes encoding proteins targeted to the chloroplasts were only affected in T11-P (1.00–1.50) (Additional file 1: Fig. S10). The distribution of mitochondria-targeted genes has no significant fluctuations (0.67–1.00) in all types of aneuploids (Additional file 1: Fig. S11). Proteasomal-, signaling- and transcription factor-targeted genes showed the similar distribution, the gene expression ratio is around 1.00 in parental primary trisomy, but the main peak is gradually shifted to the left in the filial (Additional file 1: Fig. S12, S13 and S14), suggesting that gene expression level of genome functional groups was affected over generation of aneuploid.

## Discussion

Early studies on polyploid and aneuploid have shown that the changes in aneuploidy can produce more severe phenotypic consequences than ploidy. Unbalanced chromosome numbers (aneuploid) have a profound effect on the growth and development of eukaryotic organisms which can trigger a range of changes in transcription; proteotoxic stress; genomic instability and response to interferons. These can ultimately lead to distinct phenotypic features (McClintock 1929; Singh et al 1996; Henry et al 2010). The degree of genomic imbalance can be answered by the ratio of regulation of *cis* and *trans* gene expression (Blakeslee et al 1920). In our study, both *cis* and *trans* gene expression ratio in parental and filial aneuploid were examined, the parental aneuploid preferring intermediate, and filial aneuploid manifested as a typical dosage compensation. The impact of gene dosage effects appears to be attenuated during transmission from parental to its offspring and the phenotypes of filial seems to be recovered. For *trans* genes, their prevalence has been reported in aneuploid of many species (Makarevitch et al 2008; Guo and Birchler 1994; Huettel et al 2008), the changes in *trans* genes in expression ratios appear to be *cis*-related. When the *cis* gene expression ratio approaches 1.00, the broad peak of the *trans* gene shifts further to the left, illustrating there is a relationship between the *cis* and *trans* genes. A global genome-wide cascading modulations in aneuploid was determined, potentially there is molecular basis common to aneuploid in multiple species (Birchler 1981). Different types of aneuploids show more similar expression patterns, the effect of aneuploidy on gene expression imbalance may be caused mainly by imbalance in the chromosome itself rather than dominated by gene-specific dosage effect, viz chromosomally distinct aneuploidies produce highly similar global expression profiles. This is consistent with previous findings in humans pluripotent stem cells and wheat (Ben-David et al 2014; Zhang et al 2017).

The abnormalities in chromosome number are capable of disrupting gene expression balance, thereby reducing fitness and viability in organisms (Wheeler et al 2016). When the products of dosage-sensitive genes are disrupted, global alterations can be observed (Henry et al 2006). In all DEGs, we defined dosage-sensitive genes with gene expression fold-change ≥ 1.50 and *p*-value ≤ 0.05. Venn diagrams shows that dosage-sensitive genes not only exist in trisomic, but also in T11- and T12-FN. In addition to this, the gene expression ratio of T11- and T12-FN deviates from the theoretical concept and the gene expression pattern were more similar to aneuploids than diploid, indicating that the effect of aneuploidy may shift dynamically in its dosage as it is transferred from parental to filial, but its effects are far-reaching and cannot be easily faded away. According to the analyses, it is not difficult to find that the expression patterns of the T11- and T12-FN exhibit a large proportion of upregulated genes. However, in contrast to aneuploidy, there are also a large proportion of downregulated genes in T11- and T12-FN. Dosage compensation mechanisms have been reported in different organisms, which significantly attenuate the adverse effects of dosage imbalance (Birchler and Veitia 2012; Sun et al 2013a; Veitia and Potier 2015).

A meta-analysis on gene expression data form aneuploid cells in diverse organisms, identified different types of aneuploids in different organisms affect similar cellular pathways (Sheltzer et al 2012). We found a large proportion of upregulated overlap genes in two types of primary trisomy, and the GO terms were approximately the same between those two, but for downregulated overlapped genes, the pattern is the exact opposite. Based on the above phenomena, we conclude that in different types of aneuploid in rice, there is commonality in the co-upregulated genes and individuality in the co-downregulated genes. This may be due to the fact that upregulated genes are mainly regulated by the imbalance genome, while downregulated genes are mainly influenced by the added chromosomes. It has been shown that primary trisomy in rice have a single-addition chromosome-specific phenotype which are correlated with defective cell proliferation and growth retardation (Khush et al 1984), similar studies exist in mammals and yeast (Donnelly et al 2014; Torres et al 2007; Williams et al 2008). In our study, the upregulated genes of rice aneuploid were mainly involved in biological processes, signal transduction and transporter activity, it is the addition of chromosome by having aneuploidy that increases the cellular demand for energy, demonstrating the reduced efficiency of aneuploid cells in converting nutrients into biomass (Torres et al 2007; Williams et al 2008). The presence of iron-sulfur cluster and ribosome-associated GO terms in downregulated genes in rice aneuploid that affects the proliferative capacity of aneuploid cells, and studies on breast cancer cell lines showed that iron-sulfur cluster deficiency was strongly activate the iron-starvation, induced ferroptosis (Terzi et al 2021). These GO terms that are significantly involved in either up- or down-regulated genes are associated with growth and developmental defects that occur in aneuploid.

Research of genes in aneuploid and haploid genomes has revealed a stoichiometric equilibrium between them, and when this equilibrium is disrupted, the regulatory genes of signal transduction and transcription factors preferentially exhibit dosage-sensitive (Birchler and Veitia 2007; Blanc and Wolfe 2004; Chapman et al 2006). The genes were divided into eight different functional groups to determine whether rice aneuploid would have a specific response to a certain class of genes. For the moment, no summarized pattern was found for the expression ratios of these two functional groups in four types aneuploid in rice. Stress-related genes may be induced within the aneuploid genome (Ohashi et al 2015) and the genes that were involved in the response to stress in aneuploids were consistently upregulated (Sheltzer et al 2012). In this study, the gene expression of stress-related genes was induced to higher or lower extremes. Upregulated genes are mainly related with various types of response to stress while the downregulated genes are mainly related with various binding of chemical compound. Genes in some different functional classes exhibit responses to aneuploidy, with ribosomal, peroxisome, nuclear chloroplast and mitochondrial genes.

Epigenetic modifications are defined as those affecting phenotypic outcomes (Desai et al 2015). Current research on epigenetic modifications associated with aneuploid has mainly focused on DNA methylation. In human, the level of genome-wide DNA methylation of trisomic 8 and 21 has a tendency toward increased (Davidsson et al 2013; Kerkel et al 2010). In plants, the variation of DNA methylation in aneuploid genome changes among species. Only trisomy 4 showed the most significant change with increased CHH methylation in gene body, while the other types of trisomes did not show generalized effect on DNA methylation in *Arabidopsis thaliana* (Hou et al 2018). However, extensive changes in wheat genome-wide cytosine DNA methylation patterns occured in a set of aneuploids associated with chromosomes 1A, 1B and 1D. Furthermore, aneuploidy-induced DNA methylation alterations lead to inheritable epigenetic difference in wheat (Gao et al 2016). In this study, there is no change in the number of genes in aneuploid genome, but parental and filial trisomic plants have significant changes in multiple phenotypic traits. Even though the phenotypic traits of T11- and T12-FN were analogous to those of wild type, but there were still aneuploid dosage-sensitive genes within the genome. We speculate that there is heritable epigenetic diversification in rice, and the mechanism of epigenetic regulation needs further study in the future.

## Conclusion

Studies on rice aneuploid have been sparsely reported, and whether aneuploidy effects vary with the transmission of generations has not been explained. Our results clearly demonstrate that the interaction between *cis* and *trans* genes in rice aneuploids. The gene expression patterns of rice aneuploid are not related to the added chromosomes but its affected by the imbalance genome. Compared with parental primary trisomy, dosage-sensitive genes were reduced in filial, while dosage-sensitive genes were still present in the filial of normal plants in aneuploid progenies, suggesting that the effects of aneuploidy are profound and cannot be easily eliminated. Our study provides an important basis for a deeper understanding of the regulatory and functional interactions of genes within the rice aneuploid genome and their phenotypic manifestation, gene regulation and functional interactions as well as the transmission characteristics in the rice aneuploid genome.

## Supplementary Information

**Additional file 1**. **Figure S1**. Oligo-painting FISH identification of rice primary trisomy and diploid on mitotic metaphase chromosomes. **Figure S2**. Phenotypic traits between aneuploid and diploid in different generations of rice during the maturity period. **Figure S3**. RT-qPCR and mRNA-seq compared the expression levels of different types of genes. **Figure S4**. Frequency distribution of genes with different expression levels in T12 (T12-P, T12-F and T12- FN). **Figure S5**. GO enrichment analysis of differentially expressed *cis* and *trans* genes. **Figure S6**. GO enrichment analysis of genes with no change in aneuploids. **Figure S7**. GO enrichment analysis of up- and down-regulated DEGs in primary trisomy and diploid from aneuploid offspring. **Figure S8**. GO enrichment analysis of T11-FN and T12-FN. **Figure S9**. Ratio distributions of expression of genes for the functional class of peroxisomal genes in T11 and T12. **Figure S10**. Ratio distributions of expression of genes for the functional class of nuclear chloroplast genes in T11 and T12. **Figure S11**. Ratio distributions of expression of genes for the functional class of nuclear mitochondrial genes in T11 and T12. **Figure S12**. Ratio distributions of expression of genes for the functional class of proteasomal genes in T11 and T12. **Figure S13**. Ratio distributions of expression of genes for the functional class of signaling genes in T11 and T12. **Figure S14**. Ratio distributions of expression of genes for the functional class of transcription factors (TFs) genes in T11 and T12.

**Additional file 2.** **Table S1**. mRNA-seq alignment summary.

**Additional file 3.** **Table S2**. Primer sequence of RT-qPCR genes.

**Additional file 4.** **Table S3**. The ratio of different expression level DEGs in express genes.

**Additional file 5.** **Table S4**. The ratio of up- and down-regulated genes in different expression level DEGs.

**Additional file 6.** **Table S5**. Gene lists of diverse functional groups used in this study.

## Data Availability

All original sequencing data for this work are available in NCBI SRA database with accession number PRJNA909164.

## Abbreviations

GO: Gene Ontology
EGs: Expressed genes
KEGG: Kyoto encyclopedia of genes and genomes
mRNA-seq: mRNA-sequence
T11/T12-P: Parental primary trisomy 11/12
T11/T12-F: Filial primary trisomy 11/12
FPKM: Fragments per kilobase million
FDR: False discovery rate
DEGs: Differentially expressed genes
Oligo-FISH: Oligonucleotide-fluorescence in situ hybridization
RT-qPCR: Real time quantitative PCR
