# Supplementary material for: Genome-Wide Effects on Gene Expression Between Parental and Filial Generations of Trisomy 11 and 12 of Rice
Source: Rice (N Y). 2023 Mar 25;16:17. doi: 10.1186/s12284-023-00632-5 (PMC10039966; doi:10.1186/s12284-023-00632-5)
Supplement: Supplementary file 1 — Additional file 1. Figure S1. Oligo-painting FISH identification of rice primary trisomy and diploid on mitotic metaphase chromosomes. Figure S2. Phenotypic traits between aneuploid and diploid in different generations of rice during the maturity period. Figure S3. RT-qPCR and mRNA-seq compared the expression levels of different types of genes. Figure S4. Frequency distribution of genes with different expression levels in T12 (T12-P, T12-F and T12- FN). Figure S5. GO enrichment analysis of differentially expressed cis and trans genes. Figure S6. GO enrichment analysis of genes with no change in aneuploids. Figure S7. GO enrichment analysis of up- and down-regulated DEGs in primary trisomy and diploid from aneuploid offspring. Figure S8. GO enrichment analysis of T11-FN and T12-FN. Figure S9. Ratio distributions of expression of genes for the functional class of peroxisomal genes in T11 and T12. Figure S10. Ratio distributions of expression of genes for the functional class of nuclear chloroplast genes in T11 and T12. Figure S11. Ratio distributions of expression of genes for the functional class of nuclear mitochondrial genes in T11 and T12. Figure S12. Ratio distributions of expression of genes for the functional class of proteasomal genes in T11 and T12. Figure S13. Ratio distributions of expression of genes for the functional class of signaling genes in T11 and T12. Figure S14. Ratio distributions of expression of genes for the functional class of transcription factors (TFs) genes in T11 and T12. [file 12284_2023_632_MOESM1_ESM.docx]

**Genome-wide Effects on Gene Expression Between Parental and Filial Generations of Trisomy 11 and 12 of Rice**

# Shang Sun^1†^, Kai Liu^1†^, Chao Xue^2^, Yingying Hu^2^, Hengxiu Yu^1^, Guoxiao Qi^1^, Jijin Chen^1^, Xiya Li^2^, Xinru Zhao^2^, Zhiyun Gong^1, 2*^

**Supplementary figure legends**

**Fig. S1** Oligo-painting FISH identification of rice primary trisomy and diploid on mitotic metaphase chromosomes.

**Fig. S2** Phenotypic traits between aneuploid and diploid in different generations of rice during the maturity period.

**Fig. S3** RT-qPCR and mRNA-seq compared the expression levels of different types of genes.

**Fig. S4** Frequency distribution of genes with different expression levels in T12 (T12-P, T12-F and T12- FN).

**Fig. S5** GO enrichment analysis of differentially expressed *cis* and *trans* genes.

**Fig. S6** GO enrichment analysis of genes with no change in aneuploids.

**Fig. S7** GO enrichment analysis of up- and down-regulated DEGs in primary trisomy and diploid from aneuploid offspring.

**Fig. S8** GO enrichment analysis of T11-FN and T12-FN.

**Fig. S9** Ratio distributions of expression of genes for the functional class of peroxisomal genes in T11 and T12.

**Fig. S10** Ratio distributions of expression of genes for the functional class of nuclear chloroplast genes in T11 and T12.

**Fig. S11** Ratio distributions of expression of genes for the functional class of nuclear mitochondrial genes in T11 and T12.

**Fig. S12** Ratio distributions of expression of genes for the functional class of proteasomal genes in T11 and T12.

**Fig. S13** Ratio distributions of expression of genes for the functional class of signaling genes in T11 and T12.

**Fig. S14** Ratio distributions of expression of genes for the functional class of transcription factors (TFs) genes in T11 and T12.

Shang Sun and Kai Liu have contributed equally to this work.

Correspondence: zygong@yzu.edu.cn

^1^ Jiangsu Key Laboratory of Crop Genomics and Molecular Breeding/Key Laboratory of Plant Functional Genomics of the Ministry of Education/Jiangsu Key Laboratory of Crop Genetics and Physiology, Agricultural College of Yangzhou University, Yangzhou, 225009, China

^2^ Jiangsu Co-Innovation Center for Modern Production Technology of Grain Crops, Yangzhou University, Yangzhou, 225009, China


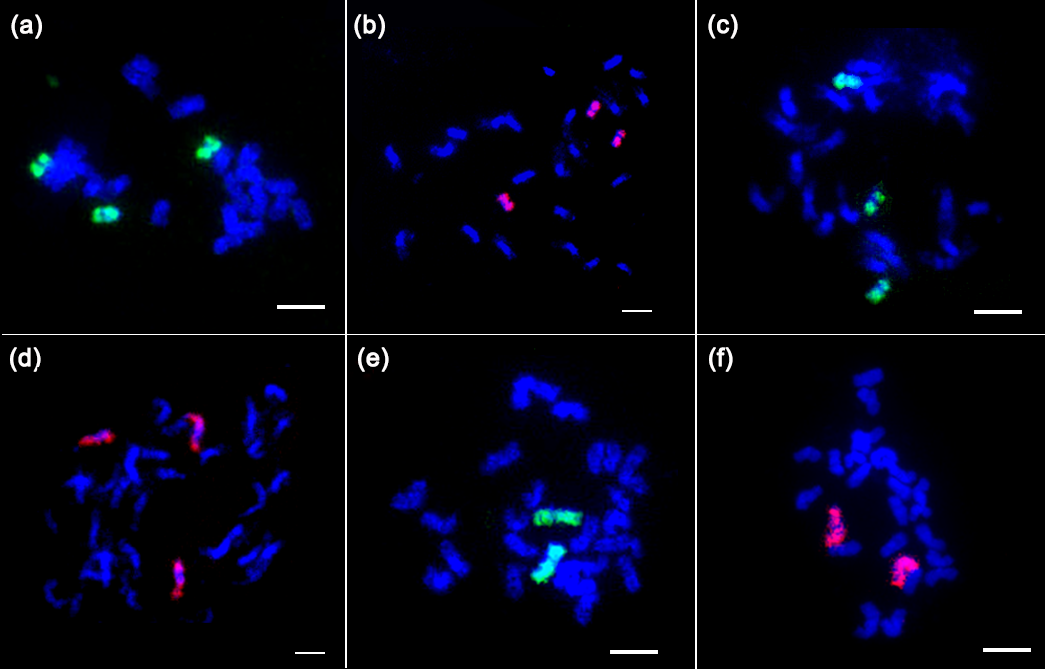


**Fig. S1** Oligo-painting FISH identification of rice primary trisomy and diploid on mitotic metaphase chromosomes.

**a** Oligo-painting FISH identification of parental primary trisomy 11 on mitotic metaphase chromosomes using FAM-green labeled whole chromosome 11 painting probes. **b** Oligo-painting FISH identification of parental primary trisomy 12 on mitotic metaphase chromosomes using digoxigenin-red labeled whole chromosome 12 painting probes. **c** and **d** Filial parental primary trisomy 11 and 12, respectively. **e** and **f** T11-FN and T12-FN, respectively. Chromosomes were counterstained with 4’,6-diamidino-2-phenylindole. Bars = 5 μm.


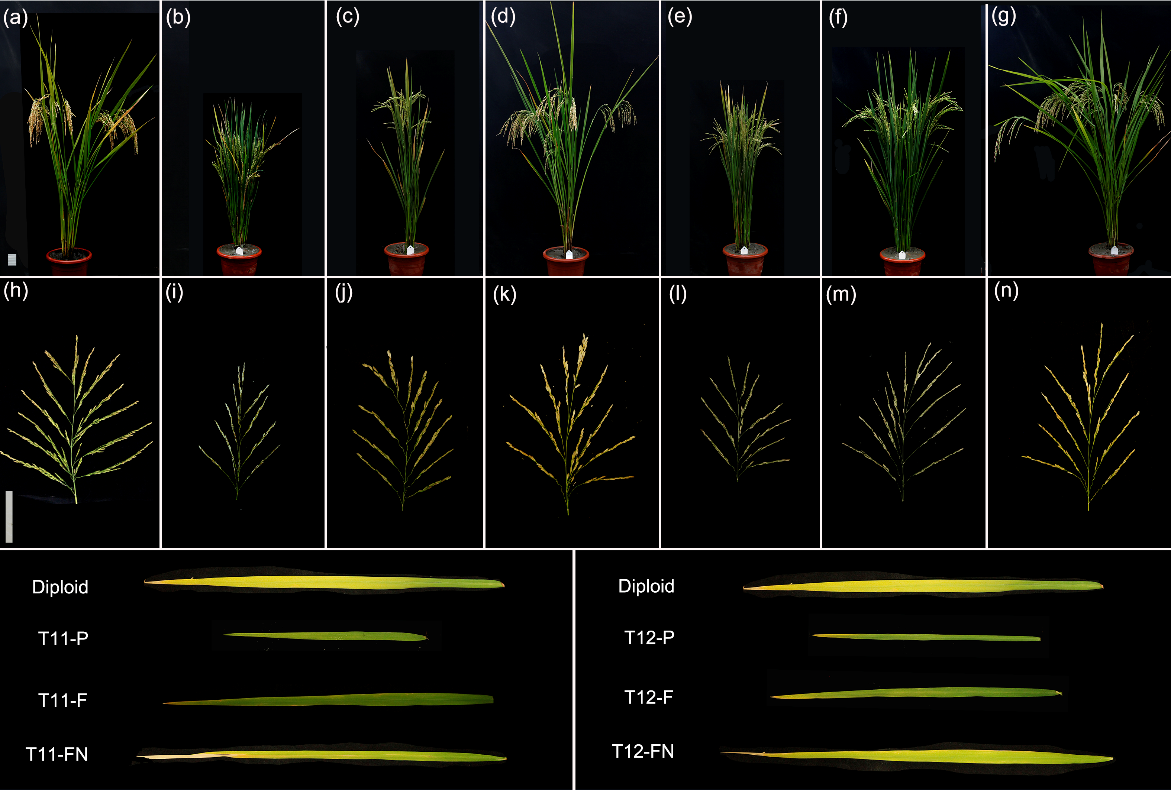


**Fig. S2** Phenotypic traits between aneuploid and diploid in different generations of rice during maturity period.

**a** Zhongxian 3037. **b**, **c** and **d** T11-P, T11-F and T11-FN, respectively. **e**, **f** and **g** T12-P, T12-F and T12-FN, respectively. Bars = 6 cm. **h**-**n** were corresponding spike features. Bars = 7 cm.


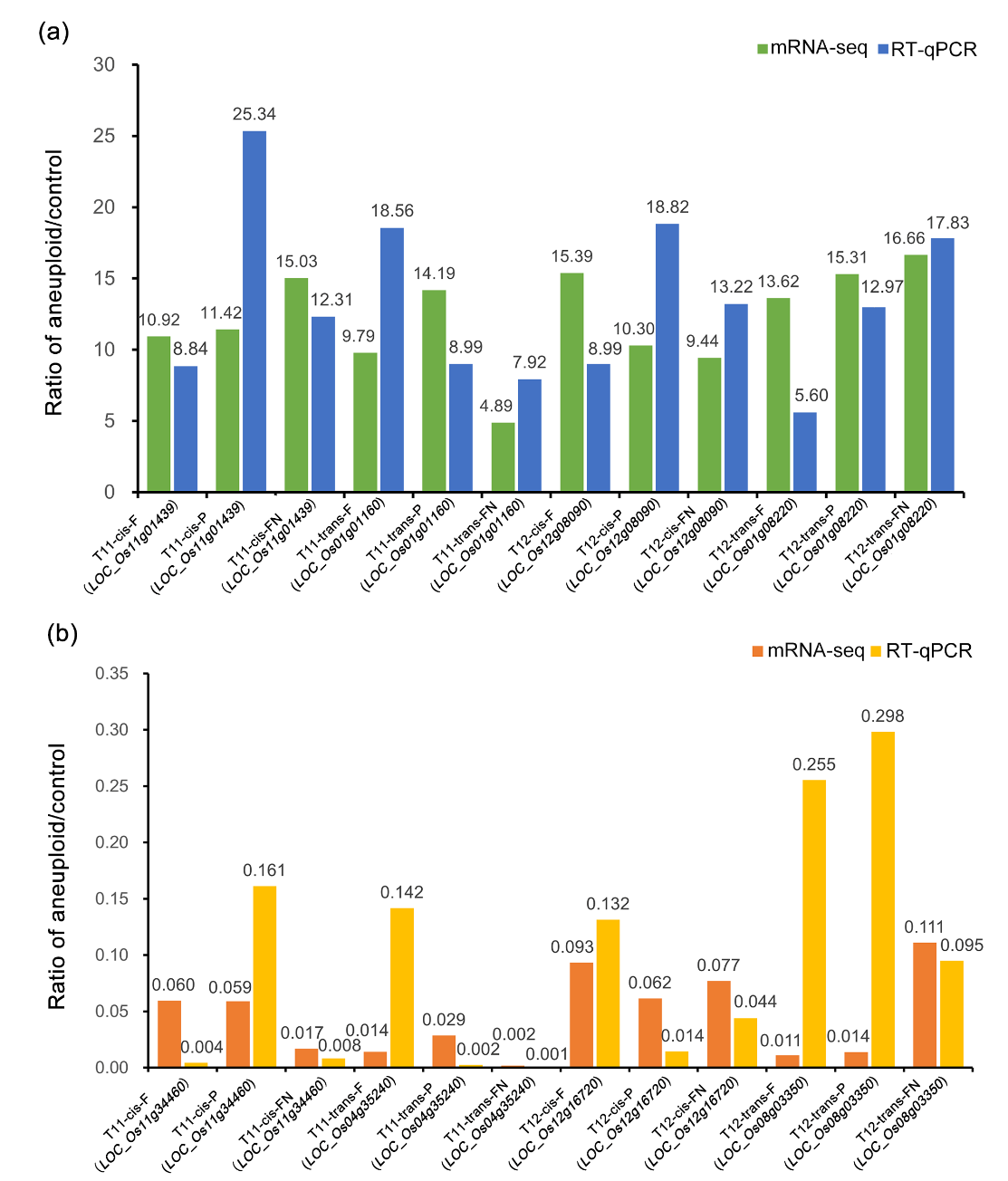


**Fig. S3** RT-qPCR and mRNA-seq compared the expression levels of different types of genes.

**a** The upregulated genes. **b** The downregulated genes. The ratio of aneuploid/control > 1.50 represent the genes were upregulated, the ratio of aneuploid/control < 0.67 represent the genes were downregulated.


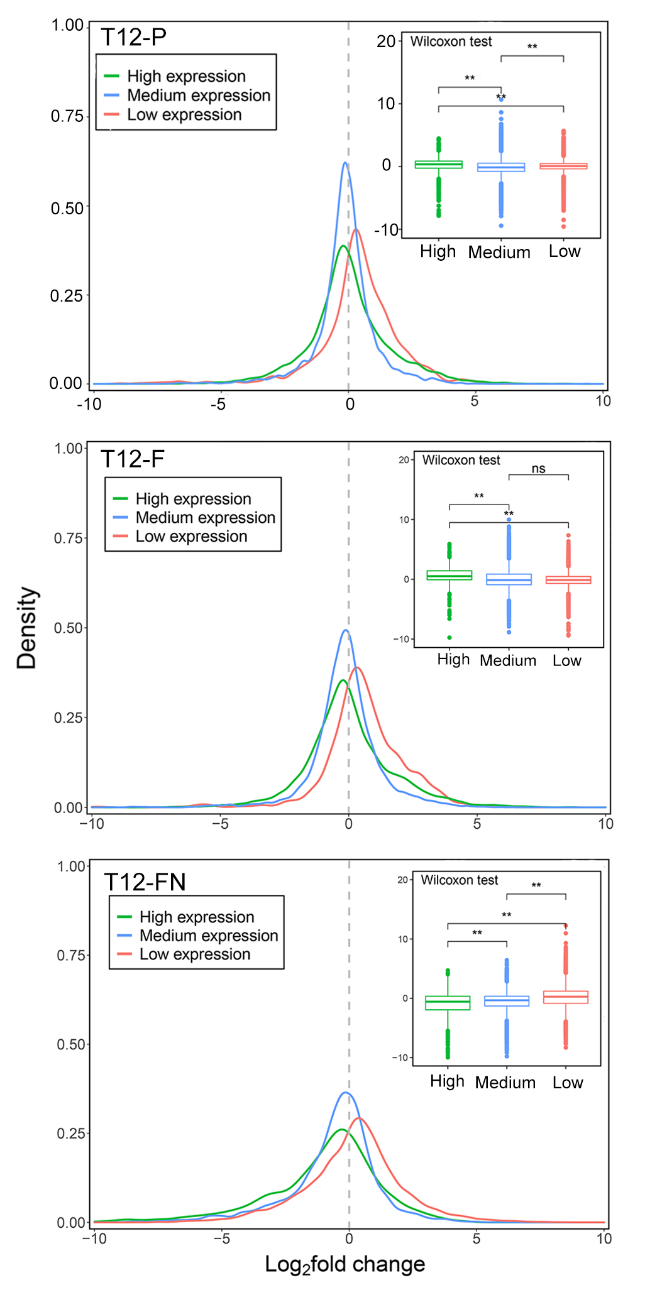


**Fig. S4** Frequency distribution of genes with different expression levels in T12 (T12-P, T12-F and T12- FN).

X-axis represents the Log_2_(fold change) in gene expression levels between diploid and trisomy and Y-axis represents frequency distributions of Log_2_(fold change). The red, blue and green lines indicate low (0 < FPKM ≤ 10), medium (10 < FPKM ≤ 100) and high (FPKM > 100) expression genes, respectively. Boxplot on the right is a statistical analysis of the expression levels of the different types of genes (Wilcoxon test, ** *p-*value < 0.01; ns, not significant).


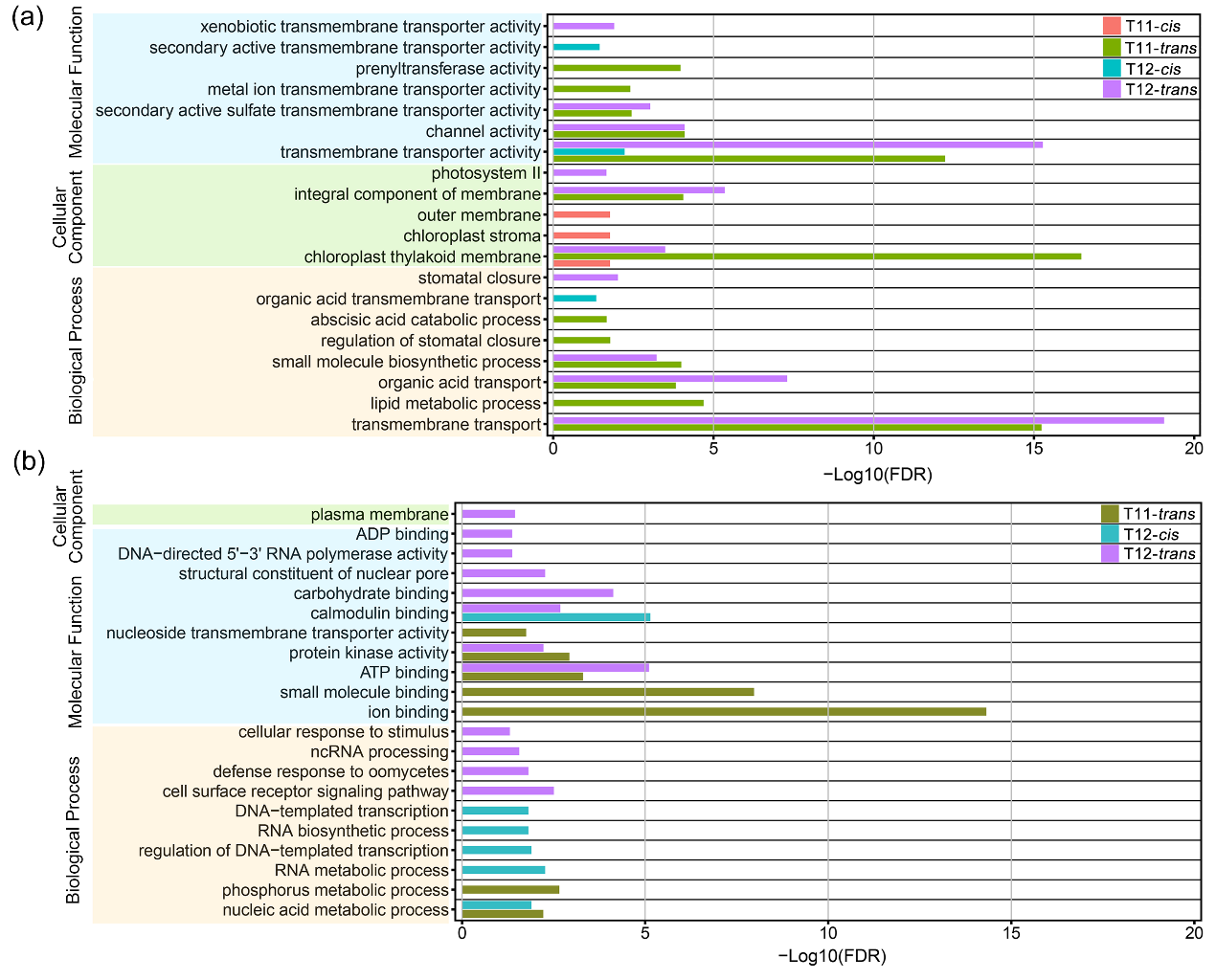


**Fig. S5** GO enrichment analysis of differentially expressed *cis* and *trans* genes.

**a** GO enrichment analysis of 1,089 and 1,292 upregulated overlapped genes in T11 (overlapped T11-P and T11-F) and T12 (overlapped T12-P and T12-F), respectively. **b** GO enrichment analysis of 540 and 789 downregulated overlapped genes in T11 (overlapped T11-P and T11-F) and T12 (overlapped T12-P and T12-F), respectively. The statistics of the number of DEGs are from Figure 4.


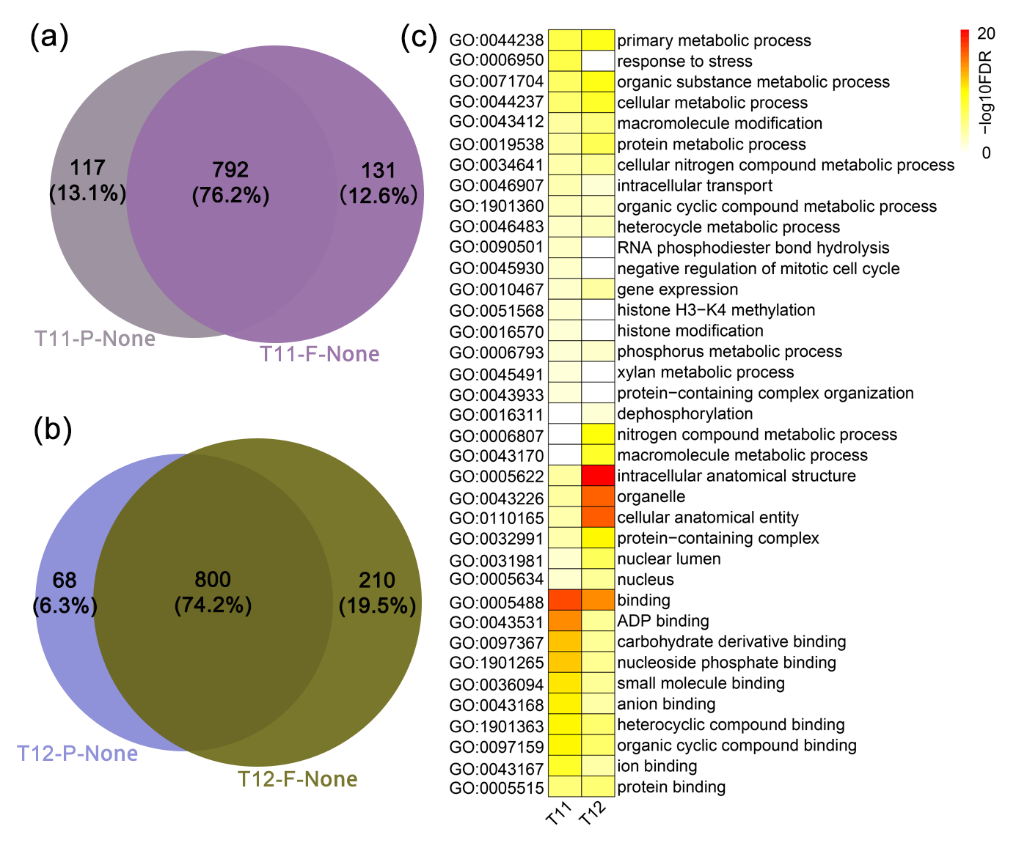


**Fig. S6** GO enrichment analysis of genes with no change in aneuploids.

**a** and **b** Venn diagrams overlapped the *cis* genes with no change in T11 (T11-P and T11-F) and T12 (T12-P and T12-F), respectively. **c** GO enrichment analysis of the *cis* genes with no change in T11 (T11-P and T11-F) and T12 (T12-P and T12-F), respectively.


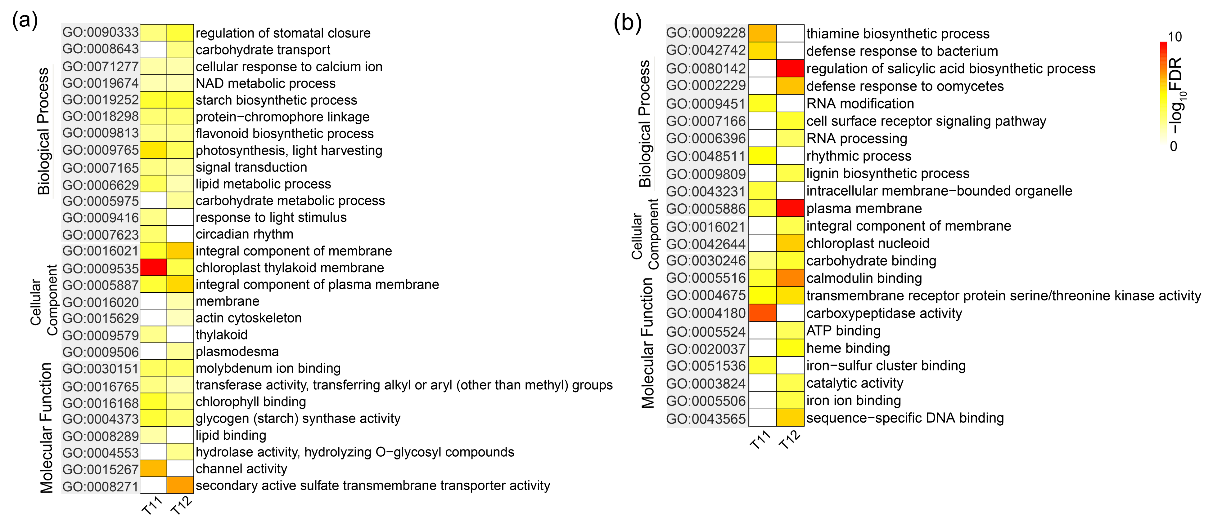


**Fig. S7** GO enrichment analysis of up- and down-regulated DEGs in primary trisomy and diploid from aneuploid offspring.

**a** Heatmap of GO enrichment analysis of upregulated genes in T11 (overlapped the T11-P, T11-F and T11-FN) and T12 (overlapped the T12-P, T12-F and T12-FN). **b** Heatmap of GO enrichment analysis of downregulated genes in T11 (overlapped the T11-P, T11-F and T11-FN) and T12 (overlapped the T12-P, T12-F and T12-FN).


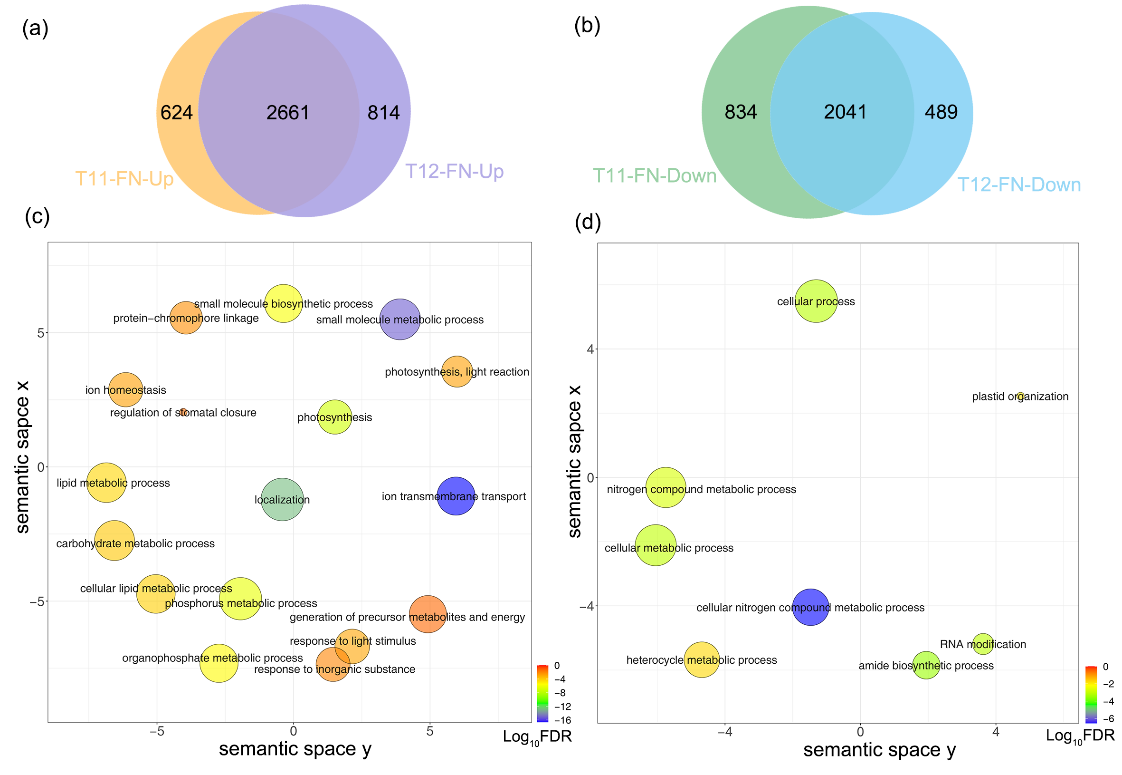


**Fig. S8** GO enrichment analysis of T11-FN and T12-FN.

**a** and **b** Venn diagrams of the up- and down-regulated genes in T11-FN and T12-FN, respectively. **c** and **d** GO enrichment analysis of the up- and down-regulated genes in T11-FN and T12-FN, respectively.


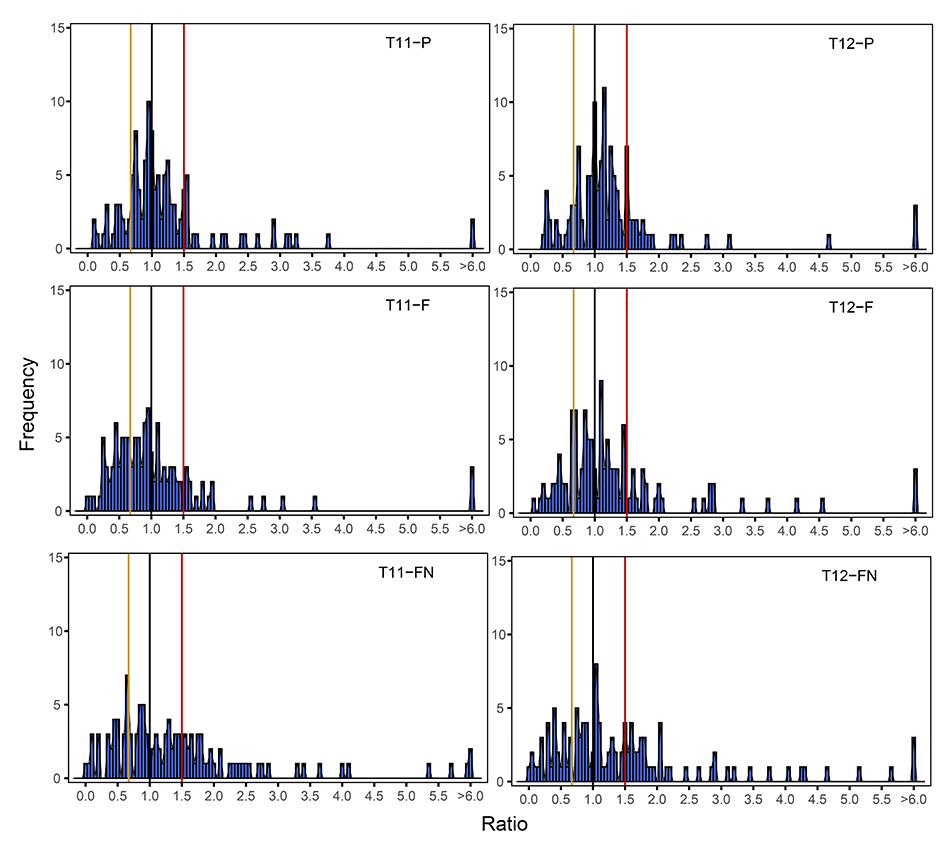


**Fig. S9** Ratio distributions of expression of genes for the functional class of peroxisomal genes in T11 and T12. The ratios were plotted with bins of 0.05 increments. X-axis represents the ratio bin of fold-change in gene expression levels and Y-axis represents the gene frequency per bin.


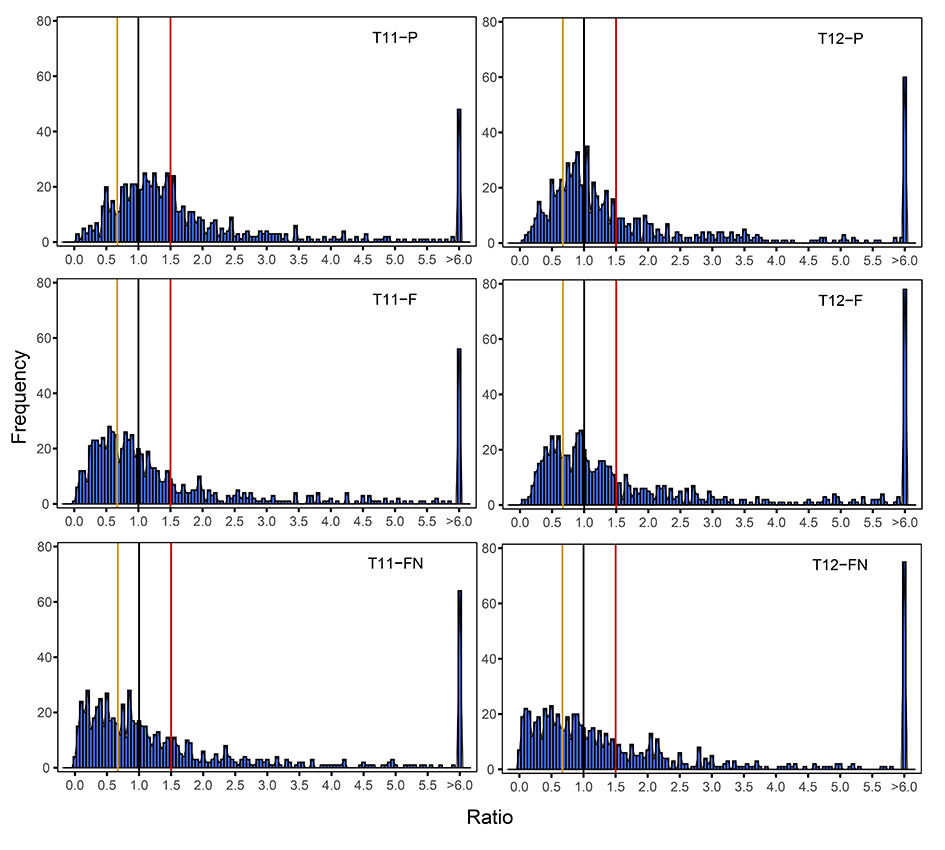


**Fig. S10** Ratio distributions of expression of genes for the functional class of nuclear chloroplast genes in T11 and T12. The ratios were plotted with bins of 0.05 increments. X-axis represents the ratio bin of fold-change in gene expression levels and Y-axis represents the gene frequency per bin.


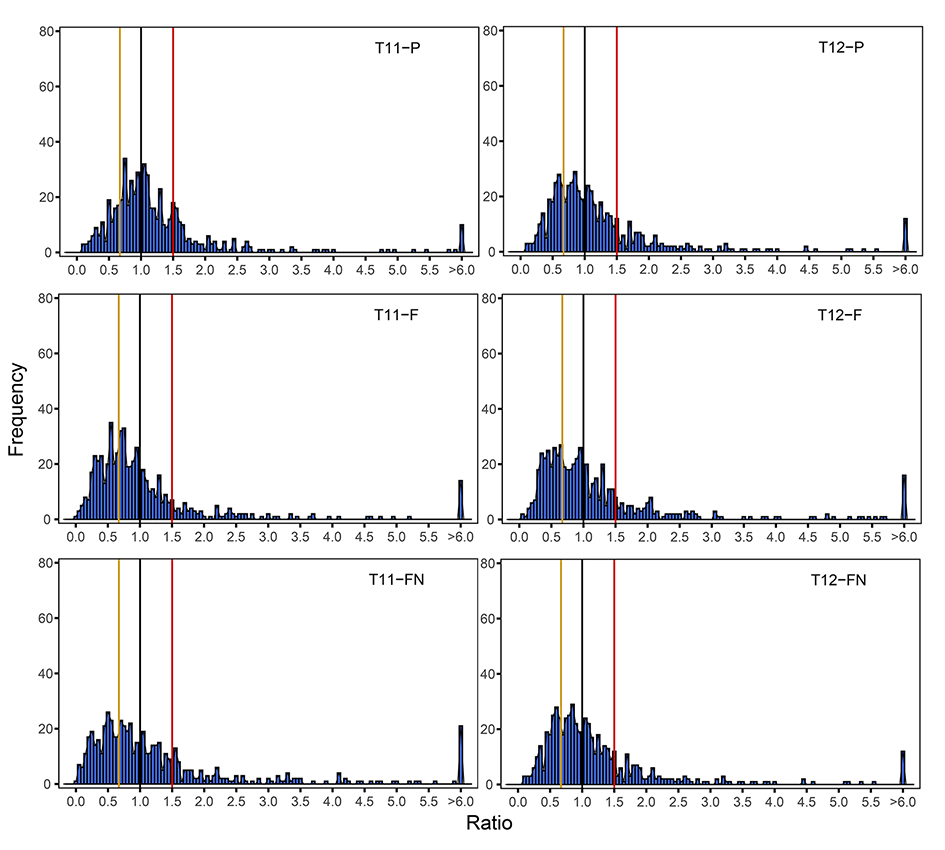


**Fig. S11** Ratio distributions of expression of genes for the functional class of nuclear mitochondrial genes in T11 and T12. The ratios were plotted with bins of 0.05 increments. X-axis represents the ratio bin of fold-change in gene expression levels and Y-axis represents the gene frequency per bin.


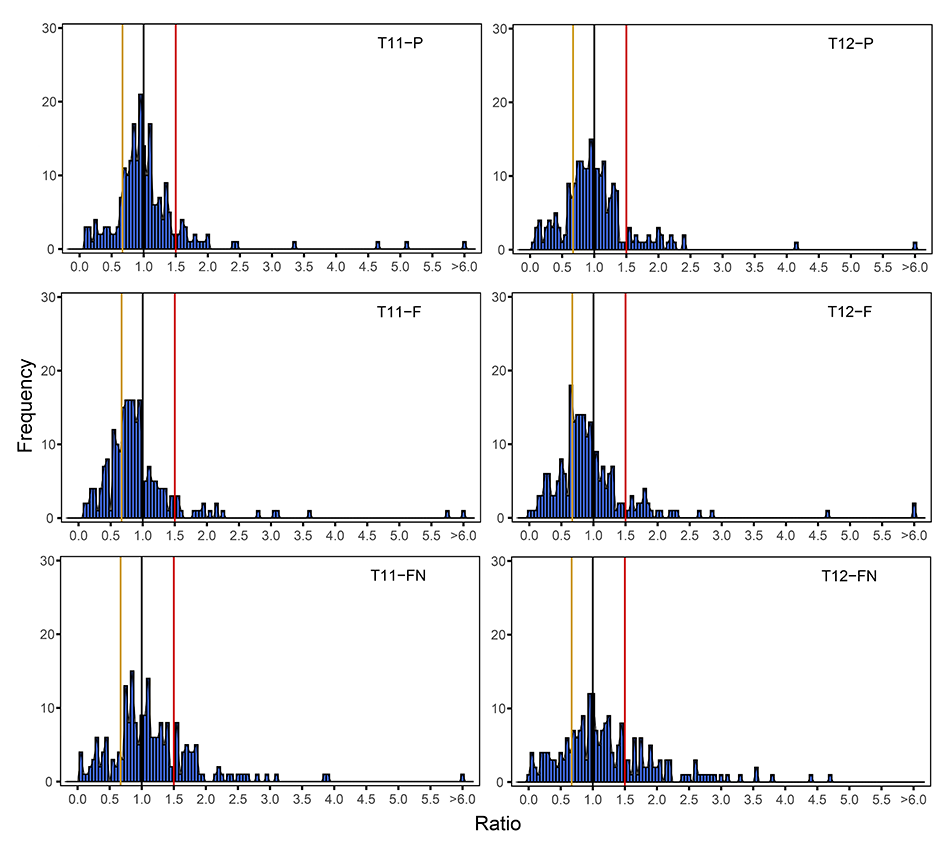


**Fig. S12** Ratio distributions of expression of genes for the functional class of proteasomal genes in T11 and T12. The ratios were plotted with bins of 0.05 increments. X-axis represents the ratio bin of fold-change in gene expression levels and Y-axis represents the gene frequency per bin.


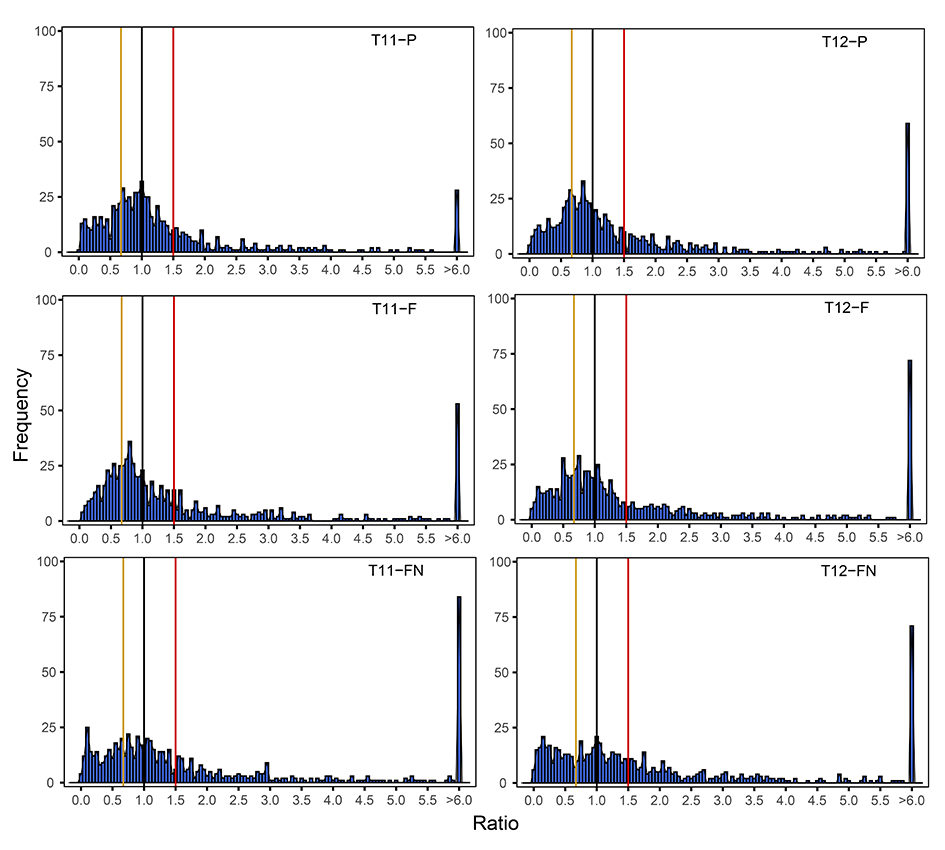


**Fig. S13** Ratio distributions of expression of genes for the functional class of signaling genes in T11 and T12. The ratios were plotted with bins of 0.05 increments. X-axis represents the ratio bin of fold-change in gene expression levels and Y-axis represents the gene frequency per bin.


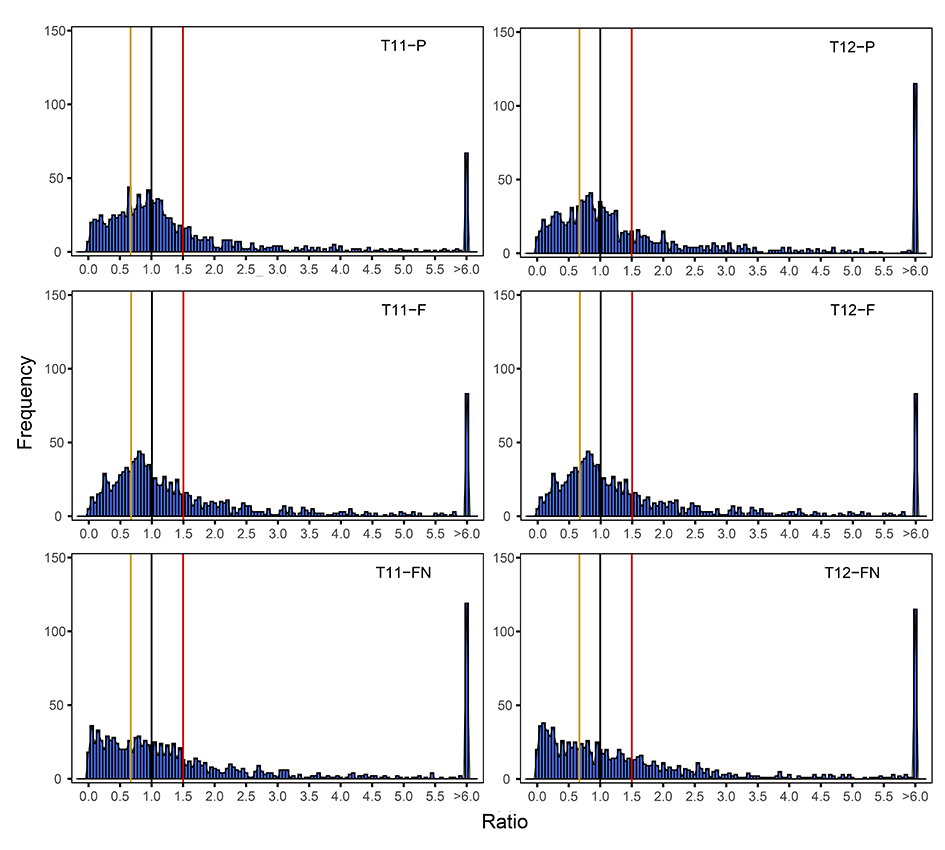


**Fig. S14** Ratio distributions of expression of genes for the functional class of transcription factors (TFs) genes in T11 and T12. The ratios were plotted with bins of 0.05 increments. X-axis represents the ratio bin of fold-change in gene expression levels and Y-axis represents the gene frequency per bin.
