# Supplementary material for: Genome-Wide Effects on Gene Expression Between Parental and Filial Generations of Trisomy 11 and 12 of Rice
Source: Rice (N Y). 2023 Mar 25;16:17. doi: 10.1186/s12284-023-00632-5 (PMC10039966; doi:10.1186/s12284-023-00632-5)
Supplement: Supplementary file 2 — Additional file 2. Table S1. mRNA-seq alignment summary. [file 12284_2023_632_MOESM2_ESM.pdf]

Table S1 mRNA-seq alignment summary

Sample	Replicate	Clean reads	Unique reads	Ratio (%)	Correlation coefficient
Diploid	1	33,209,387	31,391,839	94.53	0.93
Diploid	2	32,900,438	31,771,371	96.57	
T11-P	1	35,516,519	34,280,225	96.52	0.95
T11-P	2	37,880,390	36,306,151	95.84	
T12-P	1	41,449,351	39,751,191	95.90	0.98
T12-P	2	43,148,359	41,513,841	96.21	
T11-F	1	38,785,588	36,660,174	94.52	0.95
T11-F	2	37,228,564	35,919,236	96.48	
T12-F	1	36,643,676	35,377,676	96.55	0.90
T12-F	2	37,156,148	35,946,228	96.74	
T11-FN	1	37,195,062	34,956,461	93.98	0.99
T11-FN	2	39,665,412	36,682,456	92.48	
T12-FN	1	41,802,018	37,193,551	88.98	0.98
T12-FN	2	41,506,657	39,003,437	93.97	
