# Supplementary material for: Genome-Wide Effects on Gene Expression Between Parental and Filial Generations of Trisomy 11 and 12 of Rice
Source: Rice (N Y). 2023 Mar 25;16:17. doi: 10.1186/s12284-023-00632-5 (PMC10039966; doi:10.1186/s12284-023-00632-5)
Supplement: Supplementary file 3 — Additional file 3. Table S2. Primer sequence of RT-qPCR genes. [file 12284_2023_632_MOESM3_ESM.pdf]

Table S2 Primer sequence of RT-qPCR genes

Gene ID	F-primer sequence(5'-3')	R-primer sequence(5'-3')
LOC_Os11g01439	CAGAGATGGCCAAGCAAGGA	TAGGAGGCTCTCGATCTCGG
LOC_Os01g01160	CTGGCTGGAGGAGAACTTGG	GTTCTTCTTCGCATCCGAGC
LOC_Os12g08090	AGACAATCTCCAATGGCGGG	AGGAGTATGCGAAGGCGATG
LOC_Os03g08220	GAAGCCCGAGTCCGTGTGCGCGATG	CTACCTCCTCTTCTGTGACGTG
LOC_Os11g34460	GACGTCACTGTGAGGCTTGA	AGGCGATTACCAACAGCACA
LOC_Os04g35240	GTTCTGTCACTGTCTTTGTAAT	AGATGCTCAATGAGATGGTTTT
LOC_Os12g16720	CCCCCTAACCGACGACAATC	CTAGCTCCGTCATCACCCAC
LOC_Os08g03350	GGACTCCGGCAGATCATCA	CTGGTTTCATCATGTGTGCCTA
