# Supplementary material for: Genome-Wide Effects on Gene Expression Between Parental and Filial Generations of Trisomy 11 and 12 of Rice
Source: Rice (N Y). 2023 Mar 25;16:17. doi: 10.1186/s12284-023-00632-5 (PMC10039966; doi:10.1186/s12284-023-00632-5)
Supplement: Supplementary file 4 — Additional file 4. Table S3. The ratio of different expression level DEGs in express genes. [file 12284_2023_632_MOESM4_ESM.pdf]

Table S3 The ratio of different expression level DEGs in express genes

Comparisons	Low (0<FPKM≤10)			Medium (10<FPKM≤100)			High (FPKM>100)		
	EGs	Total DEGs	Ratio	EGs	Total DEGs	Ratio	EGs	Total DEGs	Ratio
T11-P vs Diploid	11,929	1,411	11.83%	6,857	1,341	19.56%	1,026	274	26.71%
T11-F vs Diploid	12,057	1,572	13.04%	6,819	1,193	17.50%	1,014	218	21.50%
T11-FN vs Diploid	11,731	2,804	23.90%	6,759	2,556	37.82%	996	506	50.80%
T12-P vs Diploid	12,351	2,513	20.35%	6,560	1,720	26.22%	947	313	33.05%
T12-F vs Diploid	12,698	1,584	12.47%	6,243	816	13.07%	846	123	14.54%
T12-FN vs Diploid	11,548	2,723	23.58%	6,582	2,466	37.47%	958	469	48.96%

EGs: Expressed genes
DEGs: Differentially expressed genes
