# Supplementary material for: Genome-Wide Effects on Gene Expression Between Parental and Filial Generations of Trisomy 11 and 12 of Rice
Source: Rice (N Y). 2023 Mar 25;16:17. doi: 10.1186/s12284-023-00632-5 (PMC10039966; doi:10.1186/s12284-023-00632-5)
Supplement: Supplementary file 5 — Additional file 5. Table S4. The ratio of up- and down-regulated genes in different expression level DEGs. [file 12284_2023_632_MOESM5_ESM.pdf]

Table S4 The ratio of up- and down-regulated genes in different expression level DEGs

Comparisons	Low (0<FPKM≤10)					Medium (10<FPKM≤100)					High (FPKM>100)				
	Total DEGs	Up	Up Ratio	Down	Down Ratio	Total DEGs	Up	Up Ratio	Down	Down Ratio	Total DEGs	Up	Up Ratio	Down	Down Ratio
T11-P vs Diploid	1,411	902**	63.93%	509	36.07%	1,341	609	45.41%	732**	54.59%	274	109	39.78%	165**	60.22%
T11-F vs Diploid	1,572	977**	62.15%	595	37.85%	1,193	521	43.67%	672**	56.33%	218	72	33.03%	146**	66.97%
T11-FN vs Diploid	2,804	2,009**	71.65%	795	28.35%	2,556	993	38.85%	1,563**	61.15%	506	109	21.54%	397**	78.46%
T12-P vs Diploid	2,513	1,539**	61.24%	974	38.76%	1,720	780	45.35%	940**	54.65%	313	123	39.30%	190**	60.70%
T12-F vs Diploid	1,584	1,099**	69.38%	485	30.62%	816	360	44.12%	456**	55.88%	123	41	33.33%	82**	66.67%
T12-FN vs Diploid	2,723	2,072**	76.09%	651	23.91%	2,466	1,046	42.42%	1,420**	57.58%	469	101	21.54%	368**	78.46%

EGs: Expressed genes

DEGs: Differentially expressed genes

**The significantly dominant dysregulated group determined by chi-squary (p-value < 0.01)
