# Supplementary material for: Genome-Wide Effects on Gene Expression Between Parental and Filial Generations of Trisomy 11 and 12 of Rice
Source: Rice (N Y). 2023 Mar 25;16:17. doi: 10.1186/s12284-023-00632-5 (PMC10039966; doi:10.1186/s12284-023-00632-5)
Supplement: Supplementary file 6 — Additional file 6. Table S5. Gene lists of diverse functional groups used in this study. [file 12284_2023_632_MOESM6_ESM.pdf]

Table S5 Gene lists of diverse functional groups uesd in this study

transcription factor	signaling	stress	ribosomal	preteasomal	peroxisome	chloroplast	mitochondrial
LOC_Os01g04750	LOC_Os04g52780	LOC_Os01g01420	LOC_Os07g31280	LOC_Os09g37000	LOC_Os02g03070	LOC_Os03g45710	LOC_Os02g52860
LOC_Os01g04800	LOC_Os03g15570	LOC_Os01g02010	LOC_Os10g41520	LOC_Os08g43640	LOC_Os02g44220	LOC_Os08g33650	LOC_Os02g47200
LOC_Os05g47650	LOC_Os03g49640	LOC_Os01g02780	LOC_Os03g55490	LOC_Os09g25310	LOC_Os04g52690	LOC_Os01g31690	LOC_Os06g46450
LOC_Os01g49830	LOC_Os06g05520	LOC_Os01g03330	LOC_Os07g02350	LOC_Os03g63430	LOC_Os02g14790	LOC_Os10g32348	LOC_Os10g21324
LOC_Os01g01290	LOC_Os08g06060	LOC_Os01g03530	LOC_Os03g10940	LOC_Os04g36700	LOC_Os08g39080	LOC_Os08g25890	LOC_Os09g08910
LOC_Os01g01430	LOC_Os04g34050	LOC_Os01g04050	LOC_Os02g49620	LOC_Os04g40850	LOC_Os05g01090	LOC_Os07g04840	LOC_Os12g34124
LOC_Os01g01470	LOC_Os08g01260	LOC_Os01g04090	LOC_Os01g46060	LOC_Os02g38810	LOC_Os01g01720	LOC_Os07g01480	LOC_Os03g20020
LOC_Os01g01600	LOC_Os07g06750	LOC_Os01g04370	LOC_Os03g18840	LOC_Os04g56646	LOC_Os03g57210	LOC_Os07g36080	LOC_Os12g34144
LOC_Os01g01840	LOC_Os02g33600	LOC_Os01g04380	LOC_Os12g43830	LOC_Os01g32800	LOC_Os07g05810	LOC_Os02g36850	LOC_Os09g24404
LOC_Os01g01870	LOC_Os05g27730	LOC_Os01g04580	LOC_Os03g52470	LOC_Os03g11570	LOC_Os10g32960	LOC_Os04g44190	LOC_Os10g21396
LOC_Os01g02110	LOC_Os01g61080	LOC_Os01g04920	LOC_Os07g12320	LOC_Os06g49020	LOC_Os07g41800	LOC_Os08g10020	LOC_Os12g14930
LOC_Os01g03890	LOC_Os05g39720	LOC_Os01g05080	LOC_Os12g41620	LOC_Os05g30800	LOC_Os05g19480	LOC_Os07g05360	LOC_Os10g21418
LOC_Os01g06550	LOC_Os02g54600	LOC_Os01g05840	LOC_Os07g41190	LOC_Os01g16190	LOC_Os02g13270	LOC_Os07g05365	LOC_Os12g34040
LOC_Os01g06640	LOC_Os06g09180	LOC_Os01g06310	LOC_Os06g43690	LOC_Os07g25420	LOC_Os12g03430	LOC_Os04g59440	LOC_Os12g34022
LOC_Os01g06852	LOC_Os06g09190	LOC_Os01g06580	LOC_Os04g50660	LOC_Os01g16640	LOC_Os11g03670	LOC_Os01g64960	LOC_Os12g34018
LOC_Os01g07480	LOC_Os03g17700	LOC_Os01g06740	LOC_Os03g05720	LOC_Os03g13970	LOC_Os08g45210	LOC_Os05g43310	LOC_Os12g34012
LOC_Os01g07880	LOC_Os06g06090	LOC_Os01g06876	LOC_Os02g01350	LOC_Os10g05180	LOC_Os01g12800	LOC_Os01g56680	LOC_Os12g33962
LOC_Os01g08180	LOC_Os11g06170	LOC_Os01g06920	LOC_Os05g44320	LOC_Os06g48640	LOC_Os01g53060	LOC_Os08g02630	LOC_Os12g33958
LOC_Os01g08710	LOC_Os12g06520	LOC_Os01g07110	LOC_Os11g04200	LOC_Os02g05340	LOC_Os03g38730	LOC_Os03g21560	LOC_Os12g34052
LOC_Os01g08790	LOC_Os02g27300	LOC_Os01g07120	LOC_Os12g04010	LOC_Os09g15750	LOC_Os09g14600	LOC_Os01g71190	LOC_Os12g34094
LOC_Os01g09080	LOC_Os01g28450	LOC_Os01g07600	LOC_Os08g05880	LOC_Os08g12820	LOC_Os12g32330	LOC_Os08g44680	LOC_Os12g34102
LOC_Os01g09100	LOC_Os06g24290	LOC_Os01g08440	LOC_Os05g08360	LOC_Os05g51640	LOC_Os09g33470	LOC_Os07g25430	LOC_Os12g34104
LOC_Os01g09550	LOC_Os07g14030	LOC_Os01g09010	LOC_Os02g57590	LOC_Os06g09290	LOC_Os03g15860	LOC_Os03g56670	LOC_Os06g08380
LOC_Os01g09720	LOC_Os01g28500	LOC_Os01g11230	LOC_Os07g46720	LOC_Os07g49150	LOC_Os02g13170	LOC_Os09g30340	LOC_Os12g33956
LOC_Os01g09900	LOC_Os07g03279	LOC_Os01g11350	LOC_Os03g22880	LOC_Os03g18690	LOC_Os01g32080	LOC_Os05g48630	LOC_Os01g57990
LOC_Os01g09930	LOC_Os07g03319	LOC_Os01g11730	LOC_Os02g30790	LOC_Os06g39870	LOC_Os05g39650	LOC_Os07g05480	LOC_Os01g58048
LOC_Os01g09940	LOC_Os07g03368	LOC_Os01g11970	LOC_Os11g37120	LOC_Os02g11050	LOC_Os03g50040	LOC_Os12g23200	LOC_Os04g16854
LOC_Os01g09990	LOC_Os07g03377	LOC_Os01g12110	LOC_Os12g20410	LOC_Os06g40560	LOC_Os01g06600	LOC_Os12g08770	LOC_Os01g42650
LOC_Os01g10610	LOC_Os07g03409	LOC_Os01g12280	LOC_Os03g22730	LOC_Os02g10640	LOC_Os06g24704	LOC_Os04g33830	LOC_Os12g34096
LOC_Os01g11350	LOC_Os07g03458	LOC_Os01g12580	LOC_Os03g22740	LOC_Os04g21660	LOC_Os11g39220	LOC_Os07g37030	LOC_Os12g34010
LOC_Os01g11550	LOC_Os07g03467	LOC_Os01g12680	LOC_Os11g34230	LOC_Os02g56000	LOC_Os06g01390	LOC_Os06g01210	LOC_Os12g34014
LOC_Os01g11910	LOC_Os07g03499	LOC_Os01g13080	LOC_Os03g13800	LOC_Os06g07630	LOC_Os05g07090	LOC_Os03g48040	LOC_Os12g34016
LOC_Os01g13460	LOC_Os07g03580	LOC_Os01g13390	LOC_Os10g03540	LOC_Os02g21970	LOC_Os06g23780	LOC_Os08g01380	LOC_Os11g37990
LOC_Os01g13540	LOC_Os07g03288	LOC_Os01g13690	LOC_Os04g48700	LOC_Os09g38730	LOC_Os06g23870	LOC_Os05g37140	LOC_Os12g08220
LOC_Os01g14030	LOC_Os07g03620	LOC_Os01g13760	LOC_Os11g38600	LOC_Os03g08280	LOC_Os10g31950	LOC_Os03g61960	LOC_Os12g34148
LOC_Os01g14420	LOC_Os07g03690	LOC_Os01g13950	LOC_Os03g25450	LOC_Os07g42260	LOC_Os02g57260	LOC_Os01g64120	LOC_Os12g33946
LOC_Os01g14440	LOC_Os07g03730	LOC_Os01g14410	LOC_Os07g44190	LOC_Os02g42320	LOC_Os04g52400	LOC_Os04g33630	LOC_Os01g58750
LOC_Os01g14720	LOC_Os10g11500	LOC_Os01g15340	LOC_Os06g16290	LOC_Os03g26970	LOC_Os01g70090	LOC_Os07g05400	LOC_Os01g67430
LOC_Os01g15640	LOC_Os07g03710	LOC_Os01g16170	LOC_Os02g49610	LOC_Os06g07978	LOC_Os11g35400	LOC_Os02g01340	LOC_Os02g15594
LOC_Os01g15900	LOC_Os01g43550	LOC_Os01g16714	LOC_Os11g37080	LOC_Os06g07140	LOC_Os05g04170	LOC_Os06g01850	LOC_Os12g33944
LOC_Os01g16660	LOC_Os04g48850	LOC_Os01g17402	LOC_Os02g40514	LOC_Os06g07878	LOC_Os01g46750	LOC_Os03g57120	LOC_Os12g33928
LOC_Os01g16740	LOC_Os02g39090	LOC_Os01g24710	LOC_Os06g06410	LOC_Os08g43540	LOC_Os01g48910	LOC_Os07g38000	LOC_Os12g34128
LOC_Os01g17000	LOC_Os04g41160	LOC_Os01g25484	LOC_Os03g22320	LOC_Os09g36710	LOC_Os06g06350	LOC_Os10g21264	LOC_Os02g08490
LOC_Os01g17260	LOC_Os07g30970	LOC_Os01g26039	LOC_Os02g18830	LOC_Os11g40140	LOC_Os03g62850	LOC_Os12g10570	LOC_Os01g47340
LOC_Os01g18584	LOC_Os05g51700	LOC_Os01g27360	LOC_Os03g20100	LOC_Os02g04100	LOC_Os12g04990	LOC_Os07g32880	LOC_Os05g44760
LOC_Os01g18850	LOC_Os10g41410	LOC_Os01g27490	LOC_Os01g40640	LOC_Os05g41180	LOC_Os11g04980	LOC_Os03g17070	LOC_Os01g53930
LOC_Os01g18870	LOC_Os12g36194	LOC_Os01g29280	LOC_Os05g45920	LOC_Os01g59600	LOC_Os12g07110	LOC_Os06g21590	LOC_Os12g34098
LOC_Os01g19050	LOC_Os08g32600	LOC_Os01g32380	LOC_Os02g01230	LOC_Os06g04800	LOC_Os11g06880	LOC_Os09g26810	LOC_Os12g34045
LOC_Os01g21590	LOC_Os06g27890	LOC_Os01g34790	LOC_Os03g34040	LOC_Os02g53060	LOC_Os05g25310	LOC_Os07g38960	LOC_Os12g17080
LOC_Os01g23840	LOC_Os02g05480	LOC_Os01g36580	LOC_Os03g62630	LOC_Os05g09490	LOC_Os06g04910	LOC_Os02g10390	LOC_Os05g44390
LOC_Os01g24070	LOC_Os06g48590	LOC_Os01g37690	LOC_Os01g12090	LOC_Os06g43570	LOC_Os09g23070	LOC_Os08g33820	LOC_Os04g08340
LOC_Os01g24460	LOC_Os03g49500	LOC_Os01g38110	LOC_Os12g34045	LOC_Os02g08520	LOC_Os08g20200	LOC_Os02g52650	LOC_Os04g22360
LOC_Os01g28050	LOC_Os04g08740	LOC_Os01g40280	LOC_Os07g46440	LOC_Os03g48930	LOC_Os07g30600	LOC_Os01g41710	LOC_Os04g57540
LOC_Os01g29840	LOC_Os05g06320	LOC_Os01g40290	LOC_Os03g55930	LOC_Os06g06030	LOC_Os08g44360	LOC_Os09g17740	LOC_Os04g35180
LOC_Os01g32770	LOC_Os07g15540	LOC_Os01g41900	LOC_Os03g10060	LOC_Os08g41730	LOC_Os09g39410	LOC_Os01g52240	LOC_Os04g53230
LOC_Os01g32890	LOC_Os02g57530	LOC_Os01g42380	LOC_Os12g33240	LOC_Os09g32800	LOC_Os03g07140	LOC_Os03g39610	LOC_Os04g55860
LOC_Os01g33400	LOC_Os02g32610	LOC_Os01g42790	LOC_Os03g26860	LOC_Os09g33986	LOC_Os04g28520	LOC_Os07g37550	LOC_Os04g46010
LOC_Os01g36220	LOC_Os09g39320	LOC_Os01g42860	LOC_Os03g49710	LOC_Os07g36420	LOC_Os04g28570	LOC_Os07g37240	LOC_Os04g30740
LOC_Os01g37100	LOC_Os05g46240	LOC_Os01g43280	LOC_Os08g40610	LOC_Os02g55640	LOC_Os04g28620	LOC_Os11g13890	LOC_Os04g01530
LOC_Os01g38610	LOC_Os03g58520	LOC_Os01g43480	LOC_Os09g32274	LOC_Os02g55710	LOC_Os10g18220	LOC_Os04g38410	LOC_Os04g32660
LOC_Os01g39020	LOC_Os01g51430	LOC_Os01g44970	LOC_Os08g27090	LOC_Os02g55670	LOC_Os08g39300	LOC_Os09g12540	LOC_Os04g44550
LOC_Os01g39040	LOC_Os06g45500	LOC_Os01g45840	LOC_Os04g59494	LOC_Os03g38720	LOC_Os01g46610	LOC_Os03g02480	LOC_Os04g37600
LOC_Os01g39070	LOC_Os02g10290	LOC_Os01g46260	LOC_Os01g48690	LOC_Os09g14590	LOC_Os04g42920	LOC_Os10g37690	LOC_Os02g45820
LOC_Os01g39150	LOC_Os04g46940	LOC_Os01g46350	LOC_Os03g48830	LOC_Os02g36974	LOC_Os05g49760	LOC_Os03g62750	LOC_Os10g42840
LOC_Os01g39160	LOC_Os02g07630	LOC_Os01g46850	LOC_Os03g59310	LOC_Os06g06050	LOC_Os01g14580	LOC_Os01g05800	LOC_Os10g41710
LOC_Os01g39180	LOC_Os03g08070	LOC_Os01g46910	LOC_Os07g10660	LOC_Os01g65920	LOC_Os01g21380	LOC_Os11g08980	LOC_Os07g46440
LOC_Os01g39220	LOC_Os08g39830	LOC_Os01g47780	LOC_Os07g41750	LOC_Os04g57290	LOC_Os09g32290	LOC_Os01g21820	LOC_Os07g22720
LOC_Os01g39330	LOC_Os04g38400	LOC_Os01g48740	LOC_Os03g38000	LOC_Os03g61670	LOC_Os12g35890	LOC_Os05g43390	LOC_Os07g38270
LOC_Os01g39480	LOC_Os03g20780	LOC_Os01g48874	LOC_Os12g21798	LOC_Os04g32460	LOC_Os11g04210	LOC_Os01g56600	LOC_Os07g30790
LOC_Os01g39580	LOC_Os03g20790	LOC_Os01g49560	LOC_Os03g10340	LOC_Os04g30200	LOC_Os01g16350	LOC_Os11g05556	LOC_Os10g26690
LOC_Os01g39850	LOC_Os07g48630	LOC_Os01g49720	LOC_Os02g18550	LOC_Os04g32950	LOC_Os03g57220	LOC_Os01g72800	LOC_Os03g48080
LOC_Os01g40260	LOC_Os09g31400	LOC_Os01g50110	LOC_Os01g25610	LOC_Os04g37590	LOC_Os04g53210	LOC_Os09g33720	LOC_Os01g49190
LOC_Os01g40430	LOC_Os03g49400	LOC_Os01g50350	LOC_Os02g01560	LOC_Os07g07240	LOC_Os07g42440	LOC_Os09g17830	LOC_Os01g14820
LOC_Os01g43550	LOC_Os07g06130	LOC_Os01g50370	LOC_Os05g30530	LOC_Os10g30200	LOC_Os07g05820	LOC_Os09g17840	LOC_Os04g38900
LOC_Os01g43590	LOC_Os02g10700	LOC_Os01g50610	LOC_Os01g01060	LOC_Os01g58670	LOC_Os06g51150	LOC_Os08g15460	LOC_Os09g19980
LOC_Os01g43650	LOC_Os06g40360	LOC_Os01g50616	LOC_Os11g29190	LOC_Os01g45900	LOC_Os02g02400	LOC_Os03g11440	LOC_Os04g54640
LOC_Os01g44430	LOC_Os03g58060	LOC_Os01g50622	LOC_Os03g27260	LOC_Os09g12150	LOC_Os03g03910	LOC_Os03g48590	LOC_Os07g07470
LOC_Os01g45860	LOC_Os09g39850	LOC_Os01g50910	LOC_Os07g42950	LOC_Os06g38940	LOC_Os08g44770	LOC_Os11g01300	LOC_Os02g37930
LOC_Os01g46800	LOC_Os03g64260	LOC_Os01g51040	LOC_Os05g27940	LOC_Os07g43220	LOC_Os03g11960	LOC_Os12g07490	LOC_Os04g42430
LOC_Os01g46970	LOC_Os05g33130	LOC_Os01g51754	LOC_Os03g18570	LOC_Os04g42470	LOC_Os07g46990	LOC_Os12g01260	LOC_Os04g42920
LOC_Os01g47360	LOC_Os05g33140	LOC_Os01g52130	LOC_Os03g18580	LOC_Os04g42670	LOC_Os03g22810	LOC_Os01g38510	LOC_Os04g43690
LOC_Os01g47560	LOC_Os05g33150	LOC_Os01g52230	LOC_Os02g21900	LOC_Os04g41470	LOC_Os06g02500	LOC_Os02g08180	LOC_Os04g45490
LOC_Os01g47670	LOC_Os03g04060	LOC_Os01g52260	LOC_Os04g28180	LOC_Os04g48520	LOC_Os05g25850	LOC_Os06g44374	LOC_Os04g29424
LOC_Os01g48130	LOC_Os06g51050	LOC_Os01g52340	LOC_Os02g28810	LOC_Os04g51110	LOC_Os06g05110	LOC_Os02g23890	LOC_Os04g32650
LOC_Os01g48290	LOC_Os03g30470	LOC_Os01g52380	LOC_Os11g38959	LOC_Os04g30210	LOC_Os03g31550	LOC_Os04g24180	LOC_Os04g37720
LOC_Os01g48320	LOC_Os10g39680	LOC_Os01g52530	LOC_Os03g26229	LOC_Os04g30320	LOC_Os09g04730	LOC_Os05g35400	LOC_Os01g46070
LOC_Os01g48446	LOC_Os10g39700	LOC_Os01g52680	LOC_Os03g05980	LOC_Os04g39040	LOC_Os07g29620	LOC_Os06g10990	LOC_Os09g24412
LOC_Os01g48470	LOC_Os06g51060	LOC_Os01g52900	LOC_Os04g35090	LOC_Os02g08300	LOC_Os08g32870	LOC_Os08g09770	LOC_Os10g21266
LOC_Os01g48700	LOC_Os10g42430	LOC_Os01g53220	LOC_Os02g34460	LOC_Os06g05760	LOC_Os04g39020	LOC_Os05g30480	LOC_Os05g47980
LOC_Os01g50940	LOC_Os06g33480	LOC_Os01g53370	LOC_Os01g73160	LOC_Os06g07000	LOC_Os08g35740	LOC_Os02g02410	LOC_Os05g33210
LOC_Os01g51140	LOC_Os06g33690	LOC_Os01g53640	LOC_Os04g42380	LOC_Os06g11630	LOC_Os04g57550	LOC_Os05g51360	LOC_Os05g31770
LOC_Os01g51690	LOC_Os02g15620	LOC_Os01g54030	LOC_Os04g52361	LOC_Os06g43710	LOC_Os04g57560	LOC_Os12g05740	LOC_Os03g07290
LOC_Os01g52090	LOC_Os02g15640	LOC_Os01g55200	LOC_Os07g12650	LOC_Os04g50720	LOC_Os02g17390	LOC_Os01g33360	LOC_Os10g42170
LOC_Os01g53040	LOC_Os03g18600	LOC_Os01g55260	LOC_Os07g05580	LOC_Os03g47770	LOC_Os04g53190	LOC_Os03g50250	LOC_Os10g42090
LOC_Os01g53220	LOC_Os01g61210	LOC_Os01g55270	LOC_Os07g38540	LOC_Os03g47550	LOC_Os04g53214	LOC_Os04g53220	LOC_Os01g14580
LOC_Os01g53260	LOC_Os02g13330	LOC_Os01g55450	LOC_Os02g45490	LOC_Os02g42412	LOC_Os07g34520	LOC_Os01g66560	LOC_Os06g43850
LOC_Os01g54210	LOC_Os05g12260	LOC_Os01g55700	LOC_Os08g02400	LOC_Os05g02650	LOC_Os04g45210	LOC_Os08g23360	LOC_Os09g28100
LOC_Os01g54550	LOC_Os06g36670	LOC_Os01g55974	LOC_Os08g02410	LOC_Os03g47500	LOC_Os09g31970	LOC_Os06g23430	LOC_Os06g44230
LOC_Os01g54600	LOC_Os05g39580	LOC_Os01g56090	LOC_Os02g33140	LOC_Os03g05730	LOC_Os09g14510	LOC_Os08g37444	LOC_Os06g01630
LOC_Os01g54930	LOC_Os10g42280	LOC_Os01g56780	LOC_Os02g06700	LOC_Os08g06510	LOC_Os08g40180	LOC_Os08g41250	LOC_Os06g02600
LOC_Os01g55150	LOC_Os06g33640	LOC_Os01g58420	LOC_Os03g58430	LOC_Os03g63520	LOC_Os05g31770	LOC_Os06g27870	LOC_Os06g01400
LOC_Os01g55340	LOC_Os01g46760	LOC_Os01g59020	LOC_Os07g10720	LOC_Os09g30390	LOC_Os06g03660	LOC_Os03g43430	LOC_Os01g59060
LOC_Os01g55580	LOC_Os01g40094	LOC_Os01g59150	LOC_Os02g15610	LOC_Os02g44510	LOC_Os03g24060	LOC_Os01g31680	LOC_Os10g37530
LOC_Os01g55750	LOC_Os05g49730	LOC_Os01g60000	LOC_Os02g27760	LOC_Os03g04270	LOC_Os03g19010	LOC_Os09g28000	LOC_Os01g19548
LOC_Os01g56530	LOC_Os03g16170	LOC_Os01g60020	LOC_Os01g06000	LOC_Os03g03420	LOC_Os03g02590	LOC_Os02g16709	LOC_Os09g38100
LOC_Os01g56690	LOC_Os05g46040	LOC_Os01g60730	LOC_Os11g03400	LOC_Os03g03150	LOC_Os03g04000	LOC_Os12g25210	LOC_Os04g51280
LOC_Os01g57580	LOC_Os01g62760	LOC_Os01g61610	LOC_Os12g03090	LOC_Os03g15930	LOC_Os03g14830	LOC_Os02g35720	LOC_Os04g41530
LOC_Os01g58760	LOC_Os09g15670	LOC_Os01g62020	LOC_Os03g01900	LOC_Os03g15880	LOC_Os02g48330	LOC_Os04g36859	LOC_Os10g42540
LOC_Os01g59350	LOC_Os03g55600	LOC_Os01g62260	LOC_Os10g27190	LOC_Os03g13220	LOC_Os03g19680	LOC_Os01g04030	LOC_Os10g34750
LOC_Os01g59640	LOC_Os01g64970	LOC_Os01g62410	LOC_Os03g58050	LOC_Os03g13100	LOC_Os03g19000	LOC_Os09g38370	LOC_Os03g38490
LOC_Os01g59760	LOC_Os03g41460	LOC_Os01g62810	LOC_Os07g07709	LOC_Os07g15490	LOC_Os05g01700	LOC_Os05g23260	LOC_Os03g41530
LOC_Os01g60020	LOC_Os03g27280	LOC_Os01g62980	LOC_Os07g07719	LOC_Os05g05210	LOC_Os01g10830	LOC_Os06g16260	LOC_Os03g25050
LOC_Os01g60490	LOC_Os04g35240	LOC_Os01g63160	LOC_Os07g07770	LOC_Os09g24200	LOC_Os05g06300	LOC_Os02g58139	LOC_Os03g24060
LOC_Os01g60520	LOC_Os05g35760	LOC_Os01g63190	LOC_Os03g31090	LOC_Os05g05800	LOC_Os09g36300	LOC_Os11g40500	LOC_Os03g25400
LOC_Os01g60540	LOC_Os02g34600	LOC_Os01g64010	LOC_Os03g31134	LOC_Os05g08960	LOC_Os01g73530	LOC_Os04g08340	LOC_Os03g24440
LOC_Os01g60600	LOC_Os04g59450	LOC_Os01g65100	LOC_Os06g04290	LOC_Os05g11850	LOC_Os09g37280	LOC_Os03g05430	LOC_Os03g59220
LOC_Os01g60640	LOC_Os10g41490	LOC_Os01g65150	LOC_Os03g14530	LOC_Os05g39380	LOC_Os08g21590	LOC_Os01g16520	LOC_Os03g26860
LOC_Os01g60960	LOC_Os07g42940	LOC_Os01g65169	LOC_Os10g08930	LOC_Os05g43170	LOC_Os08g04770	LOC_Os02g02860	LOC_Os12g07700
LOC_Os01g61080	LOC_Os12g39630	LOC_Os01g65420	LOC_Os03g22460	LOC_Os09g25260	LOC_Os01g11946	LOC_Os10g22380	LOC_Os03g30200
LOC_Os01g61480	LOC_Os05g46750	LOC_Os01g65690	LOC_Os03g46490	LOC_Os09g33876	LOC_Os05g50220	LOC_Os10g35840	LOC_Os10g10244
LOC_Os01g61810	LOC_Os06g51150	LOC_Os01g66240	LOC_Os10g20990	LOC_Os12g24080	LOC_Os03g48480	LOC_Os08g41990	LOC_Os10g10434
LOC_Os01g62460	LOC_Os02g02400	LOC_Os01g66980	LOC_Os03g60400	LOC_Os12g34220	LOC_Os01g24680	LOC_Os06g49110	LOC_Os03g62490
LOC_Os01g62510	LOC_Os03g03910	LOC_Os01g67390	LOC_Os01g61814	LOC_Os08g35060	LOC_Os05g08810	LOC_Os02g07230	LOC_Os03g63010
LOC_Os01g62514	LOC_Os12g04580	LOC_Os01g68650	LOC_Os06g36160	LOC_Os11g41990	LOC_Os05g32630	LOC_Os03g08730	LOC_Os03g63500
LOC_Os01g63030	LOC_Os01g17190	LOC_Os01g68790	LOC_Os02g13530	LOC_Os09g07900	LOC_Os05g29880	LOC_Os01g43390	LOC_Os03g53500
LOC_Os01g63380	LOC_Os11g37550	LOC_Os01g69020	LOC_Os01g52490	LOC_Os03g45770	LOC_Os05g45300	LOC_Os03g21900	LOC_Os03g18420
LOC_Os01g64000	LOC_Os01g16240	LOC_Os01g69030	LOC_Os09g39540	LOC_Os03g43770	LOC_Os01g72280	LOC_Os03g22060	LOC_Os03g18500
LOC_Os01g64020	LOC_Os11g03980	LOC_Os01g71040	LOC_Os11g05562	LOC_Os03g31400	LOC_Os10g42800	LOC_Os04g52130	LOC_Os07g32880
LOC_Os01g64310	LOC_Os03g20370	LOC_Os01g72360	LOC_Os08g44480	LOC_Os05g35400	LOC_Os08g09860	LOC_Os04g41260	LOC_Os03g20750
LOC_Os01g64560	LOC_Os05g41200	LOC_Os01g72370	LOC_Os01g60790	LOC_Os03g42760	LOC_Os07g44560	LOC_Os01g18320	LOC_Os03g21950
LOC_Os01g64590	LOC_Os05g41210	LOC_Os01g72460	LOC_Os05g39960	LOC_Os03g42790	LOC_Os08g44240	LOC_Os09g12560	LOC_Os03g23000
LOC_Os01g64730	LOC_Os01g59530	LOC_Os01g72530	LOC_Os04g27860	LOC_Os07g22680	LOC_Os03g48160	LOC_Os05g29760	LOC_Os07g28400
LOC_Os01g65900	LOC_Os12g03816	LOC_Os01g72900	LOC_Os02g27769	LOC_Os08g28820	LOC_Os05g04660	LOC_Os01g44050	LOC_Os07g33680
LOC_Os01g66120	LOC_Os01g25820	LOC_Os01g73200	LOC_Os05g06770	LOC_Os07g05180	LOC_Os12g07880	LOC_Os02g19440	LOC_Os03g02030
LOC_Os01g66490	LOC_Os05g45210	LOC_Os01g73720	LOC_Os01g22490	LOC_Os08g42740	LOC_Os07g41810	LOC_Os01g34390	LOC_Os03g01216
LOC_Os01g66590	LOC_Os01g53294	LOC_Os01g73980	LOC_Os10g34990	LOC_Os02g03820	LOC_Os05g41810	LOC_Os08g38720	LOC_Os03g02260
LOC_Os01g67480	LOC_Os08g35210	LOC_Os02g01190	LOC_Os10g27174	LOC_Os08g33340		LOC_Os03g59640	LOC_Os03g03820
LOC_Os01g67650	LOC_Os12g35610	LOC_Os02g01590	LOC_Os03g56241	LOC_Os08g28800		LOC_Os03g36540	LOC_Os03g04970
LOC_Os01g67670	LOC_Os09g26660	LOC_Os02g02030	LOC_Os11g41610	LOC_Os08g35700		LOC_Os06g04150	LOC_Os03g04410
LOC_Os01g68700	LOC_Os04g38470	LOC_Os02g02140	LOC_Os12g32380	LOC_Os08g02140		LOC_Os01g17170	LOC_Os03g04460
LOC_Os01g69830	LOC_Os04g54490	LOC_Os02g02340	LOC_Os02g56014	LOC_Os02g47180		LOC_Os03g22780	LOC_Os03g63680
LOC_Os01g69910	LOC_Os06g03970	LOC_Os02g02930	LOC_Os06g07580	LOC_Os02g13180		LOC_Os04g58200	LOC_Os08g06430
LOC_Os01g69980	LOC_Os06g10230	LOC_Os02g03710	LOC_Os07g42450	LOC_Os02g52130		LOC_Os05g28200	LOC_Os03g10060
LOC_Os01g70110	LOC_Os04g47240	LOC_Os02g04780	LOC_Os03g28410	LOC_Os02g50680		LOC_Os10g28370	LOC_Os03g10850
LOC_Os01g70230	LOC_Os02g44642	LOC_Os02g06290	LOC_Os05g32220	LOC_Os02g21240		LOC_Os10g41760	LOC_Os03g11120
LOC_Os01g70310	LOC_Os02g15760	LOC_Os02g06410	LOC_Os02g04460	LOC_Os02g21260		LOC_Os10g41780	LOC_Os03g11410
LOC_Os01g70880	LOC_Os06g33450	LOC_Os02g06930	LOC_Os01g14830	LOC_Os01g63420		LOC_Os01g12710	LOC_Os03g58040
LOC_Os01g70890	LOC_Os03g50320	LOC_Os02g07430	LOC_Os03g15870	LOC_Os02g02410		LOC_Os03g45194	LOC_Os03g63410
LOC_Os01g71790	LOC_Os11g20384	LOC_Os02g07930	LOC_Os11g37510	LOC_Os08g09640		LOC_Os04g25400	LOC_Os03g63420
LOC_Os01g71850	LOC_Os02g34884	LOC_Os02g08230	LOC_Os03g03360	LOC_Os08g09650		LOC_Os04g59610	LOC_Os03g16440
LOC_Os01g71970	LOC_Os03g18080	LOC_Os02g09240	LOC_Os03g54040	LOC_Os08g09770		LOC_Os09g36200	LOC_Os03g58540
LOC_Os01g72370	LOC_Os03g49800	LOC_Os02g09810	LOC_Os03g51510	LOC_Os05g09550		LOC_Os03g05310	LOC_Os03g14890
LOC_Os01g72490	LOC_Os07g46490	LOC_Os02g09830	LOC_Os03g24020	LOC_Os09g38630		LOC_Os10g25030	LOC_Os03g15240
LOC_Os01g74140	LOC_Os03g24160	LOC_Os02g09940	LOC_Os03g52400	LOC_Os02g39970		LOC_Os10g25020	LOC_Os03g10510
LOC_Os01g74540	LOC_Os12g04700	LOC_Os02g10120	LOC_Os05g49320	LOC_Os02g26670		LOC_Os10g25040	LOC_Os03g34040
LOC_Os02g01380	LOC_Os02g57660	LOC_Os02g10240	LOC_Os05g48050	LOC_Os01g70140		LOC_Os01g16020	LOC_Os05g06750
LOC_Os02g01860	LOC_Os03g49510	LOC_Os02g10760	LOC_Os07g43310	LOC_Os12g43930		LOC_Os02g51080	LOC_Os02g39950
LOC_Os02g01950	LOC_Os11g04840	LOC_Os02g10800	LOC_Os02g10540	LOC_Os12g25640		LOC_Os06g40080	LOC_Os02g49360
LOC_Os02g02480	LOC_Os11g04900	LOC_Os02g11110	LOC_Os05g45270	LOC_Os01g16650		LOC_Os01g72090	LOC_Os03g03770
LOC_Os02g02820	LOC_Os03g15550	LOC_Os02g11760	LOC_Os02g57670	LOC_Os01g61420		LOC_Os01g57460	LOC_Os06g10810
LOC_Os02g03580	LOC_Os12g21890	LOC_Os02g12370	LOC_Os05g03030	LOC_Os01g74610		LOC_Os02g33610	LOC_Os05g44916
LOC_Os02g03690	LOC_Os08g33200	LOC_Os02g12610	LOC_Os03g17580	LOC_Os06g15360		LOC_Os01g54540	LOC_Os06g22654
LOC_Os02g03960	LOC_Os09g10650	LOC_Os02g13800	LOC_Os10g32870	LOC_Os06g42810		LOC_Os02g15900	LOC_Os05g25850
LOC_Os02g04340	LOC_Os08g01390	LOC_Os02g14430	LOC_Os03g03020	LOC_Os06g40360		LOC_Os11g05552	LOC_Os03g22950
LOC_Os02g04680	LOC_Os09g23740	LOC_Os02g14440	LOC_Os01g54540	LOC_Os09g11170		LOC_Os03g03990	LOC_Os03g20560
LOC_Os02g05510	LOC_Os12g13440	LOC_Os02g14600	LOC_Os05g15350	LOC_Os12g37720		LOC_Os04g57920	LOC_Os03g20010
LOC_Os02g06370	LOC_Os04g59540	LOC_Os02g14790	LOC_Os05g15360	LOC_Os12g40300		LOC_Os03g61570	LOC_Os03g11510
LOC_Os02g06950	LOC_Os08g44260	LOC_Os02g15460	LOC_Os05g15370	LOC_Os12g43560		LOC_Os06g45640	LOC_Os03g09210
LOC_Os02g07450	LOC_Os03g18000	LOC_Os02g15930	LOC_Os08g06474	LOC_Os12g44000		LOC_Os06g16370	LOC_Os03g61330
LOC_Os02g07780	LOC_Os07g49330	LOC_Os02g16909	LOC_Os12g42180	LOC_Os09g26460		LOC_Os03g05690	LOC_Os01g55540
LOC_Os02g07800	LOC_Os05g03610	LOC_Os02g16940	LOC_Os08g21840	LOC_Os06g10990		LOC_Os03g61220	LOC_Os03g50940
LOC_Os02g07840	LOC_Os12g37560	LOC_Os02g18410	LOC_Os03g12020	LOC_Os09g36830		LOC_Os11g14130	LOC_Os01g68790
LOC_Os02g08070	LOC_Os02g32370	LOC_Os02g19850	LOC_Os01g62210	LOC_Os06g45460		LOC_Os11g10040	LOC_Os07g44940
LOC_Os02g08440	LOC_Os02g26720	LOC_Os02g21700	LOC_Os05g38580	LOC_Os01g01484		LOC_Os11g10000	LOC_Os01g46610
LOC_Os02g08540	LOC_Os10g01480	LOC_Os02g22020	LOC_Os03g60100	LOC_Os02g52230		LOC_Os11g09990	LOC_Os07g31380
LOC_Os02g09830	LOC_Os03g12840	LOC_Os02g26810	LOC_Os02g46440	LOC_Os07g14270		LOC_Os12g17310	LOC_Os04g40650
LOC_Os02g10140	LOC_Os03g51610	LOC_Os02g26860	LOC_Os10g34320	LOC_Os01g69940		LOC_Os03g27800	LOC_Os03g62260
LOC_Os02g10360	LOC_Os10g42550	LOC_Os02g29340	LOC_Os02g43600	LOC_Os01g74146		LOC_Os11g43600	LOC_Os03g61019
LOC_Os02g10840	LOC_Os04g56580	LOC_Os02g30310	LOC_Os02g32400	LOC_Os02g42690		LOC_Os03g11190	LOC_Os07g13590
LOC_Os02g10860	LOC_Os01g56980	LOC_Os02g30650	LOC_Os01g53280	LOC_Os01g05090		LOC_Os03g08080	LOC_Os07g08950
LOC_Os02g12310	LOC_Os03g48300	LOC_Os02g30780	LOC_Os05g45220	LOC_Os06g02360		LOC_Os03g22040	LOC_Os04g51260
LOC_Os02g12790	LOC_Os12g08270	LOC_Os02g32060	LOC_Os05g48410	LOC_Os02g44104		LOC_Os03g44420	LOC_Os07g23244
LOC_Os02g12820	LOC_Os12g08280	LOC_Os02g32550	LOC_Os02g15900	LOC_Os09g10054		LOC_Os03g24590	LOC_Os07g15880
LOC_Os02g13670	LOC_Os02g07350	LOC_Os02g32650	LOC_Os01g42980	LOC_Os09g10270		LOC_Os12g01449	LOC_Os02g10070
LOC_Os02g13800	LOC_Os03g39000	LOC_Os02g33380	LOC_Os07g23244	LOC_Os09g10300		LOC_Os11g09040	LOC_Os04g38330
LOC_Os02g13900	LOC_Os07g09330	LOC_Os02g33590	LOC_Os11g04370	LOC_Os05g30480		LOC_Os04g02650	LOC_Os04g47900
LOC_Os02g14910	LOC_Os04g54200	LOC_Os02g33710	LOC_Os06g46930	LOC_Os05g33710		LOC_Os04g02580	LOC_Os04g46460
LOC_Os02g15340	LOC_Os08g08110	LOC_Os02g34470	LOC_Os12g04160	LOC_Os02g40810		LOC_Os12g30610	LOC_Os03g19890
LOC_Os02g15350	LOC_Os02g54650	LOC_Os02g35000	LOC_Os03g41612	LOC_Os05g06760		LOC_Os04g49540	LOC_Os01g40570
LOC_Os02g15760	LOC_Os03g31180	LOC_Os02g35329	LOC_Os01g41030	LOC_Os03g57790		LOC_Os12g08160	LOC_Os03g29180
LOC_Os02g16140	LOC_Os01g57420	LOC_Os02g36110	LOC_Os05g18750	LOC_Os01g37560		LOC_Os12g02385	LOC_Os10g17280
LOC_Os02g16240	LOC_Os12g38780	LOC_Os02g36190	LOC_Os08g31228	LOC_Os09g10230		LOC_Os04g35900	LOC_Os09g31486
LOC_Os02g16540	LOC_Os10g17990	LOC_Os02g36280	LOC_Os01g69950	LOC_Os09g10200		LOC_Os02g36780	LOC_Os07g07060
LOC_Os02g16680	LOC_Os01g55360	LOC_Os02g36450	LOC_Os08g31219	LOC_Os02g43920		LOC_Os01g60830	LOC_Os07g10300
LOC_Os02g17460	LOC_Os02g03110	LOC_Os02g36570	LOC_Os04g25540	LOC_Os09g10260		LOC_Os02g05880	LOC_Os07g10500
LOC_Os02g18370	LOC_Os03g14080	LOC_Os02g36740	LOC_Os05g01110	LOC_Os05g37690		LOC_Os06g12040	LOC_Os07g09450
LOC_Os02g18460	LOC_Os10g05690	LOC_Os02g37000	LOC_Os07g36090	LOC_Os05g35110		LOC_Os06g12060	LOC_Os06g35540
LOC_Os02g18470	LOC_Os01g63770	LOC_Os02g37190	LOC_Os02g51790	LOC_Os05g01460		LOC_Os06g12080	LOC_Os06g44880
LOC_Os02g18660	LOC_Os05g37470	LOC_Os02g37480	LOC_Os08g01859	LOC_Os05g05720		LOC_Os06g12100	LOC_Os07g32550
LOC_Os02g21685	LOC_Os11g06820	LOC_Os02g37654	LOC_Os06g23210	LOC_Os01g35040		LOC_Os06g12110	LOC_Os07g39070
LOC_Os02g23823	LOC_Os03g53150	LOC_Os02g38430	LOC_Os01g44210	LOC_Os01g74360		LOC_Os01g48380	LOC_Os07g47300
LOC_Os02g25470	LOC_Os02g57250	LOC_Os02g39000	LOC_Os10g26690	LOC_Os07g43180		LOC_Os01g39890	LOC_Os05g23620
LOC_Os02g26430	LOC_Os03g58350	LOC_Os02g39260	LOC_Os03g07290	LOC_Os05g30920		LOC_Os02g03000	LOC_Os05g29760
LOC_Os02g26780	LOC_Os06g39590	LOC_Os02g39710	LOC_Os05g37884	LOC_Os11g29340		LOC_Os11g01439	LOC_Os05g34770
LOC_Os02g28180	LOC_Os03g43410	LOC_Os02g39730	LOC_Os01g39010	LOC_Os11g31620		LOC_Os03g57149	LOC_Os05g38400
LOC_Os02g29340	LOC_Os12g40890	LOC_Os02g39930	LOC_Os01g59060	LOC_Os12g33220		LOC_Os07g48080	LOC_Os05g43770
LOC_Os02g29640	LOC_Os02g13520	LOC_Os02g40510	LOC_Os06g43900	LOC_Os12g33230		LOC_Os05g49820	LOC_Os01g21250
LOC_Os02g31160	LOC_Os01g09450	LOC_Os02g40710	LOC_Os03g59720	LOC_Os11g38710		LOC_Os05g33020	LOC_Os01g34620
LOC_Os02g31850	LOC_Os11g11410	LOC_Os02g40770	LOC_Os12g07010	LOC_Os10g24070		LOC_Os05g39840	LOC_Os01g41610
LOC_Os02g32590	LOC_Os08g01780	LOC_Os02g40900	LOC_Os11g06750	LOC_Os09g25190		LOC_Os01g46340	LOC_Os09g28160
LOC_Os02g33430	LOC_Os05g44810	LOC_Os02g41840	LOC_Os07g08330	LOC_Os03g50250		LOC_Os11g02460	LOC_Os09g33480
LOC_Os02g33560	LOC_Os05g48590	LOC_Os02g41860	LOC_Os03g58204	LOC_Os02g10700		LOC_Os09g38268	LOC_Os09g33500
LOC_Os02g33610	LOC_Os01g13030	LOC_Os02g41904	LOC_Os01g67134	LOC_Os11g16280		LOC_Os09g38330	LOC_Os12g18630
LOC_Os02g33750	LOC_Os05g08570	LOC_Os02g42374	LOC_Os02g37862	LOC_Os03g44830		LOC_Os01g49740	LOC_Os12g41500
LOC_Os02g33770	LOC_Os01g08320	LOC_Os02g42740	LOC_Os04g39700	LOC_Os01g28680		LOC_Os04g54510	LOC_Os01g07950
LOC_Os02g34320	LOC_Os05g14180	LOC_Os02g42800	LOC_Os08g42920	LOC_Os07g05150		LOC_Os07g08770	LOC_Os08g14860
LOC_Os02g34370	LOC_Os07g08460	LOC_Os02g42880	LOC_Os08g13690	LOC_Os04g44820		LOC_Os09g02440	LOC_Os08g15170
LOC_Os02g34520	LOC_Os03g43400	LOC_Os02g43330	LOC_Os04g51630	LOC_Os07g43230		LOC_Os02g51460	LOC_Os11g02460
LOC_Os02g34590	LOC_Os06g07040	LOC_Os02g43519	LOC_Os09g32976	LOC_Os07g43250		LOC_Os07g24090	LOC_Os11g06340
LOC_Os02g34970	LOC_Os02g56120	LOC_Os02g44300	LOC_Os08g23710	LOC_Os05g51360		LOC_Os03g18300	LOC_Os11g07916
LOC_Os02g35660	LOC_Os06g22870	LOC_Os02g44630	LOC_Os01g70010	LOC_Os11g01240		LOC_Os07g38900	LOC_Os08g32850
LOC_Os02g35690	LOC_Os11g11430	LOC_Os02g44820	LOC_Os12g38000	LOC_Os11g01780		LOC_Os08g41690	LOC_Os08g33460
LOC_Os02g36510	LOC_Os11g11420	LOC_Os02g44990	LOC_Os09g31180	LOC_Os12g36670		LOC_Os04g56970	LOC_Os08g33720
LOC_Os02g36880	LOC_Os12g40900	LOC_Os02g45010	LOC_Os02g01332	LOC_Os12g40860		LOC_Os09g28450	LOC_Os11g40500
LOC_Os02g38130	LOC_Os01g53880	LOC_Os02g45160	LOC_Os05g07700	LOC_Os12g40390		LOC_Os05g37160	LOC_Os12g01160
LOC_Os02g39140	LOC_Os09g35870	LOC_Os02g46030	LOC_Os11g11390	LOC_Os11g19500		LOC_Os02g34870	LOC_Os01g51170
LOC_Os02g39220	LOC_Os01g48444	LOC_Os02g46473	LOC_Os08g44380	LOC_Os12g01760		LOC_Os06g22660	LOC_Os10g15390
LOC_Os02g39520	LOC_Os01g18360	LOC_Os02g46500	LOC_Os08g44450	LOC_Os12g01230		LOC_Os03g55164	LOC_Os10g35730
LOC_Os02g39540	LOC_Os05g05800	LOC_Os02g46660	LOC_Os01g64090	LOC_Os11g42160		LOC_Os05g43470	LOC_Os03g43420
LOC_Os02g40950	LOC_Os05g43920	LOC_Os02g46680	LOC_Os01g10820	LOC_Os10g05800		LOC_Os05g33440	LOC_Os03g30230
LOC_Os02g41450	LOC_Os02g35140	LOC_Os02g46970	LOC_Os06g35730	LOC_Os09g33740		LOC_Os05g33460	LOC_Os05g03540
LOC_Os02g42380	LOC_Os01g48060	LOC_Os02g47090	LOC_Os05g11710	LOC_Os08g39060		LOC_Os05g34160	LOC_Os03g49710
LOC_Os02g42950	LOC_Os04g36054	LOC_Os02g47200	LOC_Os02g14059	LOC_Os01g05500		LOC_Os01g63220	LOC_Os03g61920
LOC_Os02g42970	LOC_Os01g54990	LOC_Os02g47370	LOC_Os04g50990	LOC_Os01g67054		LOC_Os05g32680	LOC_Os04g57950
LOC_Os02g43150	LOC_Os04g56850	LOC_Os02g47420	LOC_Os02g47140	LOC_Os02g28830		LOC_Os02g51450	LOC_Os03g31150
LOC_Os02g43300	LOC_Os05g48870	LOC_Os02g47560	LOC_Os03g37970	LOC_Os02g17990		LOC_Os07g09040	LOC_Os08g21840
LOC_Os02g43560	LOC_Os07g40290	LOC_Os02g47620	LOC_Os06g02510	LOC_Os02g08480		LOC_Os05g47680	LOC_Os03g10930
LOC_Os02g44360	LOC_Os01g57610	LOC_Os02g47660	LOC_Os03g54860	LOC_Os02g01160		LOC_Os05g25060	LOC_Os07g45090
LOC_Os02g44370	LOC_Os07g38860	LOC_Os02g47840	LOC_Os03g54890	LOC_Os03g13370		LOC_Os08g03560	LOC_Os07g43700
LOC_Os02g45200	LOC_Os11g32510	LOC_Os02g48140	LOC_Os07g01870	LOC_Os03g59264		LOC_Os06g05060	LOC_Os07g31300
LOC_Os02g45760	LOC_Os01g12160	LOC_Os02g48200	LOC_Os02g40880	LOC_Os03g01660		LOC_Os06g06300	LOC_Os06g01470
LOC_Os02g46560	LOC_Os01g55940	LOC_Os02g48770	LOC_Os05g19370	LOC_Os02g54490		LOC_Os03g19480	LOC_Os08g02540
LOC_Os02g47060	LOC_Os05g05180	LOC_Os02g49230	LOC_Os03g40180	LOC_Os03g42780		LOC_Os01g69850	LOC_Os08g44250
LOC_Os02g47660	LOC_Os05g42150	LOC_Os02g50040	LOC_Os08g41810	LOC_Os03g43390		LOC_Os10g32600	LOC_Os04g25410
LOC_Os02g47770	LOC_Os06g30440	LOC_Os02g50372	LOC_Os09g08430	LOC_Os02g46140		LOC_Os03g55389	LOC_Os02g35039
LOC_Os02g47810	LOC_Os11g08340	LOC_Os02g50460	LOC_Os07g47780	LOC_Os01g05120		LOC_Os09g13630	LOC_Os02g38200
LOC_Os02g48060	LOC_Os02g05050	LOC_Os02g50560	LOC_Os05g06310	LOC_Os01g56070		LOC_Os03g32314	LOC_Os02g38210
LOC_Os02g48270	LOC_Os08g02520	LOC_Os02g50990	LOC_Os03g22180	LOC_Os01g50840		LOC_Os02g18840	LOC_Os02g02940
LOC_Os02g49370	LOC_Os09g26610	LOC_Os02g51040	LOC_Os05g49030	LOC_Os07g43240		LOC_Os01g51610	LOC_Os02g48670
LOC_Os02g49410	LOC_Os04g52670	LOC_Os02g51710	LOC_Os01g54870	LOC_Os01g33360		LOC_Os08g07740	LOC_Os02g53770
LOC_Os02g49440	LOC_Os02g42990	LOC_Os02g51910	LOC_Os03g38260	LOC_Os05g30130		LOC_Os09g06464	LOC_Os08g14770
LOC_Os02g49480	LOC_Os02g30810	LOC_Os02g52150	LOC_Os03g21940	LOC_Os04g50740		LOC_Os07g49460	LOC_Os07g36090
LOC_Os02g49560	LOC_Os04g56680	LOC_Os02g52710	LOC_Os10g32820	LOC_Os09g31090		LOC_Os06g06320	LOC_Os08g04450
LOC_Os02g50630	LOC_Os04g56690	LOC_Os02g52780	LOC_Os03g04750	LOC_Os01g73750		LOC_Os06g41090	LOC_Os04g51350
LOC_Os02g51090	LOC_Os06g04590	LOC_Os02g52910	LOC_Os03g22340	LOC_Os06g41750		LOC_Os08g01420	LOC_Os07g40750
LOC_Os02g51280	LOC_Os02g52990	LOC_Os02g53130	LOC_Os07g47710	LOC_Os06g19730		LOC_Os03g14669	LOC_Os04g33060
LOC_Os02g51310	LOC_Os04g51890	LOC_Os02g53160	LOC_Os02g56960	LOC_Os06g35740		LOC_Os06g16390	LOC_Os07g04240
LOC_Os02g51320	LOC_Os06g45950	LOC_Os02g53200	LOC_Os10g32920	LOC_Os06g46540		LOC_Os03g60110	LOC_Os04g17050
LOC_Os02g52190	LOC_Os06g50040	LOC_Os02g53320	LOC_Os03g04590	LOC_Os07g42900		LOC_Os09g04890	LOC_Os08g42560
LOC_Os02g52780	LOC_Os04g45370	LOC_Os02g53384	LOC_Os01g24690	LOC_Os07g12620		LOC_Os07g15770	LOC_Os11g20790
LOC_Os02g52950	LOC_Os02g24740	LOC_Os02g53820	LOC_Os04g42270	LOC_Os07g03760		LOC_Os03g57940	LOC_Os12g13380
LOC_Os02g53100	LOC_Os04g43740	LOC_Os02g54060	LOC_Os07g19190	LOC_Os07g03160		LOC_Os03g02160	LOC_Os08g09200
LOC_Os02g53150	LOC_Os01g70050	LOC_Os02g54140	LOC_Os05g40820	LOC_Os06g46590		LOC_Os12g38210	LOC_Os08g44460
LOC_Os02g53620	LOC_Os10g36703	LOC_Os02g54254	LOC_Os01g33050	LOC_Os08g01040		LOC_Os02g41550	LOC_Os08g44470
LOC_Os02g55250	LOC_Os06g48850	LOC_Os02g54560	LOC_Os01g59990	LOC_Os08g31870		LOC_Os02g10990	LOC_Os02g49820
LOC_Os02g56140	LOC_Os12g43110	LOC_Os02g54710	LOC_Os07g12250	LOC_Os08g19830		LOC_Os05g43950	LOC_Os08g40230
LOC_Os02g56250	LOC_Os02g24700	LOC_Os02g54920	LOC_Os01g04730	LOC_Os08g09670		LOC_Os02g36380	LOC_Os08g33810
LOC_Os02g56600	LOC_Os03g56784	LOC_Os02g55134	LOC_Os12g05430	LOC_Os08g09590		LOC_Os01g08700	LOC_Os02g46990
LOC_Os02g57490	LOC_Os10g21810	LOC_Os02g55380	LOC_Os11g05370	LOC_Os08g09600		LOC_Os01g54930	LOC_Os07g11050
LOC_Os02g57650	LOC_Os03g50860	LOC_Os02g56150	LOC_Os02g18380	LOC_Os08g09410		LOC_Os04g37920	LOC_Os08g01859
LOC_Os02g58670	LOC_Os09g39400	LOC_Os02g56570	LOC_Os10g41470	LOC_Os08g09220		LOC_Os02g17380	LOC_Os08g23320
LOC_Os03g01870	LOC_Os05g44560	LOC_Os02g57010	LOC_Os02g07890	LOC_Os07g31540		LOC_Os02g05890	LOC_Os08g37320
LOC_Os03g02240	LOC_Os05g09410	LOC_Os02g57160	LOC_Os07g42170	LOC_Os04g51910		LOC_Os07g47300	LOC_Os04g57730
LOC_Os03g02800	LOC_Os01g54050	LOC_Os02g57720	LOC_Os03g29460	LOC_Os04g48770		LOC_Os02g12790	LOC_Os02g20844
LOC_Os03g03000	LOC_Os08g44350	LOC_Os02g58120	LOC_Os01g51020	LOC_Os04g50760		LOC_Os01g58470	LOC_Os02g53420
LOC_Os03g03540	LOC_Os02g08500	LOC_Os03g01270	LOC_Os02g57540			LOC_Os07g33660	LOC_Os02g35610
LOC_Os03g03550	LOC_Os01g67770	LOC_Os03g01800	LOC_Os05g46430			LOC_Os07g42790	LOC_Os07g19460
LOC_Os03g03850	LOC_Os02g55320	LOC_Os03g02040	LOC_Os05g28750			LOC_Os05g09380	LOC_Os02g20860
LOC_Os03g03900	LOC_Os03g12350	LOC_Os03g02050	LOC_Os01g19840			LOC_Os03g40550	LOC_Os02g01230
LOC_Os03g04070	LOC_Os05g32880	LOC_Os03g02210	LOC_Os05g41110			LOC_Os02g52744	LOC_Os08g38720
LOC_Os03g04310	LOC_Os06g08440	LOC_Os03g02650	LOC_Os01g16890			LOC_Os08g21700	LOC_Os08g31830
LOC_Os03g05160	LOC_Os06g43910	LOC_Os03g02660	LOC_Os01g59730			LOC_Os08g40430	LOC_Os07g41330
LOC_Os03g05500	LOC_Os08g26990	LOC_Os03g02690	LOC_Os08g39500			LOC_Os08g40630	LOC_Os07g09060
LOC_Os03g05510	LOC_Os04g36070	LOC_Os03g03034	LOC_Os06g21480			LOC_Os07g22670	LOC_Os07g09120
LOC_Os03g05760	LOC_Os07g26720	LOC_Os03g03370	LOC_Os09g32500			LOC_Os01g61320	LOC_Os07g49520
LOC_Os03g06630	LOC_Os01g72330	LOC_Os03g03450	LOC_Os09g32520			LOC_Os02g10260	LOC_Os07g09890
LOC_Os03g06860	LOC_Os08g28900	LOC_Os03g03470	LOC_Os09g32532			LOC_Os12g31450	LOC_Os08g17650
LOC_Os03g07360	LOC_Os08g28950	LOC_Os03g03660	LOC_Os08g06040			LOC_Os03g16440	LOC_Os08g42410
LOC_Os03g07540	LOC_Os11g04720	LOC_Os03g04080	LOC_Os08g33920			LOC_Os04g53210	LOC_Os02g01500
LOC_Os03g07880	LOC_Os04g57720	LOC_Os03g04480	LOC_Os09g24690			LOC_Os07g42440	LOC_Os08g19850
LOC_Os03g08370	LOC_Os02g35180	LOC_Os03g04620	LOC_Os02g30050			LOC_Os08g36140	LOC_Os07g07310
LOC_Os03g08930	LOC_Os02g42060	LOC_Os03g04760	LOC_Os02g54470			LOC_Os03g57220	LOC_Os03g36780
LOC_Os03g09100	LOC_Os02g58350	LOC_Os03g04770	LOC_Os05g48220			LOC_Os08g06630	LOC_Os06g30460
LOC_Os03g09280	LOC_Os04g44280	LOC_Os03g05500	LOC_Os05g48310			LOC_Os04g53214	LOC_Os08g41830
LOC_Os03g10770	LOC_Os12g04500	LOC_Os03g06230	LOC_Os05g38520			LOC_Os02g47360	LOC_Os02g18090
LOC_Os03g11600	LOC_Os05g33730	LOC_Os03g06630	LOC_Os02g56990			LOC_Os07g05820	LOC_Os09g07120
LOC_Os03g12120	LOC_Os03g49990	LOC_Os03g06840	LOC_Os02g02130			LOC_Os03g64020	LOC_Os02g41470
LOC_Os03g12370	LOC_Os01g45860	LOC_Os03g07180	LOC_Os08g03450			LOC_Os08g25720	LOC_Os01g62500
LOC_Os03g12490	LOC_Os03g43810	LOC_Os03g08580	LOC_Os01g48770			LOC_Os03g52660	LOC_Os03g13050
LOC_Os03g12760	LOC_Os03g56950	LOC_Os03g08920	LOC_Os05g48320			LOC_Os06g22060	LOC_Os02g40460
LOC_Os03g12940	LOC_Os12g41650	LOC_Os03g09170	LOC_Os11g24610			LOC_Os03g19380	LOC_Os02g26660
LOC_Os03g13614	LOC_Os01g64730	LOC_Os03g09250	LOC_Os06g08320			LOC_Os03g53640	LOC_Os09g19734
LOC_Os03g14270	LOC_Os08g36790	LOC_Os03g10050	LOC_Os02g55370			LOC_Os08g41460	LOC_Os09g04670
LOC_Os03g14669	LOC_Os09g28310	LOC_Os03g11370	LOC_Os09g27930			LOC_Os04g44200	LOC_Os01g66180
LOC_Os03g15010	LOC_Os05g36160	LOC_Os03g11690	LOC_Os07g30640			LOC_Os02g48360	LOC_Os01g71180
LOC_Os03g15040	LOC_Os06g10880	LOC_Os03g11910	LOC_Os07g30990			LOC_Os08g45190	LOC_Os01g37510
LOC_Os03g15440	LOC_Os02g52780	LOC_Os03g12414	LOC_Os07g31110			LOC_Os06g13810	LOC_Os10g38170
LOC_Os03g15680	LOC_Os05g41070	LOC_Os03g12510	LOC_Os03g13170			LOC_Os01g19740	LOC_Os10g35940
LOC_Os03g16850	LOC_Os07g48660	LOC_Os03g12530	LOC_Os07g26740			LOC_Os01g25600	LOC_Os12g08260
LOC_Os03g17130	LOC_Os01g64000	LOC_Os03g12820	LOC_Os07g33860			LOC_Os02g42000	LOC_Os11g39670
LOC_Os03g17810	LOC_Os03g20650	LOC_Os03g12840	LOC_Os07g33898			LOC_Os10g38216	LOC_Os06g50000
LOC_Os03g18330	LOC_Os01g59760	LOC_Os03g12890	LOC_Os08g03640			LOC_Os10g32300	LOC_Os06g50970
LOC_Os03g18340	LOC_Os01g52050	LOC_Os03g12950	LOC_Os11g04070			LOC_Os10g21406	LOC_Os01g32790
LOC_Os03g19370	LOC_Os08g36970	LOC_Os03g13050	LOC_Os12g03880			LOC_Os08g21900	LOC_Os01g60110
LOC_Os03g19375	LOC_Os09g28550	LOC_Os03g13140	LOC_Os06g48780			LOC_Os06g50070	LOC_Os01g14830
LOC_Os03g20310	LOC_Os03g04050	LOC_Os03g13200	LOC_Os01g13080			LOC_Os11g16550	LOC_Os01g57930
LOC_Os03g20550	LOC_Os10g42110	LOC_Os03g13740	LOC_Os08g02340			LOC_Os02g02400	LOC_Os05g05560
LOC_Os03g20650	LOC_Os02g14130	LOC_Os03g14010	LOC_Os07g28710			LOC_Os06g51150	LOC_Os05g05320
LOC_Os03g20780	LOC_Os01g10840	LOC_Os03g14030	LOC_Os08g15180			LOC_Os08g29669	LOC_Os03g48110
LOC_Os03g20790	LOC_Os05g11730	LOC_Os03g14080	LOC_Os01g09510			LOC_Os08g41830	LOC_Os01g53620
LOC_Os03g21030	LOC_Os06g35530	LOC_Os03g14120	LOC_Os07g14750			LOC_Os02g43360	LOC_Os01g74520
LOC_Os03g21060	LOC_Os07g39220	LOC_Os03g14130	LOC_Os05g37330			LOC_Os05g49040	LOC_Os01g53140
LOC_Os03g21660	LOC_Os04g51460	LOC_Os03g14950	LOC_Os02g32760			LOC_Os01g73990	LOC_Os01g73020
LOC_Os03g21710	LOC_Os06g48160	LOC_Os03g14990	LOC_Os03g21530			LOC_Os12g25630	LOC_Os01g70800
LOC_Os03g21800	LOC_Os06g48180	LOC_Os03g15033	LOC_Os12g07300			LOC_Os12g12170	LOC_Os05g39390
LOC_Os03g25120	LOC_Os09g02360	LOC_Os03g15320	LOC_Os03g61790			LOC_Os05g01820	LOC_Os05g49300
LOC_Os03g25430	LOC_Os05g50890	LOC_Os03g15460	LOC_Os11g45250			LOC_Os03g03910	LOC_Os05g09500
LOC_Os03g26210	LOC_Os03g15880	LOC_Os03g15920	LOC_Os05g39530			LOC_Os10g37420	LOC_Os01g09460
LOC_Os03g27390	LOC_Os01g63420	LOC_Os03g15960	LOC_Os04g22870			LOC_Os09g16520	LOC_Os06g23210
LOC_Os03g29480	LOC_Os05g37690	LOC_Os03g16030	LOC_Os05g18780			LOC_Os03g48170	LOC_Os06g49890
LOC_Os03g29760	LOC_Os08g33160	LOC_Os03g16470	LOC_Os04g34940			LOC_Os12g08730	LOC_Os01g56310
LOC_Os03g29970	LOC_Os04g55920	LOC_Os03g16900	LOC_Os11g01074			LOC_Os03g31150	LOC_Os06g19640
LOC_Os03g31880	LOC_Os04g32480	LOC_Os03g16920	LOC_Os12g01060			LOC_Os01g03750	LOC_Os01g22900
LOC_Os03g33012	LOC_Os03g28940	LOC_Os03g16980	LOC_Os12g07680			LOC_Os08g07840	LOC_Os01g45530
LOC_Os03g33090	LOC_Os07g42370	LOC_Os03g17060	LOC_Os12g08740			LOC_Os03g07140	LOC_Os01g08930
LOC_Os03g37900	LOC_Os09g23650	LOC_Os03g17590	LOC_Os04g55700			LOC_Os08g01170	LOC_Os05g01110
LOC_Os03g37920	LOC_Os03g08310	LOC_Os03g17790	LOC_Os02g26660			LOC_Os10g01044	LOC_Os05g46290
LOC_Os03g38870	LOC_Os03g08320	LOC_Os03g17800	LOC_Os04g23830			LOC_Os05g06890	LOC_Os05g35540
LOC_Os03g39050	LOC_Os03g08330	LOC_Os03g17810	LOC_Os01g65220			LOC_Os07g46410	LOC_Os06g29220
LOC_Os03g39100	LOC_Os09g26780	LOC_Os03g18030	LOC_Os08g06230			LOC_Os05g40260	LOC_Os06g42770
LOC_Os03g40080	LOC_Os10g25230	LOC_Os03g18530	LOC_Os07g01920			LOC_Os06g51290	LOC_Os06g09679
LOC_Os03g40160	LOC_Os10g25290	LOC_Os03g18850	LOC_Os03g22890			LOC_Os09g38420	LOC_Os06g09698
LOC_Os03g41330	LOC_Os01g56200	LOC_Os03g19220	LOC_Os01g27730			LOC_Os02g58550	LOC_Os01g59490
LOC_Os03g41600	LOC_Os01g72020	LOC_Os03g19270	LOC_Os06g01980			LOC_Os12g43130	LOC_Os06g05250
LOC_Os03g41800	LOC_Os11g04600	LOC_Os03g19427	LOC_Os10g31856			LOC_Os12g35630	LOC_Os01g46380
LOC_Os03g42100	LOC_Os01g09800	LOC_Os03g19980	LOC_Os03g60930			LOC_Os11g37990	LOC_Os06g04380
LOC_Os03g42200	LOC_Os03g46440	LOC_Os03g22010	LOC_Os03g58720			LOC_Os12g08220	LOC_Os01g45070
LOC_Os03g42630	LOC_Os12g04410	LOC_Os03g22200	LOC_Os02g02360			LOC_Os01g01340	LOC_Os06g05670
LOC_Os03g43730	LOC_Os01g59350	LOC_Os03g22330	LOC_Os05g49890			LOC_Os04g54800	LOC_Os09g16910
LOC_Os03g43810	LOC_Os05g41280	LOC_Os03g22590	LOC_Os06g39875			LOC_Os06g27760	LOC_Os02g33500
LOC_Os03g44540	LOC_Os08g07970	LOC_Os03g22790	LOC_Os03g64080			LOC_Os01g67430	LOC_Os05g49880
LOC_Os03g44710	LOC_Os01g17260	LOC_Os03g22800	LOC_Os10g42320			LOC_Os02g51410	LOC_Os02g33070
LOC_Os03g45300	LOC_Os04g54474	LOC_Os03g22820	LOC_Os01g17330			LOC_Os06g12150	LOC_Os09g11250
LOC_Os03g45450	LOC_Os09g10840	LOC_Os03g24020	LOC_Os07g44620			LOC_Os03g31300	LOC_Os02g37000
LOC_Os03g45750	LOC_Os12g05680	LOC_Os03g24380	LOC_Os08g01620			LOC_Os10g41400	LOC_Os12g34860
LOC_Os03g46350	LOC_Os06g15480	LOC_Os03g24510	LOC_Os02g33850			LOC_Os02g32730	LOC_Os11g43610
LOC_Os03g46790	LOC_Os06g41100	LOC_Os03g25460	LOC_Os03g44780			LOC_Os02g15750	LOC_Os12g37640
LOC_Os03g46860	LOC_Os02g10140	LOC_Os03g26210	LOC_Os03g44530			LOC_Os01g54270	LOC_Os12g38650
LOC_Os03g47200	LOC_Os03g20310	LOC_Os03g26430	LOC_Os12g42370			LOC_Os03g08570	LOC_Os12g40450
LOC_Os03g47970	LOC_Os05g37170	LOC_Os03g26820	LOC_Os07g22950			LOC_Os05g50550	LOC_Os12g41250
LOC_Os03g48450	LOC_Os11g05480	LOC_Os03g26990	LOC_Os07g06820			LOC_Os11g37650	LOC_Os12g42180
LOC_Os03g48970	LOC_Os07g48820	LOC_Os03g27019	LOC_Os01g66520			LOC_Os07g49390	LOC_Os12g44080
LOC_Os03g49880	LOC_Os09g31390	LOC_Os03g27080	LOC_Os12g35630			LOC_Os04g34460	LOC_Os07g28470
LOC_Os03g49990	LOC_Os01g64020	LOC_Os03g30830	LOC_Os02g21660			LOC_Os03g03370	LOC_Os09g26730
LOC_Os03g50110	LOC_Os06g42680	LOC_Os03g30950	LOC_Os04g33750			LOC_Os03g58100	LOC_Os07g48960
LOC_Os03g50900	LOC_Os05g02730	LOC_Os03g31360	LOC_Os12g34128			LOC_Os05g44350	LOC_Os07g22700
LOC_Os03g50920	LOC_Os06g44750	LOC_Os03g32490	LOC_Os04g42200			LOC_Os01g73780	LOC_Os09g12560
LOC_Os03g51330	LOC_Os08g42580	LOC_Os03g36910	LOC_Os09g24412			LOC_Os04g22360	LOC_Os01g09560
LOC_Os03g51580	LOC_Os05g51830	LOC_Os03g38540	LOC_Os10g21196			LOC_Os04g04230	LOC_Os01g47420
LOC_Os03g51910	LOC_Os06g50310	LOC_Os03g39760	LOC_Os10g21374			LOC_Os04g57500	LOC_Os06g30380
LOC_Os03g52450	LOC_Os01g45990	LOC_Os03g40100	LOC_Os10g21314			LOC_Os04g35180	LOC_Os06g45670
LOC_Os03g53020	LOC_Os03g04110	LOC_Os03g41060	LOC_Os03g63950			LOC_Os04g39450	LOC_Os01g21160
LOC_Os03g53050	LOC_Os02g09060	LOC_Os03g41330	LOC_Os03g01910			LOC_Os03g58300	LOC_Os06g47000
LOC_Os03g53340	LOC_Os01g54930	LOC_Os03g43720	LOC_Os03g08440			LOC_Os03g58260	LOC_Os05g06330
LOC_Os03g55080	LOC_Os11g01074	LOC_Os03g43820	LOC_Os03g63400			LOC_Os03g55820	LOC_Os06g03770
LOC_Os03g55164	LOC_Os05g43950	LOC_Os03g44380	LOC_Os03g16874			LOC_Os03g45400	LOC_Os06g03790
LOC_Os03g55220	LOC_Os01g03340	LOC_Os03g45390	LOC_Os07g08660			LOC_Os04g55690	LOC_Os01g01610
LOC_Os03g55550	LOC_Os10g34540	LOC_Os03g45720	LOC_Os04g30780			LOC_Os04g55720	LOC_Os02g18810
LOC_Os03g55610	LOC_Os05g06270	LOC_Os03g45750	LOC_Os04g30730			LOC_Os04g44560	LOC_Os02g17370
LOC_Os03g56010	LOC_Os06g49080	LOC_Os03g45960	LOC_Os07g10300			LOC_Os04g51330	LOC_Os02g54060
LOC_Os03g56580	LOC_Os03g05510	LOC_Os03g46770	LOC_Os12g37610			LOC_Os04g55180	LOC_Os02g57430
LOC_Os03g56630	LOC_Os03g12660	LOC_Os03g46860	LOC_Os09g08890			LOC_Os10g42240	LOC_Os02g57180
LOC_Os03g56950	LOC_Os04g39430	LOC_Os03g47610	LOC_Os03g10930			LOC_Os08g35620	LOC_Os06g29180
LOC_Os03g57190	LOC_Os11g04710	LOC_Os03g48000	LOC_Os08g40230			LOC_Os07g38150	LOC_Os01g72980
LOC_Os03g57670	LOC_Os12g04480	LOC_Os03g48010	LOC_Os02g55390			LOC_Os07g26610	LOC_Os05g05840
LOC_Os03g58160	LOC_Os01g63260	LOC_Os03g48030	LOC_Os02g18090			LOC_Os10g30870	LOC_Os06g05080
LOC_Os03g58250	LOC_Os11g08110	LOC_Os03g48050	LOC_Os12g34144			LOC_Os01g52500	LOC_Os09g23070
LOC_Os03g58330	LOC_Os03g40540	LOC_Os03g48060	LOC_Os01g32790			LOC_Os01g14820	LOC_Os02g10260
LOC_Os03g58420	LOC_Os01g10040	LOC_Os03g49430	LOC_Os06g19640			LOC_Os04g33390	LOC_Os02g43720
LOC_Os03g58830	LOC_Os09g26960	LOC_Os03g49480	LOC_Os10g21358			LOC_Os09g33810	LOC_Os02g07410
LOC_Os03g59460	LOC_Os09g26940	LOC_Os03g49630	LOC_Os06g03790			LOC_Os07g05940	LOC_Os01g34390
LOC_Os03g59670	LOC_Os08g35510	LOC_Os03g50780	LOC_Os09g24404			LOC_Os09g36040	LOC_Os01g61380
LOC_Os03g59730	LOC_Os09g26980	LOC_Os03g51210	LOC_Os09g10770			LOC_Os09g24320	LOC_Os06g50870
LOC_Os03g60080	LOC_Os02g11020	LOC_Os03g51530	LOC_Os12g14080			LOC_Os04g54640	LOC_Os06g47940
LOC_Os03g60630	LOC_Os06g39880	LOC_Os03g51760	LOC_Os10g34180			LOC_Os07g01030	LOC_Os06g47950
LOC_Os03g61249	LOC_Os07g45290	LOC_Os03g51920	LOC_Os09g24414			LOC_Os05g09440	LOC_Os06g46930
LOC_Os03g61319	LOC_Os03g24440	LOC_Os03g52340	LOC_Os05g22724			LOC_Os04g41580	LOC_Os01g42980
LOC_Os03g61570	LOC_Os01g49390	LOC_Os03g52390	LOC_Os10g21342			LOC_Os04g43690	LOC_Os01g57073
LOC_Os03g61650	LOC_Os05g24660	LOC_Os03g52680	LOC_Os08g15278			LOC_Os04g51300	LOC_Os01g06454
LOC_Os03g61760	LOC_Os07g09220	LOC_Os03g53340	LOC_Os10g21372			LOC_Os04g14680	LOC_Os01g40620
LOC_Os03g62470	LOC_Os03g24240	LOC_Os03g53400	LOC_Os10g38214			LOC_Os04g09900	LOC_Os12g33240
LOC_Os03g62660	LOC_Os05g47840	LOC_Os03g53620	LOC_Os09g24416			LOC_Os04g39190	LOC_Os01g57460
LOC_Os03g63530	LOC_Os07g11050	LOC_Os03g53860	LOC_Os10g38278			LOC_Os02g32930	LOC_Os02g07044
LOC_Os03g63750	LOC_Os03g59570	LOC_Os03g53900	LOC_Os08g15308			LOC_Os10g26070	LOC_Os09g20660
LOC_Os03g63810	LOC_Os06g37500	LOC_Os03g53930	LOC_Os10g38238			LOC_Os10g21266	LOC_Os09g20430
LOC_Os04g02140	LOC_Os05g31040	LOC_Os03g54000	LOC_Os10g21332			LOC_Os06g33520	LOC_Os09g20440
LOC_Os04g04300	LOC_Os02g12780	LOC_Os03g54040	LOC_Os10g35690			LOC_Os01g38580	LOC_Os12g33160
LOC_Os04g04400	LOC_Os01g10110	LOC_Os03g55030	LOC_Os02g11030			LOC_Os10g42100	LOC_Os05g45950
LOC_Os04g09560	LOC_Os01g56810	LOC_Os03g55220	LOC_Os12g34092			LOC_Os06g06190	LOC_Os05g43360
LOC_Os04g10260	LOC_Os01g71310	LOC_Os03g55590	LOC_Os07g33997			LOC_Os06g29430	LOC_Os05g43440
LOC_Os04g10860	LOC_Os04g44230	LOC_Os03g56000	LOC_Os12g30490			LOC_Os06g43800	LOC_Os05g43470
LOC_Os04g11830	LOC_Os10g34230	LOC_Os03g56060	LOC_Os12g23620			LOC_Os02g24020	LOC_Os05g43576
LOC_Os04g21950	LOC_Os01g09260	LOC_Os03g56260	LOC_Os10g20910			LOC_Os09g28100	LOC_Os05g29860
LOC_Os04g22990	LOC_Os03g24430	LOC_Os03g56820	LOC_Os08g41300			LOC_Os09g27050	LOC_Os05g47840
LOC_Os04g23440	LOC_Os10g09990	LOC_Os03g56930	LOC_Os02g39350			LOC_Os09g27080	LOC_Os07g12150
LOC_Os04g23550	LOC_Os01g73760	LOC_Os03g56940	LOC_Os02g48660			LOC_Os06g05110	LOC_Os05g45590
LOC_Os04g25100	LOC_Os04g46980	LOC_Os03g56950	LOC_Os06g35470			LOC_Os06g02500	LOC_Os01g52450
LOC_Os04g28280	LOC_Os04g46970	LOC_Os03g57660	LOC_Os07g43510			LOC_Os06g01400	LOC_Os05g31110
LOC_Os04g28580	LOC_Os04g46990	LOC_Os03g57670	LOC_Os06g51530			LOC_Os06g12510	LOC_Os01g71320
LOC_Os04g30870	LOC_Os04g47720	LOC_Os03g58420	LOC_Os07g23800			LOC_Os06g12370	LOC_Os02g57670
LOC_Os04g31290	LOC_Os04g47770	LOC_Os03g58790				LOC_Os10g36860	LOC_Os03g07330
LOC_Os04g31900	LOC_Os07g46610	LOC_Os03g59320				LOC_Os04g43350	LOC_Os02g40830
LOC_Os04g32590	LOC_Os05g41270	LOC_Os03g60080				LOC_Os10g35140	LOC_Os05g08410
LOC_Os04g33420	LOC_Os02g58520	LOC_Os03g60250				LOC_Os02g57100	LOC_Os01g16900
LOC_Os04g33440	LOC_Os01g43410	LOC_Os03g60260				LOC_Os03g37090	LOC_Os01g16152
LOC_Os04g33950	LOC_Os02g46090	LOC_Os03g60300				LOC_Os03g25960	LOC_Os01g53700
LOC_Os04g35010	LOC_Os04g47300	LOC_Os03g60580				LOC_Os12g07700	LOC_Os03g62420
LOC_Os04g35250	LOC_Os07g33110	LOC_Os03g60840				LOC_Os10g10244	LOC_Os03g59590
LOC_Os04g35500	LOC_Os10g39420	LOC_Os03g61150				LOC_Os03g16470	LOC_Os03g59570
LOC_Os04g35660	LOC_Os07g38120	LOC_Os03g61260				LOC_Os03g18020	LOC_Os02g55310
LOC_Os04g36590	LOC_Os03g57450	LOC_Os03g61670				LOC_Os03g17690	LOC_Os01g31610
LOC_Os04g36790	LOC_Os05g39090	LOC_Os03g62330				LOC_Os03g19290	LOC_Os01g70960
LOC_Os04g37440	LOC_Os07g22710	LOC_Os03g62610				LOC_Os03g19560	LOC_Os02g43470
LOC_Os04g38400	LOC_Os04g49510	LOC_Os03g63330				LOC_Os03g20700	LOC_Os02g32120
LOC_Os04g38720	LOC_Os05g41090	LOC_Os03g63620				LOC_Os02g30200	LOC_Os01g52214
LOC_Os04g39570	LOC_Os11g04170	LOC_Os03g63810				LOC_Os07g19030	LOC_Os01g66330
LOC_Os04g39960	LOC_Os07g06740	LOC_Os03g64320				LOC_Os07g36390	LOC_Os01g49330
LOC_Os04g40060	LOC_Os01g59360	LOC_Os04g01910				LOC_Os08g19610	LOC_Os02g44000
LOC_Os04g40140	LOC_Os03g48270	LOC_Os04g02620				LOC_Os03g05806	LOC_Os05g40300
LOC_Os04g40930	LOC_Os01g61590	LOC_Os04g02880				LOC_Os03g09870	LOC_Os01g12560
LOC_Os04g41229	LOC_Os05g50810	LOC_Os04g06590				LOC_Os03g11700	LOC_Os01g12570
LOC_Os04g41820	LOC_Os11g07040	LOC_Os04g07110				LOC_Os03g11120	LOC_Os05g15360
LOC_Os04g41850	LOC_Os12g30150	LOC_Os04g09260				LOC_Os03g11560	LOC_Os05g15370
LOC_Os04g42940	LOC_Os02g03410	LOC_Os04g09390				LOC_Os03g15840	LOC_Os01g22520
LOC_Os04g43560	LOC_Os03g59390	LOC_Os04g09900				LOC_Os03g60740	LOC_Os02g05870
LOC_Os04g44440	LOC_Os08g42750	LOC_Os04g10010				LOC_Os03g14120	LOC_Os05g49760
LOC_Os04g44630	LOC_Os09g33910	LOC_Os04g10060				LOC_Os03g63360	LOC_Os05g49800
LOC_Os04g45330	LOC_Os12g07230	LOC_Os04g10160				LOC_Os03g63480	LOC_Os05g38330
LOC_Os04g45650	LOC_Os03g03660	LOC_Os04g11040				LOC_Os03g39830	LOC_Os05g45270
LOC_Os04g45750	LOC_Os03g57510	LOC_Os04g12639				LOC_Os03g48920	LOC_Os05g01750
LOC_Os04g45940	LOC_Os12g03970	LOC_Os04g12720				LOC_Os07g37250	LOC_Os01g68030
LOC_Os04g46020	LOC_Os06g33570	LOC_Os04g14680				LOC_Os06g06560	LOC_Os01g52720
LOC_Os04g46060	LOC_Os02g15580	LOC_Os04g14810				LOC_Os02g49870	LOC_Os01g73760
LOC_Os04g46580	LOC_Os03g44440	LOC_Os04g16680				LOC_Os02g57160	LOC_Os01g72049
LOC_Os04g46860	LOC_Os06g10580	LOC_Os04g20330				LOC_Os02g57290	LOC_Os01g72280
LOC_Os04g47040	LOC_Os02g54760	LOC_Os04g23910				LOC_Os06g02540	LOC_Os01g19490
LOC_Os04g47059	LOC_Os02g41710	LOC_Os04g25380				LOC_Os12g43630	LOC_Os01g19529
LOC_Os04g47080	LOC_Os03g55100	LOC_Os04g25650				LOC_Os03g30950	LOC_Os01g73420
LOC_Os04g47640	LOC_Os09g38580	LOC_Os04g27060				LOC_Os04g01540	LOC_Os01g73550
LOC_Os04g47990	LOC_Os02g53340	LOC_Os04g28420				LOC_Os03g16140	LOC_Os05g45730
LOC_Os04g48030	LOC_Os12g28260	LOC_Os04g28620				LOC_Os03g17730	LOC_Os04g59494
LOC_Os04g49110	LOC_Os05g42250	LOC_Os04g29680				LOC_Os03g58620	LOC_Os04g57320
LOC_Os04g49380	LOC_Os11g04480	LOC_Os04g30450				LOC_Os03g11510	LOC_Os03g24240
LOC_Os04g50060	LOC_Os07g48340	LOC_Os04g31120				LOC_Os03g12236	LOC_Os11g01270
LOC_Os04g50090	LOC_Os01g72080	LOC_Os04g31410				LOC_Os03g11670	LOC_Os04g51150
LOC_Os04g50920	LOC_Os05g50180	LOC_Os04g31610				LOC_Os03g58710	LOC_Os04g51160
LOC_Os04g51000	LOC_Os12g12730	LOC_Os04g32480				LOC_Os01g01302	LOC_Os12g26060
LOC_Os04g51070	LOC_Os04g41540	LOC_Os04g33670				LOC_Os02g36340	LOC_Os11g29390
LOC_Os04g51320	LOC_Os08g44390	LOC_Os04g33920				LOC_Os05g33840	LOC_Os12g25630
LOC_Os04g51560	LOC_Os05g05460	LOC_Os04g34010				LOC_Os03g60910	LOC_Os11g32480
LOC_Os04g52560	LOC_Os08g44660	LOC_Os04g34370				LOC_Os03g63330	LOC_Os11g34140
LOC_Os04g52770	LOC_Os12g04360	LOC_Os04g35100				LOC_Os04g31070	LOC_Os11g34210
LOC_Os04g52810	LOC_Os11g01390	LOC_Os04g35660				LOC_Os07g13590	LOC_Os12g29990
LOC_Os04g53990	LOC_Os01g32120	LOC_Os04g36062				LOC_Os08g29160	LOC_Os12g30030
LOC_Os04g54474	LOC_Os01g41990	LOC_Os04g36720				LOC_Os04g33940	LOC_Os11g38030
LOC_Os04g54880	LOC_Os05g13580	LOC_Os04g36750				LOC_Os04g46460	LOC_Os11g37330
LOC_Os04g54900	LOC_Os01g72100	LOC_Os04g36800				LOC_Os10g43060	LOC_Os12g34340
LOC_Os04g56170	LOC_Os01g04330	LOC_Os04g37710				LOC_Os03g02690	LOC_Os12g01240
LOC_Os04g57530	LOC_Os05g31620	LOC_Os04g37790				LOC_Os03g55640	LOC_Os12g01500
LOC_Os04g58190	LOC_Os03g55960	LOC_Os04g38560				LOC_Os03g58320	LOC_Os11g47800
LOC_Os04g58680	LOC_Os01g56030	LOC_Os04g38690				LOC_Os03g15033	LOC_Os12g02040
LOC_Os04g59470	LOC_Os01g72550	LOC_Os04g39110				LOC_Os03g20100	LOC_Os12g02880
LOC_Os05g01256	LOC_Os01g72540	LOC_Os04g39570				LOC_Os02g04460	LOC_Os10g23120
LOC_Os05g02150	LOC_Os01g72530	LOC_Os04g40490				LOC_Os03g31490	LOC_Os11g11020
LOC_Os05g03090	LOC_Os12g04240	LOC_Os04g40520				LOC_Os07g07060	LOC_Os12g07260
LOC_Os05g03160	LOC_Os02g10470	LOC_Os04g40560				LOC_Os07g10490	LOC_Os12g07650
LOC_Os05g03740	LOC_Os06g40720	LOC_Os04g41550				LOC_Os07g08160	LOC_Os04g45970
LOC_Os05g03800	LOC_Os08g02420	LOC_Os04g41620				LOC_Os07g08340	LOC_Os10g33910
LOC_Os05g03860	LOC_Os08g04890	LOC_Os04g41680				LOC_Os06g37300	LOC_Os10g35960
LOC_Os05g03865	LOC_Os03g21380	LOC_Os04g41740				LOC_Os05g03830	LOC_Os10g37210
LOC_Os05g03900	LOC_Os09g28480	LOC_Os04g42720				LOC_Os07g38390	LOC_Os10g40360
LOC_Os05g04640	LOC_Os10g25010	LOC_Os04g43070				LOC_Os05g05950	LOC_Os12g14080
LOC_Os05g04740	LOC_Os05g24780	LOC_Os04g43200				LOC_Os05g22730	LOC_Os08g28230
LOC_Os05g06170	LOC_Os11g04560	LOC_Os04g43820				LOC_Os05g34470	LOC_Os08g02640
LOC_Os05g06340	LOC_Os12g01400	LOC_Os04g44060				LOC_Os05g38400	LOC_Os03g56190
LOC_Os05g06520	LOC_Os12g41110	LOC_Os04g44280				LOC_Os01g43130	LOC_Os02g13840
LOC_Os05g07120	LOC_Os02g01440	LOC_Os04g45480				LOC_Os09g29840	LOC_Os02g33730
LOC_Os05g07270	LOC_Os02g38210	LOC_Os04g45940				LOC_Os09g32820	LOC_Os02g44570
LOC_Os05g09020	LOC_Os03g63410	LOC_Os04g46000				LOC_Os12g18630	LOC_Os07g38300
LOC_Os05g10620	LOC_Os01g32660	LOC_Os04g46130				LOC_Os12g32986	LOC_Os02g14110
LOC_Os05g11070	LOC_Os04g55410	LOC_Os04g47260				LOC_Os08g15500	LOC_Os08g30884
LOC_Os05g14370	LOC_Os01g54890	LOC_Os04g48030				LOC_Os08g14440	LOC_Os07g38970
LOC_Os05g23910	LOC_Os05g04520	LOC_Os04g48390				LOC_Os11g07916	LOC_Os08g02080
LOC_Os05g25320	LOC_Os09g33860	LOC_Os04g48880				LOC_Os08g31750	LOC_Os05g22724
LOC_Os05g25770	LOC_Os03g62700	LOC_Os04g49194				LOC_Os09g10740	LOC_Os10g21342
LOC_Os05g25960	LOC_Os01g21970	LOC_Os04g49210				LOC_Os01g73540	LOC_Os01g58000
LOC_Os05g26026	LOC_Os01g67340	LOC_Os04g49370				LOC_Os04g48370	LOC_Os06g46435
LOC_Os05g26049	LOC_Os08g41470	LOC_Os04g49450				LOC_Os01g47080	LOC_Os07g44440

LOC_Os05g27730	LOC_Os03g63140	LOC_Os04g49940
LOC_Os05g27749	LOC_Os04g31030	LOC_Os04g50940
LOC_Os05g27980	LOC_Os02g30190	LOC_Os04g51160
LOC_Os05g30310	LOC_Os07g08890	LOC_Os04g51460
LOC_Os05g30720	LOC_Os08g16070	LOC_Os04g51520
LOC_Os05g31380	LOC_Os11g12000	LOC_Os04g51660
LOC_Os05g31420	LOC_Os08g09430	LOC_Os04g51890
LOC_Os05g32070	LOC_Os06g22460	LOC_Os04g52090
LOC_Os05g33810	LOC_Os04g02860	LOC_Os04g52164
LOC_Os05g34050	LOC_Os07g09940	LOC_Os04g52310
LOC_Os05g34310	LOC_Os08g16120	LOC_Os04g52504
LOC_Os05g34450	LOC_Os04g14220	LOC_Os04g52780
LOC_Os05g34600	LOC_Os10g07574	LOC_Os04g54230
LOC_Os05g34830	LOC_Os10g07978	LOC_Os04g55250
LOC_Os05g35170	LOC_Os12g14330	LOC_Os04g55600
LOC_Os05g36160	LOC_Os11g45330	LOC_Os04g56130
LOC_Os05g36900	LOC_Os10g04674	LOC_Os04g56180
LOC_Os05g37080	LOC_Os11g11960	LOC_Os04g56250
LOC_Os05g37170	LOC_Os11g11990	LOC_Os04g56400
LOC_Os05g37540	LOC_Os11g12300	LOC_Os04g56430
LOC_Os05g38140	LOC_Os09g30220	LOC_Os04g56450
LOC_Os05g38820	LOC_Os08g32880	LOC_Os04g56680
LOC_Os05g39480	LOC_Os09g09750	LOC_Os04g56710
LOC_Os05g39720	LOC_Os01g36640	LOC_Os04g56760
LOC_Os05g39790	LOC_Os12g31620	LOC_Os04g56920
LOC_Os05g40060	LOC_Os02g33180	LOC_Os04g57310
LOC_Os05g40070	LOC_Os01g43540	LOC_Os04g57739
LOC_Os05g40080	LOC_Os01g32930	LOC_Os04g57880
LOC_Os05g40680	LOC_Os08g38086	LOC_Os04g58120
LOC_Os05g40710	LOC_Os09g29840	LOC_Os04g58140
LOC_Os05g41070	LOC_Os06g50300	LOC_Os04g58330
LOC_Os05g41280	LOC_Os12g32986	LOC_Os04g58420
LOC_Os05g41450	LOC_Os09g30412	LOC_Os04g59210
LOC_Os05g41540	LOC_Os09g30418	LOC_Os04g59440
LOC_Os05g42130	LOC_Os08g39140	LOC_Os04g59540
LOC_Os05g42180	LOC_Os01g57870	LOC_Os05g01140
LOC_Os05g43380	LOC_Os01g72680	LOC_Os05g01270
LOC_Os05g43760	LOC_Os04g39460	LOC_Os05g01990
LOC_Os05g43950	LOC_Os02g30900	LOC_Os05g03120
LOC_Os05g43960	LOC_Os04g32310	LOC_Os05g04450
LOC_Os05g44400	LOC_Os09g22450	LOC_Os05g04610
LOC_Os05g45230	LOC_Os07g29760	LOC_Os05g04660
LOC_Os05g45410	LOC_Os02g27030	LOC_Os05g05290
LOC_Os05g46020	LOC_Os04g24600	LOC_Os05g05640
LOC_Os05g46370	LOC_Os03g12030	LOC_Os05g06920
LOC_Os05g48690	LOC_Os06g14810	LOC_Os05g06970
LOC_Os05g48850	LOC_Os06g15170	LOC_Os05g07060
LOC_Os05g49100	LOC_Os03g06700	LOC_Os05g08480
LOC_Os05g49210	LOC_Os03g06705	LOC_Os05g09440
LOC_Os05g49280	LOC_Os04g02640	LOC_Os05g10210
LOC_Os05g49420	LOC_Os03g08360	LOC_Os05g10310
LOC_Os05g49620	LOC_Os03g14170	LOC_Os05g10370
LOC_Os05g49780	LOC_Os09g19650	LOC_Os05g10800
LOC_Os05g49930	LOC_Os09g34930	LOC_Os05g10830
LOC_Os05g50270	LOC_Os10g33370	LOC_Os05g11060
LOC_Os05g50310	LOC_Os11g37900	LOC_Os05g11250
LOC_Os05g50610	LOC_Os05g49290	LOC_Os05g12460
LOC_Os05g50700	LOC_Os05g49900	LOC_Os05g13320
LOC_Os05g50900	LOC_Os06g39750	LOC_Os05g13940
LOC_Os05g51040	LOC_Os02g49920	LOC_Os05g14270
LOC_Os05g51820	LOC_Os03g26620	LOC_Os05g15530
LOC_Os06g01230	LOC_Os10g28060	LOC_Os05g15690
LOC_Os06g01480	LOC_Os01g34560	LOC_Os05g15770
LOC_Os06g01620	LOC_Os02g11070	LOC_Os05g15920
LOC_Os06g03710	LOC_Os02g56860	LOC_Os05g19500
LOC_Os06g04010	LOC_Os03g26530	LOC_Os05g19600
LOC_Os06g04090	LOC_Os08g38990	LOC_Os05g20150
LOC_Os06g05350	LOC_Os04g39570	LOC_Os05g23910
LOC_Os06g05380	LOC_Os09g30400	LOC_Os05g24654
LOC_Os06g06360	LOC_Os11g36180	LOC_Os05g25210
LOC_Os06g06900	LOC_Os03g59040	LOC_Os05g25540
LOC_Os06g07010	LOC_Os07g10130	LOC_Os05g25580
LOC_Os06g08500	LOC_Os03g12900	LOC_Os05g25770
LOC_Os06g09370	LOC_Os03g12910	LOC_Os05g27050
LOC_Os06g10710	LOC_Os02g02230	LOC_Os05g27790
LOC_Os06g10820	LOC_Os07g37970	LOC_Os05g29790
LOC_Os06g10880	LOC_Os05g12040	LOC_Os05g30250
LOC_Os06g10900	LOC_Os02g21810	LOC_Os05g31140
LOC_Os06g12210	LOC_Os05g34325	LOC_Os05g31670
LOC_Os06g12230	LOC_Os05g34380	LOC_Os05g32110
LOC_Os06g12360	LOC_Os11g32240	LOC_Os05g32150
LOC_Os06g13670	LOC_Os01g25189	LOC_Os05g33030
LOC_Os06g13740	LOC_Os09g39220	LOC_Os05g33140
LOC_Os06g13900	LOC_Os01g01369	LOC_Os05g33400
LOC_Os06g14190	LOC_Os10g25780	LOC_Os05g33920
LOC_Os06g15480	LOC_Os01g04260	LOC_Os05g33940
LOC_Os06g16400	LOC_Os02g26650	LOC_Os05g34150
LOC_Os06g17410	LOC_Os06g42860	LOC_Os05g34700
LOC_Os06g17480	LOC_Os08g41780	LOC_Os05g34800
LOC_Os06g22670	LOC_Os09g01590	LOC_Os05g35230
LOC_Os06g23030	LOC_Os03g59620	LOC_Os05g36186
LOC_Os06g23650	LOC_Os01g55650	LOC_Os05g36260
LOC_Os06g25610	LOC_Os03g59290	LOC_Os05g37250
LOC_Os06g28150	LOC_Os07g10600	LOC_Os05g37780
LOC_Os06g30090	LOC_Os11g19140	LOC_Os05g37910
LOC_Os06g30860	LOC_Os02g04710	LOC_Os05g37930
LOC_Os06g32530	LOC_Os10g39810	LOC_Os05g37970
LOC_Os06g33450	LOC_Os03g01820	LOC_Os05g38250
LOC_Os06g33940	LOC_Os03g29170	LOC_Os05g38280
LOC_Os06g35900	LOC_Os09g34090	LOC_Os05g38350
LOC_Os06g35960	LOC_Os03g29150	LOC_Os05g38500
LOC_Os06g36480	LOC_Os11g19700	LOC_Os05g38530
LOC_Os06g36930	LOC_Os03g04340	LOC_Os05g38770
LOC_Os06g36970	LOC_Os07g01150	LOC_Os05g38880
LOC_Os06g37410	LOC_Os11g48020	LOC_Os05g39140
LOC_Os06g37450	LOC_Os01g11270	LOC_Os05g39250
LOC_Os06g39090	LOC_Os01g11280	LOC_Os05g39800
LOC_Os06g39680	LOC_Os01g11300	LOC_Os05g40020
LOC_Os06g39960	LOC_Os01g11340	LOC_Os05g41166
LOC_Os06g40780	LOC_Os02g26814	LOC_Os05g41290
LOC_Os06g41100	LOC_Os03g08300	LOC_Os05g41950
LOC_Os06g41770	LOC_Os04g49960	LOC_Os05g42100
LOC_Os06g42140	LOC_Os12g39340	LOC_Os05g42150
LOC_Os06g42420	LOC_Os01g11070	LOC_Os05g43030
LOC_Os06g42520	LOC_Os04g46050	LOC_Os05g43910
LOC_Os06g42690	LOC_Os05g44360	LOC_Os05g43940
LOC_Os06g43870	LOC_Os04g57890	LOC_Os05g44060
LOC_Os06g44010	LOC_Os05g23600	LOC_Os05g44300
LOC_Os06g44860	LOC_Os01g68324	LOC_Os05g44340
LOC_Os06g45140	LOC_Os04g32550	LOC_Os05g44610
LOC_Os06g45310	LOC_Os03g36730	LOC_Os05g45100
LOC_Os06g45640	LOC_Os07g10830	LOC_Os05g45710
LOC_Os06g46270	LOC_Os05g11850	LOC_Os05g45810
LOC_Os06g48534	LOC_Os04g51690	LOC_Os05g45820
LOC_Os06g49010	LOC_Os07g23494	LOC_Os05g46050
LOC_Os06g49550	LOC_Os02g58590	LOC_Os05g46480
LOC_Os06g49830	LOC_Os06g13650	LOC_Os05g46520
LOC_Os06g50310	LOC_Os02g55470	LOC_Os05g47700
LOC_Os06g50480	LOC_Os08g39380	LOC_Os05g47770
LOC_Os06g50600	LOC_Os08g36840	LOC_Os05g47880
LOC_Os06g50830	LOC_Os02g36770	LOC_Os05g47960
LOC_Os06g50900	LOC_Os06g12390	LOC_Os05g48000
LOC_Os06g51070	LOC_Os12g07290	LOC_Os05g48200
LOC_Os07g01270	LOC_Os04g35270	LOC_Os05g48300
LOC_Os07g02060	LOC_Os03g11980	LOC_Os05g48330
LOC_Os07g03220	LOC_Os01g66700	LOC_Os05g48680
LOC_Os07g04560	LOC_Os05g34320	LOC_Os05g48810
LOC_Os07g05010	LOC_Os07g38790	LOC_Os05g48880
LOC_Os07g05720	LOC_Os05g02510	LOC_Os05g49700
LOC_Os07g06470	LOC_Os04g43800	LOC_Os05g49910
LOC_Os07g06620	LOC_Os05g35290	LOC_Os05g50260
LOC_Os07g07974	LOC_Os02g41650	LOC_Os05g50330
LOC_Os07g08140	LOC_Os02g41680	LOC_Os05g50500
LOC_Os07g08420	LOC_Os02g41670	LOC_Os05g50610
LOC_Os07g09590	LOC_Os12g33610	LOC_Os05g50930
LOC_Os07g09740	LOC_Os04g43760	LOC_Os05g51670
LOC_Os07g09830	LOC_Os02g41630	LOC_Os06g01210
LOC_Os07g09860	LOC_Os02g26770	LOC_Os06g01760
LOC_Os07g10890	LOC_Os02g26810	LOC_Os06g01850
LOC_Os07g12210	LOC_Os05g25640	LOC_Os06g01966
LOC_Os07g12340	LOC_Os10g42800	LOC_Os06g02040
LOC_Os07g13260	LOC_Os02g08100	LOC_Os06g02420
LOC_Os07g13920	LOC_Os06g44620	LOC_Os06g02630
LOC_Os07g16330	LOC_Os08g14760	LOC_Os06g03080
LOC_Os07g17160	LOC_Os08g34790	LOC_Os06g04230
LOC_Os07g17180	LOC_Os02g46970	LOC_Os06g04240
LOC_Os07g18840	LOC_Os03g05780	LOC_Os06g04990
LOC_Os07g27330	LOC_Os08g04770	LOC_Os06g05000
LOC_Os07g27340	LOC_Os01g67540	LOC_Os06g05420
LOC_Os07g27670	LOC_Os06g08580	LOC_Os06g05424
LOC_Os07g27770	LOC_Os05g02590	LOC_Os06g05790
LOC_Os07g27800	LOC_Os08g43040	LOC_Os06g06260
LOC_Os07g30774	LOC_Os09g25460	LOC_Os06g06490
LOC_Os07g31400	LOC_Os05g41440	LOC_Os06g07120
LOC_Os07g31410	LOC_Os02g11720	LOC_Os06g08310
LOC_Os07g31420	LOC_Os05g29974	LOC_Os06g08840
LOC_Os07g32110	LOC_Os12g16180	LOC_Os06g09560
LOC_Os07g32170	LOC_Os10g04620	LOC_Os06g09610
LOC_Os07g32510	LOC_Os01g49380	LOC_Os06g09820
LOC_Os07g35870	LOC_Os09g30360	LOC_Os06g11280
LOC_Os07g36170	LOC_Os06g06980	LOC_Os06g11300
LOC_Os07g36460	LOC_Os08g38920	LOC_Os06g11304
LOC_Os07g37630	LOC_Os01g18110	LOC_Os06g11610
LOC_Os07g37920	LOC_Os01g18120	LOC_Os06g12090
LOC_Os07g38030	LOC_Os08g34280	LOC_Os06g12450
LOC_Os07g38410	LOC_Os02g08420	LOC_Os06g12560
LOC_Os07g39220	LOC_Os08g17500	LOC_Os06g13190
LOC_Os07g39470	LOC_Os09g08720	LOC_Os06g13870
LOC_Os07g39480	LOC_Os02g56460	LOC_Os06g14240
LOC_Os07g39800	LOC_Os09g04050	LOC_Os06g15400
LOC_Os07g39820	LOC_Os02g56680	LOC_Os06g18670
LOC_Os07g39940	LOC_Os09g25150	LOC_Os06g19070
LOC_Os07g40000	LOC_Os02g56690	LOC_Os06g19444
LOC_Os07g40020	LOC_Os02g56700	LOC_Os06g21210
LOC_Os07g40570	LOC_Os02g56720	LOC_Os06g21380
LOC_Os07g41580	LOC_Os01g40870	LOC_Os06g21590
LOC_Os07g41720	LOC_Os01g40860	LOC_Os06g23684
LOC_Os07g42400	LOC_Os03g02180	LOC_Os06g25010
LOC_Os07g43030	LOC_Os10g36848	LOC_Os06g28050
LOC_Os07g43530	LOC_Os08g06100	LOC_Os06g28194
LOC_Os07g44200	LOC_Os10g11810	LOC_Os06g28820
LOC_Os07g44690	LOC_Os04g15920	LOC_Os06g30250
LOC_Os07g44950	LOC_Os02g09490	LOC_Os06g30710
LOC_Os07g45450	LOC_Os11g40690	LOC_Os06g31090
LOC_Os07g48180	LOC_Os10g29470	LOC_Os06g33970
LOC_Os07g48260	LOC_Os08g16910	LOC_Os06g34070
LOC_Os07g48450	LOC_Os07g44430	LOC_Os06g35480
LOC_Os07g48550	LOC_Os07g44440	LOC_Os06g35520
LOC_Os07g48570	LOC_Os01g18930	LOC_Os06g35700
LOC_Os07g48630	LOC_Os05g04380	LOC_Os06g36670
LOC_Os07g48660	LOC_Os01g10850	LOC_Os06g36680
LOC_Os07g48820	LOC_Os09g15500	LOC_Os06g37300
LOC_Os08g01330	LOC_Os09g32964	LOC_Os06g37560
LOC_Os08g01700	LOC_Os02g14170	LOC_Os06g37660
LOC_Os08g02160	LOC_Os02g14440	LOC_Os06g38660
LOC_Os08g02300	LOC_Os02g58720	LOC_Os06g39050
LOC_Os08g04390	LOC_Os03g55420	LOC_Os06g39240
LOC_Os08g06140	LOC_Os06g29470	LOC_Os06g39330
LOC_Os08g06659	LOC_Os07g02440	LOC_Os06g39660
LOC_Os08g07740	LOC_Os11g02100	LOC_Os06g40550
LOC_Os08g07970	LOC_Os11g02130	LOC_Os06g41010
LOC_Os08g08160	LOC_Os07g47990	LOC_Os06g42660
LOC_Os08g09690	LOC_Os07g48020	LOC_Os06g44320
LOC_Os08g09800	LOC_Os07g48030	LOC_Os06g44374
LOC_Os08g09810	LOC_Os07g48040	LOC_Os06g44620
LOC_Os08g09840	LOC_Os07g48050	LOC_Os06g45100
LOC_Os08g09900	LOC_Os07g48060	LOC_Os06g46500
LOC_Os08g10080	LOC_Os04g56180	LOC_Os06g46900
LOC_Os08g10560	LOC_Os08g20730	LOC_Os06g46910
LOC_Os08g13840	LOC_Os07g49360	LOC_Os06g46930
LOC_Os08g14880	LOC_Os01g15810	LOC_Os06g47650
LOC_Os08g15510	LOC_Os01g15830	LOC_Os06g47910
LOC_Os08g16370	LOC_Os04g34630	LOC_Os06g48050
LOC_Os08g17100	LOC_Os10g01750	LOC_Os06g48660
LOC_Os08g17400	LOC_Os10g02040	LOC_Os06g48800
LOC_Os08g20486	LOC_Os10g02070	LOC_Os06g49120
LOC_Os08g23880	LOC_Os10g39170	LOC_Os06g49160
LOC_Os08g25260	LOC_Os01g36240	LOC_Os06g50230
LOC_Os08g26880	LOC_Os01g22230	LOC_Os06g50930
LOC_Os08g27740	LOC_Os01g22249	LOC_Os06g50960
LOC_Os08g28214	LOC_Os01g22352	LOC_Os06g51050
LOC_Os08g29500	LOC_Os11g10460	LOC_Os06g51060
LOC_Os08g29660	LOC_Os07g06190	LOC_Os07g01820
LOC_Os08g31080	LOC_Os07g06300	LOC_Os07g01904
LOC_Os08g32440	LOC_Os07g06380	LOC_Os07g03288
LOC_Os08g33270	LOC_Os07g44460	LOC_Os07g03580
LOC_Os08g33530	LOC_Os07g44550	LOC_Os07g03590
LOC_Os08g33590	LOC_Os07g44590	LOC_Os07g03710
LOC_Os08g33670	LOC_Os03g25280	LOC_Os07g03730
LOC_Os08g33910	LOC_Os03g25300	LOC_Os07g05360
LOC_Os08g34010	LOC_Os03g25330	LOC_Os07g05400
LOC_Os08g36450	LOC_Os03g25340	LOC_Os07g05940
LOC_Os08g36700	LOC_Os03g25370	LOC_Os07g06990
LOC_Os08g36740	LOC_Os04g39100	LOC_Os07g07350
LOC_Os08g36790	LOC_Os09g29490	LOC_Os07g07480
LOC_Os08g37290	LOC_Os01g07770	LOC_Os07g08150
LOC_Os08g37400	LOC_Os08g42030	LOC_Os07g08350
LOC_Os08g37730	LOC_Os01g19020	LOC_Os07g08420
LOC_Os08g37810	LOC_Os06g20150	LOC_Os07g08729
LOC_Os08g38020	LOC_Os07g06175	LOC_Os07g09050
LOC_Os08g38080	LOC_Os03g32050	LOC_Os07g09140
LOC_Os08g38210	LOC_Os06g13050	LOC_Os07g11330
LOC_Os08g38220	LOC_Os06g27850	LOC_Os07g12390
LOC_Os08g38780	LOC_Os07g01370	LOC_Os07g14600
LOC_Os08g38990	LOC_Os07g01410	LOC_Os07g15460
LOC_Os08g39630	LOC_Os02g50770	LOC_Os07g16364
LOC_Os08g39830	LOC_Os03g02920	LOC_Os07g17184
LOC_Os08g39890	LOC_Os03g02939	LOC_Os07g17330
LOC_Os08g40030	LOC_Os06g16350	LOC_Os07g18750
LOC_Os08g40260	LOC_Os12g02060	LOC_Os07g18874
LOC_Os08g41320	LOC_Os12g02080	LOC_Os07g20270
LOC_Os08g41940	LOC_Os12g08920	LOC_Os07g20340
LOC_Os08g42400	LOC_Os05g04440	LOC_Os07g22400
LOC_Os08g42470	LOC_Os05g04450	LOC_Os07g22600
LOC_Os08g43070	LOC_Os05g04470	LOC_Os07g23120
LOC_Os08g43090	LOC_Os08g02110	LOC_Os07g23410
LOC_Os08g43160	LOC_Os01g16450	LOC_Os07g23430
LOC_Os08g43334	LOC_Os06g48000	LOC_Os07g23640
LOC_Os08g43410	LOC_Os06g48010	LOC_Os07g23660
LOC_Os08g43600	LOC_Os06g48020	LOC_Os07g24230
LOC_Os08g44820	LOC_Os06g48030	LOC_Os07g28260
LOC_Os08g44940	LOC_Os02g06630	LOC_Os07g29310
LOC_Os09g01140	LOC_Os03g13180	LOC_Os07g29640
LOC_Os09g01470	LOC_Os03g13200	LOC_Os07g30670
LOC_Os09g01780	LOC_Os03g13210	LOC_Os07g32570
LOC_Os09g08460	LOC_Os04g42100	LOC_Os07g32620
LOC_Os09g09630	LOC_Os04g59160	LOC_Os07g32630
LOC_Os09g10840	LOC_Os04g59190	LOC_Os07g33200
LOC_Os09g11390	LOC_Os04g59200	LOC_Os07g33420
LOC_Os09g11870	LOC_Os04g59210	LOC_Os07g34260
LOC_Os09g12380	LOC_Os04g59260	LOC_Os07g34280
LOC_Os09g13570	LOC_Os03g22010	LOC_Os07g34570
LOC_Os09g13575	LOC_Os03g05770	LOC_Os07g35060
LOC_Os09g14040	LOC_Os07g31610	LOC_Os07g35310
LOC_Os09g16510	LOC_Os06g32960	LOC_Os07g35340
LOC_Os09g19950	LOC_Os06g32980	LOC_Os07g35370
LOC_Os09g23230	LOC_Os06g32990	LOC_Os07g35480
LOC_Os09g24480	LOC_Os06g33080	LOC_Os07g35560
LOC_Os09g24490	LOC_Os06g33100	LOC_Os07g36190
LOC_Os09g24560	LOC_Os10g41720	LOC_Os07g36560
LOC_Os09g24810	LOC_Os01g57730	LOC_Os07g37180
LOC_Os09g24820	LOC_Os02g14180	LOC_Os07g37400
LOC_Os09g25040	LOC_Os06g46799	LOC_Os07g37420
LOC_Os09g25060	LOC_Os07g34710	LOC_Os07g37454
LOC_Os09g25070	LOC_Os01g73170	LOC_Os07g37690
LOC_Os09g25510	LOC_Os01g73200	LOC_Os07g37850
LOC_Os09g27190	LOC_Os03g36560	LOC_Os07g38000
LOC_Os09g27850	LOC_Os05g41990	LOC_Os07g38550
LOC_Os09g28200	LOC_Os05g42000	LOC_Os07g39020
LOC_Os09g28210	LOC_Os11g43980	LOC_Os07g39390
LOC_Os09g28310	LOC_Os12g34524	LOC_Os07g39830
LOC_Os09g28354	LOC_Os03g55410	LOC_Os07g40220
LOC_Os09g28900	LOC_Os01g22370	LOC_Os07g41060
LOC_Os09g29130	LOC_Os02g14430	LOC_Os07g42490
LOC_Os09g29360	LOC_Os04g55740	LOC_Os07g42650
LOC_Os09g29820	LOC_Os07g48010	LOC_Os07g42750
LOC_Os09g29830	LOC_Os06g35520	LOC_Os07g43330
LOC_Os09g29930	LOC_Os06g35480	LOC_Os07g43570
LOC_Os09g29960	LOC_Os05g04500	LOC_Os07g43950
LOC_Os09g30310	LOC_Os05g06970	LOC_Os07g44160
LOC_Os09g30400	LOC_Os06g35490	LOC_Os07g44330
LOC_Os09g31300	LOC_Os04g59150	LOC_Os07g44690
LOC_Os09g31390	LOC_Os02g14160	LOC_Os07g45060
LOC_Os09g31400	LOC_Os01g51550	LOC_Os07g46190
LOC_Os09g31438	LOC_Os03g22020	LOC_Os07g46450
LOC_Os09g32040	LOC_Os06g21570	LOC_Os07g46540
LOC_Os09g32260	LOC_Os01g67220	LOC_Os07g46852
LOC_Os09g32510	LOC_Os03g11420	LOC_Os07g47140
LOC_Os09g32944	LOC_Os05g30350	LOC_Os07g47480
LOC_Os09g33490	LOC_Os08g39860	LOC_Os07g47530
LOC_Os09g33580	LOC_Os09g31430	LOC_Os07g47790
LOC_Os09g34060	LOC_Os10g17650	LOC_Os07g48010
LOC_Os09g34330	LOC_Os04g39900	LOC_Os07g48100
LOC_Os09g34950	LOC_Os01g70520	LOC_Os07g48710
LOC_Os09g35790	LOC_Os05g30250	LOC_Os07g48760
LOC_Os09g36160	LOC_Os09g33680	LOC_Os07g48830
LOC_Os09g36910	LOC_Os11g08120	LOC_Os07g49400
LOC_Os09g37710	LOC_Os05g30300	LOC_Os08g01140
LOC_Os09g38000	LOC_Os06g46940	LOC_Os08g01380
LOC_Os09g38010	LOC_Os09g33710	LOC_Os08g01770
LOC_Os09g38570	LOC_Os09g31410	LOC_Os08g01910
LOC_Os09g39490	LOC_Os08g39870	LOC_Os08g02200
LOC_Os10g01530	LOC_Os03g53860	LOC_Os08g02730
LOC_Os10g02509	LOC_Os03g53800	LOC_Os08g03579
LOC_Os10g02584	LOC_Os03g53790	LOC_Os08g04170
LOC_Os10g02620	LOC_Os01g56510	LOC_Os08g04340
LOC_Os10g06860	LOC_Os05g37700	LOC_Os08g04450
LOC_Os10g07510	LOC_Os02g03870	LOC_Os08g04540
LOC_Os10g09820	LOC_Os04g43360	LOC_Os08g04840
LOC_Os10g11580	LOC_Os03g49600	LOC_Os08g06450
LOC_Os10g14040	LOC_Os03g49610	LOC_Os08g06510
LOC_Os10g18099	LOC_Os12g23170	LOC_Os08g06700
LOC_Os10g19270	LOC_Os04g43390	LOC_Os08g07100
LOC_Os10g21560	LOC_Os04g43400	LOC_Os08g07600
LOC_Os10g22430	LOC_Os01g32364	LOC_Os08g07730
LOC_Os10g22950	LOC_Os04g43410	LOC_Os08g07840
LOC_Os10g23050	LOC_Os02g06640	LOC_Os08g10020
LOC_Os10g25620	LOC_Os04g53620	LOC_Os08g10080
LOC_Os10g25640	LOC_Os10g33620	LOC_Os08g10612
LOC_Os10g25850	LOC_Os09g27930	LOC_Os08g10730
LOC_Os10g26240	LOC_Os07g30640	LOC_Os08g12160
LOC_Os10g26410	LOC_Os07g30990	LOC_Os08g16330
LOC_Os10g26460	LOC_Os07g31110	LOC_Os08g16830
LOC_Os10g26620	LOC_Os03g13170	LOC_Os08g17140
LOC_Os10g27360	LOC_Os05g06770	LOC_Os08g17500
LOC_Os10g27380	LOC_Os01g22490	LOC_Os08g17784
LOC_Os10g27390	LOC_Os10g34990	LOC_Os08g23870
LOC_Os10g28340	LOC_Os07g49230	LOC_Os08g24390
LOC_Os10g32070	LOC_Os03g18380	LOC_Os08g26120
LOC_Os10g33760	LOC_Os11g01510	LOC_Os08g29600
LOC_Os10g34884	LOC_Os11g30410	LOC_Os08g31930
LOC_Os10g35300	LOC_Os01g16540	LOC_Os08g31980
LOC_Os10g36420	LOC_Os03g57790	LOC_Os08g32060
LOC_Os10g37240	LOC_Os07g07240	LOC_Os08g32330
LOC_Os10g38820	LOC_Os05g08960	LOC_Os08g32600
LOC_Os10g38834	LOC_Os01g16650	LOC_Os08g32920
LOC_Os10g39750	LOC_Os01g60360	LOC_Os08g33140
LOC_Os10g40390	LOC_Os04g57220	LOC_Os08g33488
LOC_Os10g40740	LOC_Os09g12230	LOC_Os08g33820
LOC_Os10g40810	LOC_Os01g46926	LOC_Os08g35240
LOC_Os10g41460	LOC_Os07g38940	LOC_Os08g35760
LOC_Os10g41770	LOC_Os01g62244	LOC_Os08g36910
LOC_Os10g42130	LOC_Os05g38550	LOC_Os08g37500
LOC_Os10g42160	LOC_Os10g31000	LOC_Os08g37520
LOC_Os10g42430	LOC_Os04g49130	LOC_Os08g37660
LOC_Os10g42850	LOC_Os10g39120	LOC_Os08g37700
LOC_Os11g01550	LOC_Os03g03130	LOC_Os08g37720
LOC_Os11g02470	LOC_Os03g19500	LOC_Os08g37874
LOC_Os11g02480	LOC_Os06g09330	LOC_Os08g37990
LOC_Os11g02520	LOC_Os08g28680	LOC_Os08g38170
LOC_Os11g02530	LOC_Os10g11260	LOC_Os08g39190
LOC_Os11g02540	LOC_Os09g15320	LOC_Os08g39560
LOC_Os11g02964	LOC_Os01g48280	LOC_Os08g40560
LOC_Os11g03110	LOC_Os05g48380	LOC_Os08g40680
LOC_Os11g03300	LOC_Os09g12310	LOC_Os08g40910
LOC_Os11g03310	LOC_Os01g13170	LOC_Os08g41910
LOC_Os11g03370	LOC_Os01g13280	LOC_Os08g41960
LOC_Os11g03420	LOC_Os05g14300	LOC_Os08g42350
LOC_Os11g04360	LOC_Os01g03520	LOC_Os08g43090
LOC_Os11g04400	LOC_Os01g48580	LOC_Os08g44770
LOC_Os11g04470	LOC_Os06g45000	LOC_Os08g45140
LOC_Os11g04570	LOC_Os03g47770	LOC_Os09g02810
LOC_Os11g04590	LOC_Os12g44000	LOC_Os09g04710
LOC_Os11g04960	LOC_Os01g70140	LOC_Os09g05020
LOC_Os11g05340	LOC_Os03g47949	LOC_Os09g06464
LOC_Os11g05480	LOC_Os05g06690	LOC_Os09g08130
LOC_Os11g05614	LOC_Os05g03100	LOC_Os09g08280
LOC_Os11g05640	LOC_Os05g38830	LOC_Os09g10000
LOC_Os11g06010	LOC_Os02g01170	LOC_Os09g12150
LOC_Os11g06170	LOC_Os03g31400	LOC_Os09g12290
LOC_Os11g06180	LOC_Os05g01460	LOC_Os09g13570
LOC_Os11g06410	LOC_Os08g02140	LOC_Os09g13650
LOC_Os11g07460	LOC_Os10g32880	LOC_Os09g15050
LOC_Os11g07700	LOC_Os01g03400	LOC_Os09g15540
LOC_Os11g08210	LOC_Os07g46560	LOC_Os09g17560
LOC_Os11g08410	LOC_Os01g03170	LOC_Os09g19970
LOC_Os11g11100	LOC_Os01g03290	LOC_Os09g20400
LOC_Os11g11220	LOC_Os02g03620	LOC_Os09g21120
LOC_Os11g11600	LOC_Os05g06060	LOC_Os09g21370
LOC_Os11g12490	LOC_Os05g06100	LOC_Os09g21460
LOC_Os11g13930	LOC_Os06g20610	LOC_Os09g25430
LOC_Os11g14020	LOC_Os01g13370	LOC_Os09g25784
LOC_Os11g15210	LOC_Os02g19140	LOC_Os09g26670
LOC_Os11g15755	LOC_Os03g24040	LOC_Os09g26810
LOC_Os11g16290	LOC_Os01g55640	LOC_Os09g27260
LOC_Os11g17430	LOC_Os02g53140	LOC_Os09g27990
LOC_Os11g19030	LOC_Os03g05270	LOC_Os09g28310
LOC_Os11g25560	LOC_Os10g31850	LOC_Os09g28480
LOC_Os11g29870	LOC_Os01g52110	LOC_Os09g28580
LOC_Os11g30370	LOC_Os03g22680	LOC_Os09g28620
LOC_Os11g31100	LOC_Os12g35320	LOC_Os09g28690
LOC_Os11g31330	LOC_Os06g06870	LOC_Os09g29130
LOC_Os11g31340	LOC_Os06g19680	LOC_Os09g29710
LOC_Os11g31360	LOC_Os01g01700	LOC_Os09g30410
LOC_Os11g31370	LOC_Os02g47870	LOC_Os09g31130
LOC_Os11g31380	LOC_Os01g27150	LOC_Os09g31430
LOC_Os11g32100	LOC_Os01g27160	LOC_Os09g32690
LOC_Os11g34200	LOC_Os07g43180	LOC_Os09g33530
LOC_Os11g38870	LOC_Os08g28800	LOC_Os09g34214
LOC_Os11g39000	LOC_Os08g28820	LOC_Os09g34920
LOC_Os11g41640	LOC_Os06g02360	LOC_Os09g35010
LOC_Os11g45530	LOC_Os11g26910	LOC_Os09g35020
LOC_Os11g45850	LOC_Os02g13180	LOC_Os09g35030
LOC_Os11g45920	LOC_Os07g22680	LOC_Os09g35790
LOC_Os11g45950	LOC_Os09g36830	LOC_Os09g36470
LOC_Os11g47870	LOC_Os02g01160	LOC_Os09g36680
LOC_Os11g47890	LOC_Os07g43240	LOC_Os09g36880
LOC_Os11g47900	LOC_Os07g43250	LOC_Os09g37180
LOC_Os11g47910	LOC_Os07g43260	LOC_Os09g37480
LOC_Os11g47920	LOC_Os09g10054	LOC_Os09g37500
LOC_Os12g01180	LOC_Os09g10200	LOC_Os09g38090
LOC_Os12g01550	LOC_Os09g10270	LOC_Os09g38310
LOC_Os12g02090	LOC_Os09g10300	LOC_Os09g38620
LOC_Os12g02400	LOC_Os10g30200	LOC_Os09g38710
LOC_Os12g02420	LOC_Os05g35110	LOC_Os09g38920
LOC_Os12g02440	LOC_Os01g65920	LOC_Os10g02260
LOC_Os12g02450	LOC_Os11g48030	LOC_Os10g02880
LOC_Os12g02470	LOC_Os08g07400	LOC_Os10g03669
LOC_Os12g02870	LOC_Os04g55030	LOC_Os10g03730
LOC_Os12g03040	LOC_Os02g51180	LOC_Os10g03740
LOC_Os12g03050	LOC_Os05g51480	LOC_Os10g03870
LOC_Os12g03110	LOC_Os01g04870	LOC_Os10g04780
LOC_Os12g03660	LOC_Os02g20430	LOC_Os10g05250
LOC_Os12g04200	LOC_Os01g01484	LOC_Os10g05950
LOC_Os12g04230	LOC_Os07g22840	LOC_Os10g05970
LOC_Os12g04370	LOC_Os03g19059	LOC_Os10g05980
LOC_Os12g04380	LOC_Os04g40830	LOC_Os10g05990
LOC_Os12g05680	LOC_Os04g51110	LOC_Os10g06510
LOC_Os12g05940	LOC_Os09g06680	LOC_Os10g07210
LOC_Os12g05990	LOC_Os02g47180	LOC_Os10g07270
LOC_Os12g06010	LOC_Os03g03150	LOC_Os10g07400
LOC_Os12g06200	LOC_Os01g74146	LOC_Os10g08474
LOC_Os12g06380	LOC_Os06g41750	LOC_Os10g12750
LOC_Os12g06520	LOC_Os12g43120	LOC_Os10g13850
LOC_Os12g06540	LOC_Os03g13370	LOC_Os10g14050
LOC_Os12g06640	LOC_Os05g05720	LOC_Os10g17260
LOC_Os12g07120	LOC_Os02g43920	LOC_Os10g18370
LOC_Os12g07480	LOC_Os05g50360	LOC_Os10g20230
LOC_Os12g07790	LOC_Os07g44004	LOC_Os10g21130
LOC_Os12g09250	LOC_Os06g50060	LOC_Os10g21670
LOC_Os12g10630	LOC_Os03g43910	LOC_Os10g22520
LOC_Os12g12380	LOC_Os01g74200	LOC_Os10g25030
LOC_Os12g12970	LOC_Os05g25910	LOC_Os10g25060
LOC_Os12g13170	LOC_Os03g62500	LOC_Os10g25130
LOC_Os12g18910	LOC_Os01g53160	LOC_Os10g25230
LOC_Os12g22630	LOC_Os03g51970	LOC_Os10g25290
LOC_Os12g22940	LOC_Os03g07430	LOC_Os10g26940
LOC_Os12g23090	LOC_Os07g38170	LOC_Os10g27380
LOC_Os12g24400	LOC_Os06g39480	LOC_Os10g27980
LOC_Os12g29330	LOC_Os05g09520	LOC_Os10g28050
LOC_Os12g31430	LOC_Os03g60710	LOC_Os10g28080
LOC_Os12g32140	LOC_Os02g04680	LOC_Os10g29470
LOC_Os12g32250	LOC_Os10g37640	LOC_Os10g30162
LOC_Os12g32400	LOC_Os05g38420	LOC_Os10g30640
LOC_Os12g37410	LOC_Os02g47280	LOC_Os10g31330
LOC_Os12g37415	LOC_Os01g09470	LOC_Os10g32550
LOC_Os12g38200	LOC_Os06g44034	LOC_Os10g32590
LOC_Os12g38490	LOC_Os01g64430	LOC_Os10g32810
LOC_Os12g39850	LOC_Os01g64380	LOC_Os10g33040
LOC_Os12g39990	LOC_Os01g12690	LOC_Os10g33050
LOC_Os12g40570	LOC_Os09g33580	LOC_Os10g33650
LOC_Os12g40590	LOC_Os06g49010	LOC_Os10g33730
LOC_Os12g40630	LOC_Os03g16740	LOC_Os10g34910
LOC_Os12g40710	LOC_Os06g46410	LOC_Os10g34920
LOC_Os12g40730	LOC_Os01g12890	LOC_Os10g34930
LOC_Os12g40920	LOC_Os05g02020	LOC_Os10g36160
LOC_Os12g41210	LOC_Os06g09660	LOC_Os10g36500
LOC_Os12g41230	LOC_Os04g38720	LOC_Os10g37160
LOC_Os12g41650	LOC_Os03g13400	LOC_Os10g37260
LOC_Os12g41680	LOC_Os02g51320	LOC_Os10g38470
LOC_Os12g41880	LOC_Os08g06480	LOC_Os10g38580
LOC_Os12g42190	LOC_Os06g08340	LOC_Os10g38610
LOC_Os12g42400	LOC_Os08g07760	LOC_Os10g38740
LOC_Os12g42610	LOC_Os01g58420	LOC_Os10g38870
LOC_Os12g42970	LOC_Os04g57610	LOC_Os10g38940
LOC_Os12g43530	LOC_Os06g15990	LOC_Os10g39170
LOC_Os12g43620	LOC_Os02g02890	LOC_Os10g39500
LOC_Os12g43790	LOC_Os03g44500	LOC_Os10g39680
LOC_Os01g13520	LOC_Os04g38870	LOC_Os10g40420
LOC_Os01g48060	LOC_Os07g13170	LOC_Os10g40480
LOC_Os01g54990	LOC_Os03g03070	LOC_Os10g40530

LOC_Os01g52170	LOC_Os07g44430
LOC_Os01g53890	LOC_Os08g15308
LOC_Os01g54370	LOC_Os10g21332
LOC_Os01g56350	LOC_Os10g35690
LOC_Os04g55060	LOC_Os03g56300
LOC_Os08g09950	LOC_Os11g24500
LOC_Os05g30240	LOC_Os08g25090
LOC_Os10g21290	LOC_Os02g47760
LOC_Os03g61890	LOC_Os07g41260
LOC_Os07g49110	LOC_Os11g26030
LOC_Os04g49440	LOC_Os02g03320
LOC_Os08g27840	LOC_Os12g34092
LOC_Os08g44290	LOC_Os01g58010
LOC_Os08g41790	LOC_Os07g33240
LOC_Os08g48480	LOC_Os07g44650
LOC_Os02g48610	LOC_Os07g44740
LOC_Os02g24354	LOC_Os03g17590
LOC_Os07g08430	LOC_Os03g02390
LOC_Os08g38980	LOC_Os10g32550
LOC_Os08g04450	LOC_Os04g41300
LOC_Os08g33440	LOC_Os04g38630
LOC_Os08g39160	LOC_Os01g47690
LOC_Os08g43560	LOC_Os10g37690
LOC_Os08g42950	LOC_Os10g35760
LOC_Os08g38086	LOC_Os10g35770
LOC_Os08g07060	LOC_Os07g26540
LOC_Os08g02060	LOC_Os01g16510
LOC_Os08g44400	LOC_Os01g17320
LOC_Os08g44530	LOC_Os01g14020
LOC_Os08g07540	LOC_Os11g02080
LOC_Os09g29080	LOC_Os11g03650
LOC_Os09g29190	LOC_Os12g23620
LOC_Os08g33700	LOC_Os09g31190
LOC_Os02g47020	LOC_Os12g23610
LOC_Os08g34720	LOC_Os10g35750
LOC_Os02g47510	LOC_Os10g30970
LOC_Os02g47620	LOC_Os12g02385
LOC_Os07g19460	LOC_Os11g40750
LOC_Os02g03010	LOC_Os09g39550
LOC_Os02g10940	LOC_Os11g34840
LOC_Os02g51790	LOC_Os11g34760
LOC_Os02g48880	LOC_Os11g34750
LOC_Os02g35900	LOC_Os11g34830
LOC_Os02g36210	LOC_Os08g29950
LOC_Os07g41710	LOC_Os02g22100
LOC_Os07g20544	LOC_Os02g19150
LOC_Os08g37605	LOC_Os02g05780
LOC_Os08g10010	LOC_Os03g59600
LOC_Os02g20490	LOC_Os03g60000
LOC_Os08g19850	LOC_Os03g02480
LOC_Os08g23410	LOC_Os03g56540
LOC_Os07g07310	LOC_Os03g49140
LOC_Os07g49400	LOC_Os03g42880
LOC_Os08g44860	LOC_Os02g45100
LOC_Os02g39600	LOC_Os01g25660
LOC_Os10g62500	LOC_Os10g16910
LOC_Os02g27730	LOC_Os01g13150
LOC_Os01g37510	LOC_Os01g57082
LOC_Os12g05650	LOC_Os01g55700
LOC_Os12g02589	LOC_Os01g54950
LOC_Os12g42300	LOC_Os01g53720
LOC_Os12g29570	LOC_Os01g51410
LOC_Os12g19304	LOC_Os11g37780
LOC_Os05g10810	LOC_Os01g51390
LOC_Os01g60650	LOC_Os12g10200
LOC_Os01g47630	LOC_Os07g12530
LOC_Os01g52864	LOC_Os07g38280
LOC_Os05g33230	LOC_Os03g05430
LOC_Os06g15400	LOC_Os03g26000
LOC_Os06g37224	LOC_Os03g45990
LOC_Os06g37330	LOC_Os03g30220
LOC_Os06g37364	LOC_Os02g35630
LOC_Os08g16010	LOC_Os06g40940
LOC_Os06g29220	LOC_Os06g35470
LOC_Os06g09679	LOC_Os07g44330
LOC_Os06g09698	LOC_Os03g27340
LOC_Os06g24730	LOC_Os03g26030
LOC_Os06g11140	LOC_Os04g32020
LOC_Os01g46380	LOC_Os03g24050
LOC_Os06g05400	LOC_Os04g24410
LOC_Os01g45070	LOC_Os04g24520
LOC_Os02g33500	LOC_Os03g15690
LOC_Os02g33580	LOC_Os03g39380
LOC_Os02g21520	LOC_Os06g02980
LOC_Os11g41160	LOC_Os07g09220
LOC_Os11g40600	LOC_Os05g45750
LOC_Os12g39370	LOC_Os05g29930
LOC_Os06g10210	LOC_Os04g44290
LOC_Os09g26730	LOC_Os08g27630
LOC_Os06g11400	LOC_Os08g16480
LOC_Os07g08150	LOC_Os08g06474
LOC_Os08g02390	LOC_Os07g40620
LOC_Os06g28194	LOC_Os07g38260
LOC_Os09g29950	LOC_Os06g09260
LOC_Os09g39034	LOC_Os06g07474
LOC_Os09g39180	LOC_Os05g15350
LOC_Os09g39230	LOC_Os04g51290
LOC_Os09g39260	LOC_Os02g54910
LOC_Os01g39270	LOC_Os01g10290
LOC_Os01g20110	LOC_Os01g70780
LOC_Os06g30380	LOC_Os06g15700
LOC_Os06g45820	LOC_Os11g06320
LOC_Os01g02080	LOC_Os06g07160
LOC_Os02g39160	LOC_Os06g09688
LOC_Os02g52260	LOC_Os06g40840
LOC_Os02g56030	LOC_Os06g04560
LOC_Os02g56040	LOC_Os12g38790
LOC_Os02g54060	LOC_Os12g42960
LOC_Os02g53810	LOC_Os09g39960
LOC_Os02g57010	LOC_Os06g03980
LOC_Os02g57030	LOC_Os02g56540
LOC_Os02g54980	LOC_Os02g28850
LOC_Os06g29180	LOC_Os03g50520
LOC_Os01g54030	LOC_Os05g38480
LOC_Os11g47970	LOC_Os01g15540
LOC_Os01g48990	LOC_Os01g49200
LOC_Os06g50130	LOC_Os01g60040
LOC_Os01g10280	LOC_Os05g06260
LOC_Os06g51029	LOC_Os10g40620
LOC_Os01g11054	LOC_Os01g70560
LOC_Os06g47940	LOC_Os01g69130
LOC_Os05g34630	LOC_Os10g20510
LOC_Os01g56400	LOC_Os07g01490
LOC_Os01g04920	LOC_Os07g33630
LOC_Os01g43120	LOC_Os07g02470
LOC_Os09g21520	LOC_Os04g54940
LOC_Os02g07680	LOC_Os10g35580
LOC_Os09g10750	LOC_Os01g56090
LOC_Os09g10760	LOC_Os03g38480
LOC_Os02g43350	LOC_Os04g31190
LOC_Os05g29860	LOC_Os07g06720
LOC_Os01g43150	LOC_Os05g45980
LOC_Os01g63410	LOC_Os05g33890
LOC_Os03g07234	LOC_Os06g13600
LOC_Os03g07310	LOC_Os12g07880
LOC_Os02g39730	LOC_Os01g60050
LOC_Os05g42100	LOC_Os02g50640
LOC_Os05g04220	LOC_Os04g04060
LOC_Os05g33520	LOC_Os01g48900
LOC_Os05g33300	LOC_Os02g02690
LOC_Os01g55570	LOC_Os08g42189
LOC_Os01g01280	LOC_Os11g37100
LOC_Os01g64270	LOC_Os09g11440
LOC_Os01g31610	
LOC_Os01g58790	
LOC_Os10g40110	
LOC_Os01g69080	
LOC_Os08g33380	
LOC_Os02g02550	
LOC_Os05g32390	
LOC_Os10g39150	
LOC_Os03g50530	
LOC_Os01g14410	
LOC_Os01g67570	
LOC_Os02g44000	
LOC_Os05g08930	
LOC_Os05g38170	
LOC_Os03g48000	
LOC_Os05g37780	
LOC_Os05g42350	
LOC_Os01g05080	
LOC_Os05g01950	
LOC_Os05g02060	
LOC_Os05g49800	
LOC_Os05g05600	
LOC_Os12g44360	
LOC_Os01g55350	
LOC_Os01g34770	
LOC_Os01g54020	
LOC_Os01g74280	
LOC_Os01g73420	
LOC_Os01g70300	
LOC_Os11g01380	
LOC_Os11g02180	
LOC_Os11g04670	
LOC_Os11g05990	
LOC_Os11g34180	
LOC_Os11g34940	
LOC_Os12g31460	
LOC_Os12g31820	
LOC_Os12g33020	
LOC_Os12g01390	
LOC_Os12g02120	
LOC_Os12g04440	
LOC_Os12g08170	
LOC_Os12g09000	
LOC_Os12g13460	
LOC_Os10g39930	
LOC_Os12g14070	
LOC_Os02g13970	
LOC_Os02g14770	
LOC_Os02g45880	
LOC_Os02g17780	
LOC_Os02g44570	
LOC_Os09g24710	
LOC_Os07g38300	
LOC_Os07g28610	
LOC_Os02g24634	
LOC_Os04g16732	
LOC_Os10g21192	
LOC_Os10g21418	
LOC_Os10g38206	
LOC_Os06g09610	
LOC_Os05g13390	
LOC_Os01g68450	
LOC_Os03g56280	
LOC_Os03g50080	
LOC_Os11g24500	
LOC_Os01g09320	
LOC_Os02g51570	
LOC_Os11g19340	
LOC_Os11g19290	
LOC_Os10g37980	
LOC_Os11g29850	
LOC_Os04g50880	
LOC_Os03g17520	
LOC_Os03g12560	
LOC_Os06g36800	
LOC_Os04g38600	
LOC_Os04g55780	
LOC_Os07g43810	
LOC_Os01g39250	
LOC_Os01g39260	
LOC_Os03g06200	
LOC_Os01g65830	
LOC_Os01g56890	
LOC_Os12g24800	
LOC_Os03g44380	
LOC_Os12g42280	
LOC_Os01g17150	
LOC_Os01g12220	
LOC_Os01g13190	
LOC_Os10g41940	
LOC_Os12g44310	
LOC_Os09g20320	
LOC_Os09g21250	
LOC_Os09g14670	
LOC_Os09g15240	
LOC_Os12g12370	
LOC_Os10g35010	
LOC_Os12g09570	
LOC_Os10g30970	
LOC_Os10g25570	
LOC_Os10g08980	
LOC_Os11g27980	
LOC_Os09g07630	
LOC_Os08g42690	
LOC_Os08g37820	
LOC_Os08g31730	
LOC_Os08g32130	
LOC_Os01g70950	
LOC_Os01g67450	
LOC_Os01g66379	
LOC_Os02g18940	
LOC_Os02g05780	
LOC_Os01g40820	
LOC_Os01g13390	
LOC_Os03g02570	
LOC_Os03g56320	
LOC_Os03g53010	
LOC_Os03g52300	
LOC_Os02g57020	
LOC_Os03g49450	
LOC_Os02g46750	
LOC_Os02g44200	
LOC_Os02g44134	
LOC_Os02g42170	
LOC_Os02g39340	
LOC_Os02g33010	
LOC_Os01g34450	
LOC_Os01g34470	
LOC_Os01g55880	
LOC_Os01g51410	
LOC_Os03g29730	
LOC_Os07g12530	
LOC_Os03g59146	
LOC_Os04g24165	
LOC_Os05g25930	
LOC_Os01g01160	
LOC_Os04g46710	
LOC_Os08g02570	
LOC_Os08g23160	
LOC_Os05g01970	
LOC_Os11g41910	
LOC_Os06g09688	
LOC_Os06g45930	
LOC_Os06g40940	
LOC_Os06g30780	
LOC_Os06g28950	
LOC_Os03g27340	
LOC_Os03g21650	
LOC_Os04g33220	
LOC_Os04g31040	
LOC_Os06g05100	
LOC_Os07g13969	
LOC_Os06g02980	
LOC_Os07g06550	
LOC_Os07g06450	
LOC_Os05g27100	
LOC_Os08g02560	
LOC_Os07g46310	
LOC_Os04g45200	
LOC_Os04g44290	
LOC_Os04g34000	
LOC_Os08g30830	
LOC_Os08g28410	
LOC_Os08g16480	
LOC_Os07g32774	
LOC_Os05g18790	
LOC_Os04g58900	
LOC_Os04g58620	

LOC_Os01g70270
LOC_Os02g04810
LOC_Os02g06910
LOC_Os02g35140
LOC_Os02g41800
LOC_Os04g36054
LOC_Os04g43910
LOC_Os04g56850
LOC_Os04g57610
LOC_Os04g59430
LOC_Os05g43920
LOC_Os05g48870
LOC_Os06g09660
LOC_Os06g46410
LOC_Os06g47150
LOC_Os06g48950
LOC_Os07g08520
LOC_Os07g08540
LOC_Os07g08600
LOC_Os08g40900
LOC_Os10g33940
LOC_Os11g32110
LOC_Os12g29520
LOC_Os12g41950
LOC_Os01g13300
LOC_Os01g51610
LOC_Os01g52514
LOC_Os01g52540
LOC_Os01g67830
LOC_Os01g68370
LOC_Os02g10420
LOC_Os02g25820
LOC_Os02g25830
LOC_Os02g38470
LOC_Os02g45850
LOC_Os03g02900
LOC_Os03g06850
LOC_Os03g08620
LOC_Os03g11370
LOC_Os03g42230
LOC_Os03g42240
LOC_Os03g42280
LOC_Os03g42290
LOC_Os03g42370
LOC_Os03g42410
LOC_Os03g42420
LOC_Os03g42430
LOC_Os04g27960
LOC_Os04g27990
LOC_Os04g28000
LOC_Os04g31730
LOC_Os04g49230
LOC_Os04g58000
LOC_Os04g58010
LOC_Os05g40280
LOC_Os06g01860
LOC_Os06g02230
LOC_Os06g09420
LOC_Os06g42630
LOC_Os07g08530
LOC_Os07g12820
LOC_Os07g17230
LOC_Os07g37610
LOC_Os07g48200
LOC_Os08g01090
LOC_Os08g06120
LOC_Os08g23470
LOC_Os08g23570
LOC_Os10g07060
LOC_Os10g17630
LOC_Os10g39190
LOC_Os11g05740
LOC_Os11g09160
LOC_Os12g06080
LOC_Os12g40070
LOC_Os12g40080
LOC_Os12g40090
LOC_Os12g40120
LOC_Os01g67410
LOC_Os02g40070
LOC_Os03g56050
LOC_Os03g60430
LOC_Os04g42570
LOC_Os04g55560
LOC_Os04g55970
LOC_Os05g03040
LOC_Os05g45954
LOC_Os06g05340
LOC_Os06g43220
LOC_Os07g13170
LOC_Os11g03540
LOC_Os11g19060
LOC_Os12g03290
LOC_Os03g19900
LOC_Os01g04020
LOC_Os01g07120
LOC_Os01g10370
LOC_Os01g12440
LOC_Os01g21120
LOC_Os01g46870
LOC_Os01g54890
LOC_Os01g58420
LOC_Os01g59780
LOC_Os01g64790
LOC_Os01g66270
LOC_Os01g73770
LOC_Os02g06330
LOC_Os02g09650
LOC_Os02g10760
LOC_Os02g13710
LOC_Os02g29550
LOC_Os02g32040
LOC_Os02g32140
LOC_Os02g34260
LOC_Os02g34270
LOC_Os02g35240
LOC_Os02g38090
LOC_Os02g42585
LOC_Os02g43790
LOC_Os02g43820
LOC_Os02g43940
LOC_Os02g43970
LOC_Os02g45420
LOC_Os02g45450
LOC_Os02g51670
LOC_Os02g52670
LOC_Os02g54050
LOC_Os02g54160
LOC_Os02g55380
LOC_Os03g05590
LOC_Os03g07830
LOC_Os03g07940
LOC_Os03g08460
LOC_Os03g08470
LOC_Os03g08490
LOC_Os03g08500
LOC_Os03g09170
LOC_Os03g15660
LOC_Os03g22170
LOC_Os03g60120
LOC_Os03g64260
LOC_Os04g18650
LOC_Os04g32620
LOC_Os04g32790
LOC_Os04g34970
LOC_Os04g44670
LOC_Os04g46220
LOC_Os04g46240
LOC_Os04g46250
LOC_Os04g46400
LOC_Os04g46410
LOC_Os04g46440
LOC_Os04g48350
LOC_Os04g52090
LOC_Os04g55520
LOC_Os04g56150
LOC_Os04g57340
LOC_Os05g25260
LOC_Os05g27930
LOC_Os05g28350
LOC_Os05g29810
LOC_Os05g34730
LOC_Os05g36100
LOC_Os05g37640
LOC_Os05g39590
LOC_Os05g41760
LOC_Os05g41780
LOC_Os05g49010
LOC_Os05g49700
LOC_Os06g03670
LOC_Os06g06540
LOC_Os06g06970
LOC_Os06g07030
LOC_Os06g08340
LOC_Os06g09390
LOC_Os06g09717
LOC_Os06g09760
LOC_Os06g09790
LOC_Os06g09810
LOC_Os06g10780
LOC_Os06g11860
LOC_Os06g11940
LOC_Os06g36000
LOC_Os06g40150
LOC_Os06g42990
LOC_Os06g44750
LOC_Os06g47590
LOC_Os07g03250
LOC_Os07g10410
LOC_Os07g12510
LOC_Os07g22730
LOC_Os07g22770
LOC_Os07g38750
LOC_Os07g42510
LOC_Os07g47330
LOC_Os07g47790
LOC_Os08g07700
LOC_Os08g27220
LOC_Os08g31580
LOC_Os08g34360
LOC_Os08g35240
LOC_Os08g36920
LOC_Os08g41030
LOC_Os08g42550
LOC_Os08g43200
LOC_Os08g43210
LOC_Os08g44960
LOC_Os08g45110
LOC_Os09g11460
LOC_Os09g11480
LOC_Os09g13940
LOC_Os09g20350
LOC_Os09g26420
LOC_Os09g28440
LOC_Os09g35010
LOC_Os09g35020
LOC_Os09g35030
LOC_Os09g39810
LOC_Os09g39850
LOC_Os10g22600
LOC_Os10g25170
LOC_Os10g26590
LOC_Os10g30840
LOC_Os10g38000
LOC_Os10g41130
LOC_Os10g41330
LOC_Os11g06770
LOC_Os11g13840
LOC_Os12g39330
LOC_Os12g40960
LOC_Os12g41030
LOC_Os12g41060
LOC_Os01g10580
LOC_Os02g39360
LOC_Os02g43170
LOC_Os04g41560
LOC_Os04g45690
LOC_Os05g11510
LOC_Os06g05890
LOC_Os06g49880
LOC_Os09g35880
LOC_Os12g10660
LOC_Os02g08150
LOC_Os02g39710
LOC_Os02g49230
LOC_Os02g49880
LOC_Os03g50310
LOC_Os04g42020
LOC_Os06g15330
LOC_Os06g16370
LOC_Os06g19444
LOC_Os06g44450
LOC_Os07g47140
LOC_Os08g15050
LOC_Os08g42440
LOC_Os09g06464
LOC_Os09g33550
LOC_Os01g67770
LOC_Os02g08500
LOC_Os02g55320
LOC_Os03g12350
LOC_Os04g28130
LOC_Os04g28160
LOC_Os05g32890
LOC_Os06g08440
LOC_Os06g43910
LOC_Os01g08160
LOC_Os01g13740
LOC_Os01g20700
LOC_Os01g62660
LOC_Os01g74020
LOC_Os02g04640
LOC_Os02g07170
LOC_Os02g07770
LOC_Os02g14490
LOC_Os02g22020
LOC_Os02g45080
LOC_Os02g46940
LOC_Os02g47190
LOC_Os03g03760
LOC_Os03g20900
LOC_Os03g21240
LOC_Os03g45194
LOC_Os03g55590
LOC_Os03g55760
LOC_Os04g47890
LOC_Os04g56990
LOC_Os05g32880
LOC_Os05g34110
LOC_Os05g40960
LOC_Os05g41240
LOC_Os06g24070
LOC_Os06g35140
LOC_Os06g40710
LOC_Os06g45410
LOC_Os06g45890
LOC_Os06g49040
LOC_Os07g02800
LOC_Os07g25710
LOC_Os07g48596
LOC_Os08g06370
LOC_Os08g25799
LOC_Os08g25820
LOC_Os08g33050
LOC_Os08g33750
LOC_Os09g12750
LOC_Os09g12770
LOC_Os09g23200
LOC_Os10g32600
LOC_Os10g39550
LOC_Os11g01480
LOC_Os12g01490
LOC_Os12g39640
LOC_Os01g10504
LOC_Os01g52680
LOC_Os01g66030
LOC_Os01g66290
LOC_Os02g01355
LOC_Os02g01365
LOC_Os02g07430
LOC_Os02g36924
LOC_Os02g45770
LOC_Os02g49840
LOC_Os02g52340
LOC_Os03g08754
LOC_Os03g11614
LOC_Os03g54160
LOC_Os03g54170
LOC_Os04g23910
LOC_Os04g49150
LOC_Os04g52410
LOC_Os05g11380
LOC_Os05g11414
LOC_Os05g34940
LOC_Os06g01890
LOC_Os06g06750
LOC_Os06g11330
LOC_Os06g45650
LOC_Os06g49840
LOC_Os07g01820
LOC_Os07g41370
LOC_Os08g02070
LOC_Os08g33488
LOC_Os08g41950
LOC_Os08g41960
LOC_Os09g32948
LOC_Os10g39130
LOC_Os12g10520
LOC_Os12g10540
LOC_Os12g31748
LOC_Os01g11510
LOC_Os01g18420
LOC_Os01g18440
LOC_Os01g23750
LOC_Os01g23760
LOC_Os01g23770
LOC_Os01g23780
LOC_Os01g67890
LOC_Os01g68560
LOC_Os01g69850
LOC_Os01g74440
LOC_Os02g06860
LOC_Os03g03100
LOC_Os03g14850
LOC_Os03g37670
LOC_Os03g38610
LOC_Os04g25870
LOC_Os04g31804
LOC_Os04g38770
LOC_Os05g23780
LOC_Os06g11970
LOC_Os06g22760
LOC_Os06g23950
LOC_Os06g30810
LOC_Os06g30830
LOC_Os07g04170
LOC_Os08g38590
LOC_Os09g02780
LOC_Os09g02830
LOC_Os11g12360
LOC_Os11g43740
LOC_Os12g21850
LOC_Os12g21880
LOC_Os02g45570
LOC_Os02g47280
LOC_Os02g53690
LOC_Os03g47140
LOC_Os03g51970
LOC_Os04g48510
LOC_Os04g51190
LOC_Os06g02560
LOC_Os06g10310
LOC_Os07g28430
LOC_Os11g35030
LOC_Os12g29980
LOC_Os01g48170
LOC_Os01g70810
LOC_Os02g05450
LOC_Os03g55990
LOC_Os05g09630
LOC_Os05g25600
LOC_Os05g48820
LOC_Os05g50130
LOC_Os06g29020
LOC_Os06g39906
LOC_Os07g24350
LOC_Os08g32990
LOC_Os10g39720
LOC_Os11g01130
LOC_Os12g01120
LOC_Os05g02730
LOC_Os04g55590
LOC_Os01g62310
LOC_Os04g56780
LOC_Os01g63510
LOC_Os01g47710
LOC_Os05g48990
LOC_Os07g48560
LOC_Os03g20910
LOC_Os07g34880
LOC_Os08g14400
LOC_Os01g60270
LOC_Os12g43950
LOC_Os03g47740
LOC_Os03g52239
LOC_Os11g06020
LOC_Os03g06930
LOC_Os06g01934
LOC_Os01g62920
LOC_Os10g39030
LOC_Os05g38120
LOC_Os03g03260
LOC_Os03g47730
LOC_Os02g13310
LOC_Os06g36680
LOC_Os08g37580
LOC_Os02g49700
LOC_Os08g32080
LOC_Os08g32085
LOC_Os09g29460
LOC_Os03g07450
LOC_Os04g46350
LOC_Os08g36220
LOC_Os03g08960
LOC_Os10g26500
LOC_Os09g27450
LOC_Os10g41230
LOC_Os10g01470
LOC_Os02g35770
LOC_Os10g23090
LOC_Os04g45810
LOC_Os06g04850
LOC_Os03g12860
LOC_Os06g04870
LOC_Os02g43330
LOC_Os09g35910
LOC_Os09g21180
LOC_Os01g45570
LOC_Os06g48290
LOC_Os07g39320
LOC_Os02g05640
LOC_Os03g10210
LOC_Os03g51690
LOC_Os03g56110
LOC_Os03g47016
LOC_Os03g47036
LOC_Os07g03770
LOC_Os01g19694
LOC_Os05g03884
LOC_Os03g51710
LOC_Os06g43860
LOC_Os02g08544
LOC_Os08g19650
LOC_Os03g56140
LOC_Os03g03164
LOC_Os08g08820
LOC_Os04g53540
LOC_Os08g04190
LOC_Os02g45250
LOC_Os09g35760
LOC_Os03g43930
LOC_Os01g55549
LOC_Os12g41860
LOC_Os03g01890
LOC_Os04g48070
LOC_Os10g33960
LOC_Os10g42490
LOC_Os06g10600
LOC_Os08g19590
LOC_Os01g57890
LOC_Os06g12400
LOC_Os01g07930
LOC_Os01g09620
LOC_Os01g09850
LOC_Os01g14010
LOC_Os01g14870
LOC_Os01g15350
LOC_Os01g15460
LOC_Os01g39100
LOC_Os01g39110
LOC_Os01g42710
LOC_Os01g42970
LOC_Os01g45730
LOC_Os01g53650
LOC_Os01g57020
LOC_Os01g57650
LOC_Os01g62130
LOC_Os01g62190
LOC_Os01g63980
LOC_Os01g65080
LOC_Os01g66570

LOC_Os10g40700
LOC_Os10g41100
LOC_Os10g41330
LOC_Os10g41710
LOC_Os10g41749
LOC_Os10g42040
LOC_Os10g42100
LOC_Os10g42299
LOC_Os10g42960
LOC_Os11g01550
LOC_Os11g02070
LOC_Os11g02240
LOC_Os11g02330
LOC_Os11g02350
LOC_Os11g02450
LOC_Os11g04020
LOC_Os11g04030
LOC_Os11g04190
LOC_Os11g04850
LOC_Os11g05170
LOC_Os11g05410
LOC_Os11g05700
LOC_Os11g05990
LOC_Os11g06150
LOC_Os11g07020
LOC_Os11g07922
LOC_Os11g08200
LOC_Os11g11000
LOC_Os11g12470
LOC_Os11g13860
LOC_Os11g13980
LOC_Os11g15624
LOC_Os11g19730
LOC_Os11g20040
LOC_Os11g20160
LOC_Os11g20689
LOC_Os11g26030
LOC_Os11g26340
LOC_Os11g26570
LOC_Os11g26750
LOC_Os11g26760
LOC_Os11g26780
LOC_Os11g26790
LOC_Os11g27329
LOC_Os11g29140
LOC_Os11g29470
LOC_Os11g29780
LOC_Os11g30350
LOC_Os11g31060
LOC_Os11g32260
LOC_Os11g32580
LOC_Os11g32650
LOC_Os11g32880
LOC_Os11g33270
LOC_Os11g37950
LOC_Os11g37960
LOC_Os11g38180
LOC_Os11g38220
LOC_Os11g38810
LOC_Os11g40600
LOC_Os11g40930
LOC_Os11g41850
LOC_Os11g42200
LOC_Os11g42370
LOC_Os11g43520
LOC_Os11g43830
LOC_Os11g44420
LOC_Os11g45710
LOC_Os11g46000
LOC_Os11g47520
LOC_Os11g47600
LOC_Os11g47610
LOC_Os12g02000
LOC_Os12g02200
LOC_Os12g02300
LOC_Os12g02570
LOC_Os12g03050
LOC_Os12g03594
LOC_Os12g03860
LOC_Os12g03990
LOC_Os12g04740
LOC_Os12g05210
LOC_Os12g05360
LOC_Os12g05540
LOC_Os12g05600
LOC_Os12g05640
LOC_Os12g05840
LOC_Os12g05890
LOC_Os12g07030
LOC_Os12g07710
LOC_Os12g07950
LOC_Os12g08700
LOC_Os12g08810
LOC_Os12g10220
LOC_Os12g10540
LOC_Os12g11660
LOC_Os12g12560
LOC_Os12g12580
LOC_Os12g12600
LOC_Os12g12860
LOC_Os12g12934
LOC_Os12g13890
LOC_Os12g14440
LOC_Os12g15680
LOC_Os12g16010
LOC_Os12g17320
LOC_Os12g18410
LOC_Os12g18450
LOC_Os12g19381
LOC_Os12g19470
LOC_Os12g24320
LOC_Os12g25090
LOC_Os12g25200
LOC_Os12g26220
LOC_Os12g26290
LOC_Os12g27254
LOC_Os12g28770
LOC_Os12g29330
LOC_Os12g30824
LOC_Os12g31000
LOC_Os12g31460
LOC_Os12g31860
LOC_Os12g32760
LOC_Os12g33210
LOC_Os12g33300
LOC_Os12g35340
LOC_Os12g35670
LOC_Os12g36040
LOC_Os12g36194
LOC_Os12g36850
LOC_Os12g36890
LOC_Os12g38010
LOC_Os12g38051
LOC_Os12g38270
LOC_Os12g38850
LOC_Os12g39360
LOC_Os12g39630
LOC_Os12g41590
LOC_Os12g43380
LOC_Os12g43430
LOC_Os12g43720

LOC_Os01g67970
LOC_Os01g68160
LOC_Os01g68860
LOC_Os01g70870
LOC_Os02g01090
LOC_Os02g02424
LOC_Os02g06584
LOC_Os02g08510
LOC_Os02g10080
LOC_Os02g19804
LOC_Os02g31890
LOC_Os02g34680
LOC_Os02g35460
LOC_Os02g36360
LOC_Os02g44120
LOC_Os02g44130
LOC_Os02g45054
LOC_Os02g45480
LOC_Os02g57550
LOC_Os02g57790
LOC_Os02g58440
LOC_Os03g02160
LOC_Os03g05480
LOC_Os03g05690
LOC_Os03g10140
LOC_Os03g13400
LOC_Os03g13600
LOC_Os03g15790
LOC_Os03g17150
LOC_Os03g18950
LOC_Os03g31240
LOC_Os03g32220
LOC_Os03g32230
LOC_Os03g43840
LOC_Os03g49132
LOC_Os03g49170
LOC_Os03g55540
LOC_Os03g60560
LOC_Os03g60570
LOC_Os03g62230
LOC_Os04g01480
LOC_Os04g02510
LOC_Os04g02730
LOC_Os04g08060
LOC_Os04g08290
LOC_Os04g08600
LOC_Os04g35800
LOC_Os04g36650
LOC_Os04g39050
LOC_Os04g39520
LOC_Os04g41060
LOC_Os04g46670
LOC_Os04g46680
LOC_Os04g47860
LOC_Os04g50070
LOC_Os04g57010
LOC_Os04g57600
LOC_Os04g59380
LOC_Os05g01550
LOC_Os05g02390
LOC_Os05g03020
LOC_Os05g03760
LOC_Os05g10670
LOC_Os05g14130
LOC_Os05g20930
LOC_Os05g37190
LOC_Os05g38600
LOC_Os05g38620
LOC_Os05g41790
LOC_Os05g45020
LOC_Os05g50080
LOC_Os05g51830
LOC_Os06g07020
LOC_Os06g10470
LOC_Os06g20020
LOC_Os06g21390
LOC_Os06g32720
LOC_Os06g32860
LOC_Os06g40960
LOC_Os06g41384
LOC_Os06g46400
LOC_Os06g46890
LOC_Os06g49080
LOC_Os06g51140
LOC_Os07g01180
LOC_Os07g04580
LOC_Os07g04650
LOC_Os07g23450
LOC_Os07g28910
LOC_Os07g38090
LOC_Os07g38240
LOC_Os07g39310
LOC_Os07g39970
LOC_Os07g40080
LOC_Os07g40780
LOC_Os07g40950
LOC_Os07g44640
LOC_Os08g03610
LOC_Os08g04170
LOC_Os08g06280
LOC_Os08g06330
LOC_Os08g17640
LOC_Os08g20580
LOC_Os08g36390
LOC_Os08g37904
LOC_Os08g37920
LOC_Os08g38370
LOC_Os08g39390
LOC_Os08g44050
LOC_Os08g44190
LOC_Os08g44830
LOC_Os09g03500
LOC_Os09g10980
LOC_Os09g13530
LOC_Os09g13680
LOC_Os09g26210
LOC_Os09g27650
LOC_Os09g31140
LOC_Os09g38340
LOC_Os09g38610
LOC_Os09g39660
LOC_Os10g07080
LOC_Os10g25220
LOC_Os10g28330
LOC_Os11g06840
LOC_Os11g25610
LOC_Os11g28270
LOC_Os11g30484
LOC_Os11g47620
LOC_Os11g47630
LOC_Os11g48000
LOC_Os12g03554
LOC_Os12g07280
LOC_Os12g18120
LOC_Os12g18150
LOC_Os12g21700
LOC_Os12g33090
LOC_Os12g38940
LOC_Os12g38960
LOC_Os12g39400
LOC_Os12g41700
LOC_Os01g03720
LOC_Os01g04930
LOC_Os01g07450
LOC_Os01g09590
LOC_Os01g12860
LOC_Os01g16810
LOC_Os01g18240
LOC_Os01g19330
LOC_Os01g19970
LOC_Os01g36460
LOC_Os01g45090
LOC_Os01g49160
LOC_Os01g50110
LOC_Os01g50720
LOC_Os01g51260
LOC_Os01g52410
LOC_Os01g59660
LOC_Os01g62410
LOC_Os01g63160
LOC_Os01g63460
LOC_Os01g63680
LOC_Os01g64360
LOC_Os01g65370
LOC_Os01g74410
LOC_Os01g74590
LOC_Os02g02370
LOC_Os02g09480
LOC_Os02g17190
LOC_Os02g36890
LOC_Os02g40530
LOC_Os02g41510
LOC_Os02g42850
LOC_Os02g42870
LOC_Os02g46780
LOC_Os02g49250
LOC_Os02g49986
LOC_Os02g51799
LOC_Os02g54520
LOC_Os03g04900
LOC_Os03g13310
LOC_Os03g18480
LOC_Os03g19120
LOC_Os03g20090
LOC_Os03g25550
LOC_Os03g26130
LOC_Os03g27090
LOC_Os03g29614
LOC_Os03g38210
LOC_Os03g51110
LOC_Os03g56090
LOC_Os04g28090
LOC_Os04g30890
LOC_Os04g38740
LOC_Os04g39470
LOC_Os04g42950
LOC_Os04g43680
LOC_Os04g45020
LOC_Os04g45060
LOC_Os04g46384
LOC_Os04g50680
LOC_Os04g50770
LOC_Os05g04210
LOC_Os05g04820
LOC_Os05g28320
LOC_Os05g35500
LOC_Os05g37060
LOC_Os05g37730
LOC_Os05g38460
LOC_Os05g41166
LOC_Os05g46610
LOC_Os05g48010
LOC_Os05g49310
LOC_Os06g02250
LOC_Os06g06740
LOC_Os06g10350
LOC_Os06g11780
LOC_Os06g14670
LOC_Os06g14700
LOC_Os06g40330
LOC_Os06g43090
LOC_Os06g46560
LOC_Os07g04700
LOC_Os07g12130
LOC_Os07g14110
LOC_Os07g25370
LOC_Os07g31470
LOC_Os07g37210
LOC_Os07g43420
LOC_Os07g43580
LOC_Os07g44090
LOC_Os07g48870
LOC_Os08g05520
LOC_Os08g15020
LOC_Os08g33150
LOC_Os08g33660
LOC_Os08g33800
LOC_Os08g33940
LOC_Os08g34960
LOC_Os08g37970
LOC_Os08g43550
LOC_Os09g01960
LOC_Os09g23620
LOC_Os09g24800
LOC_Os09g26170
LOC_Os09g36250
LOC_Os09g36730
LOC_Os10g20990
LOC_Os10g33810
LOC_Os10g35660
LOC_Os11g03440
LOC_Os11g10130
LOC_Os11g35390
LOC_Os11g45740
LOC_Os11g47460
LOC_Os12g03150
LOC_Os12g07610
LOC_Os12g07640
LOC_Os12g13570
LOC_Os12g33070
LOC_Os12g37690
LOC_Os12g37970
LOC_Os12g38400
LOC_Os01g06320
LOC_Os01g07430
LOC_Os01g09280
LOC_Os01g09640
LOC_Os01g09760
LOC_Os01g11200
LOC_Os01g12700
LOC_Os01g34060
LOC_Os01g40670
LOC_Os01g41900
LOC_Os01g43180
LOC_Os01g43230
LOC_Os01g44370
LOC_Os01g47370
LOC_Os01g51154
LOC_Os02g30700
LOC_Os02g34630
LOC_Os02g45670
LOC_Os02g46030
LOC_Os02g47744
LOC_Os02g53670
LOC_Os03g13790
LOC_Os03g14810
LOC_Os03g19630
LOC_Os03g25304
LOC_Os03g31230
LOC_Os03g53960
LOC_Os03g62100
LOC_Os03g62379
LOC_Os03g63890
LOC_Os04g27410
LOC_Os04g47750
LOC_Os04g49450
LOC_Os04g58020
LOC_Os05g02420
LOC_Os05g03550
LOC_Os05g07010
LOC_Os05g10690
LOC_Os05g37050
LOC_Os05g49240
LOC_Os05g50340
LOC_Os05g50350
LOC_Os05g51160
LOC_Os06g01670
LOC_Os06g04960
LOC_Os06g07640
LOC_Os06g07650
LOC_Os06g08290
LOC_Os06g14010
LOC_Os06g14710
LOC_Os06g19980
LOC_Os06g45840
LOC_Os06g51260
LOC_Os07g25150
LOC_Os07g26150
LOC_Os07g30130
LOC_Os07g49530
LOC_Os08g04840
LOC_Os08g05510
LOC_Os08g06110
LOC_Os08g06240
LOC_Os08g39980
LOC_Os09g31454
LOC_Os10g30690
LOC_Os10g30719
LOC_Os10g41200
LOC_Os10g41260
LOC_Os12g33950
LOC_Os12g41920
